# Recent Advances in Macrocyclic Drugs and Microwave-Assisted and/or Solid-Supported Synthesis of Macrocycles

**DOI:** 10.3390/molecules27031012

**Published:** 2022-02-02

**Authors:** Dianqing Sun

**Affiliations:** Department of Pharmaceutical Sciences, The Daniel K. Inouye College of Pharmacy, University of Hawaii at Hilo, Hilo, HI 96720, USA; dianqing@hawaii.edu; Tel.: +1-808-932-8122

**Keywords:** high throughput chemistry, microwave, macrocyclization, macrocycle, macrocyclic drugs, solid-phase

## Abstract

Macrocycles represent attractive candidates in organic synthesis and drug discovery. Since 2014, nineteen macrocyclic drugs, including three radiopharmaceuticals, have been approved by FDA for the treatment of bacterial and viral infections, cancer, obesity, immunosuppression, etc. As such, new synthetic methodologies and high throughput chemistry (e.g., microwave-assisted and/or solid-phase synthesis) to access various macrocycle entities have attracted great interest in this chemical space. This article serves as an update on our previous review related to macrocyclic drugs and new synthetic strategies toward macrocycles (*Molecules*, **2013**, *18*, 6230). In this work, I first reviewed recent FDA-approved macrocyclic drugs since 2014, followed by new advances in macrocycle synthesis using high throughput chemistry, including microwave-assisted and/or solid-supported macrocyclization strategies. Examples and highlights of macrocyclization include macrolactonization and macrolactamization, transition-metal catalyzed olefin ring-closure metathesis, intramolecular C–C and C–heteroatom cross-coupling, copper- or ruthenium-catalyzed azide–alkyne cycloaddition, intramolecular S_N_Ar or S_N_2 nucleophilic substitution, condensation reaction, and multi-component reaction-mediated macrocyclization, and covering the literature since 2010.

## 1. Introduction and Recently Approved Macrocyclic Drugs

Macrocycles continue to serve as an important class of compounds and have had a profound impact on chemistry, biology, and medicine [1,2,3]. Because of their cyclic nature, conformational and configurational characteristics, along with template-induced preorganization associated with macrocycles [4], compared to small molecule drugs and large biologics, macrocycles can offer unique drug-like profiles such as favorable pharmacokinetic and pharmacodynamic (PK/PD) parameters and improved oral bioavailability, enhanced metabolic stability and cell permeability, increased binding affinity, and desirable conformational rigidity [1,5]. As such, new synthetic strategies and structure–activity relationship (SAR) studies of macrocyclic chemical entities and related natural products remain an attractive research area in this field [6,7,8].

**Figure 1:**
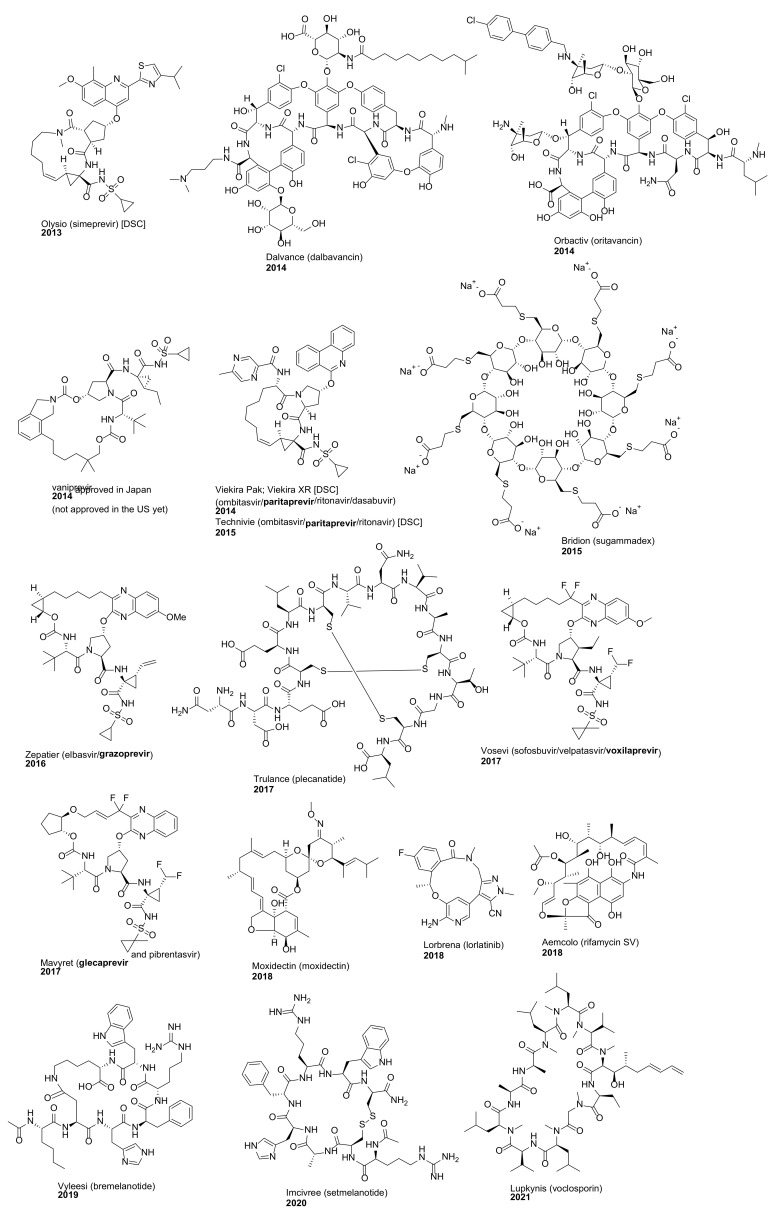
Macrocyclic drugs approved by the US FDA since 2014.

**Figure 2:**
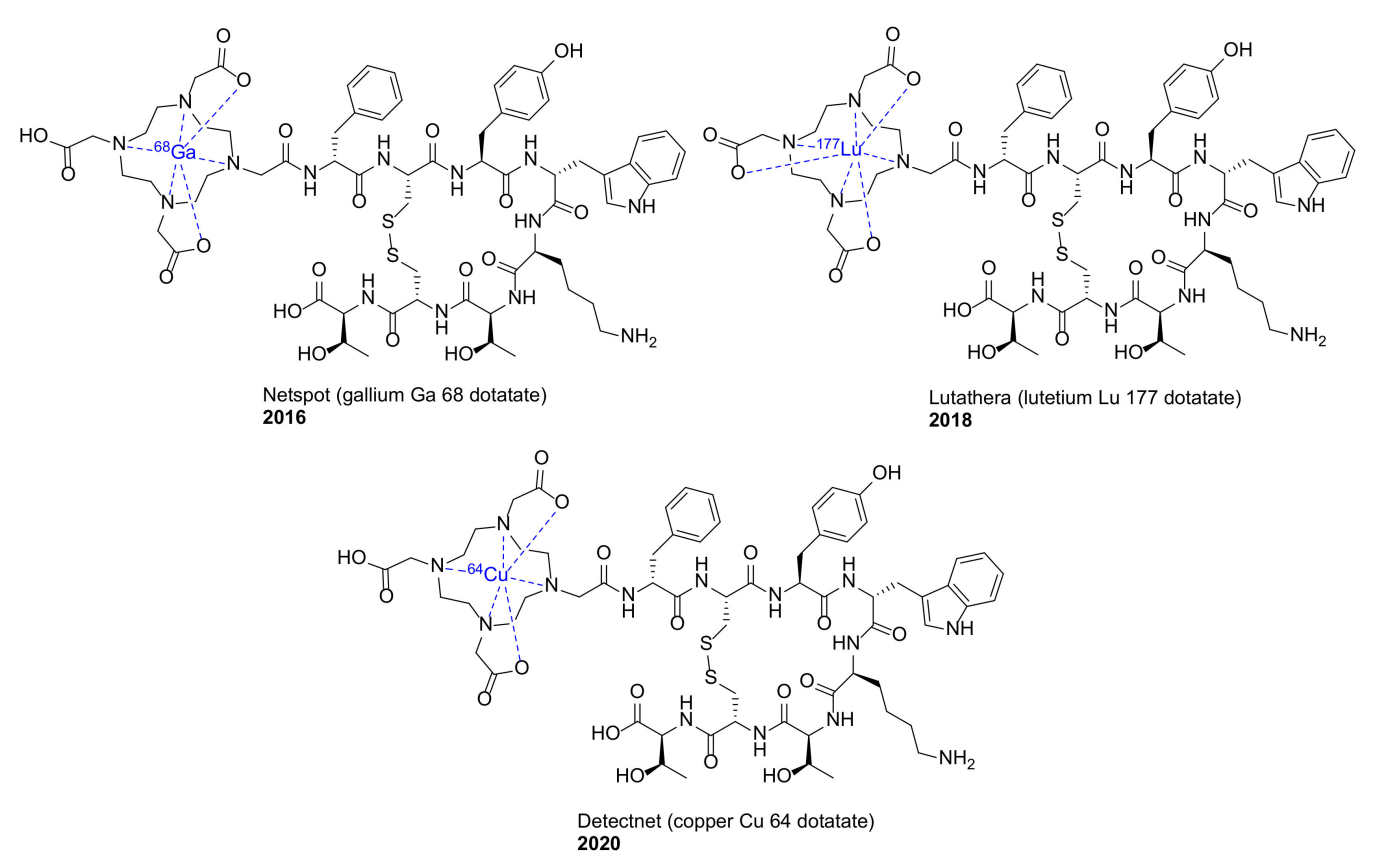
Macrocyclic radiopharmaceuticals approved by the US FDA since 2014.

We previously reviewed macrocyclic drugs and general synthetic strategies toward macrocycles [1]. Since 2014, a number of new macrocyclic drugs have been approved by the US Food and Drug Administration (FDA), and their chemical structures, brand, and generic drug names, and the year of the approval are shown in Figure 1 and Figure 2. These include antibacterial agents Dalvance (dalbavancin) [9], Orbactiv or Kimyrsa (oritavancin) [10,11] and Aemcolo (rifamycin SV) [12,13]; antiviral agents Olysio (simeprevir) [14], vaniprevir, Viekira Pak or Viekira XR (ombitasvir/paritaprevir/ritonavir) [15], Technivie (ombitasvir/paritaprevir/ritonavir/dasabuvir) [16], Vosevi (sofosbuvir/velpatasvir/voxilaprevir) [17], Zepatier (elbasvir/grazoprevir) [18], and Mavyret (glecaprevir/pibrentasvir) [19], for the treatment of hepatitis C virus (HCV) infection; antidote and reversal agent Bridion (sugammadex) for neuromuscular blockade induced by rocuronium bromide and vecuronium bromide [20]; guanylate cyclase-C agonist Trulance (plecanatide) for the treatment of chronic idiopathic constipation (CIC) and irritable bowel syndrome with constipation (IBS-C) [21]; anthelmintic moxidectin [22]; ancancer agent Lorbrena (lorlatinib) for the treatment of anaplastic lymphoma kinase (ALK)-positive metastatic non-small cell lung cancer (NSCLC) [23]; Vyleesi (bremelanotide) for the treatment of hypoactive sexual desire disorder [24]; Imcivree (setmelanotide) for chronic weight management [25]; and immunosuppressant Lupkynis (voclosporin) [26]. In addition, several macrocyclic dotatate-based radiopharmaceuticals have also been approved, such as diagnostic imaging agents Netspot (gallium Ga 68 dotatate) [27] and Detectnet (copper Cu 64 dotatate) for the detection of rare neuroendocrine tumors [28], along with Lutathera (lutetium Lu 177 dotatate) for the treatment of somatostatin receptor-positive gastroenteropancreatic neuroendocrine tumors (GEP-NETs) [29]. Their detailed physiochemical properties and PK/PD parameters of these newly FDA approved macrocyclic drugs are summarized in Table 1.

Furthermore, this review discusses and highlights new advances in macrocycle synthesis using high throughput chemistry, including microwave (MW)-assisted and/or solid-supported macrocyclization, and covering the literature since 2010. Microwave and solid-phase syntheses have played an important role in enhancing chemical synthesis efficiency, such as decreasing reaction time, improving reaction yield and product purity, and streamlining the workup and purification process [30,31]. These enabling and accelerating technologies also play an integral role in organic chemistry, chemical biology, and drug discovery. New synthetic methods were broken down into macrolactonization and macrolactamization, transition-metal catalyzed olefin ring-closure metathesis, intramolecular C–C and C–heteroatom cross-coupling, copper- or ruthenium-catalyzed azide–alkyne cycloaddition, intramolecular S_N_Ar or S_N_2 nucleophilic substitution, condensation reaction, and multi-component reaction-mediated macrocyclization.

## 2. Microwave-Assisted and/or Solid-Supported Synthesis of Macrocycles

### 2.1. Macrolactonization and Macrolactamization

**Scheme 1:**
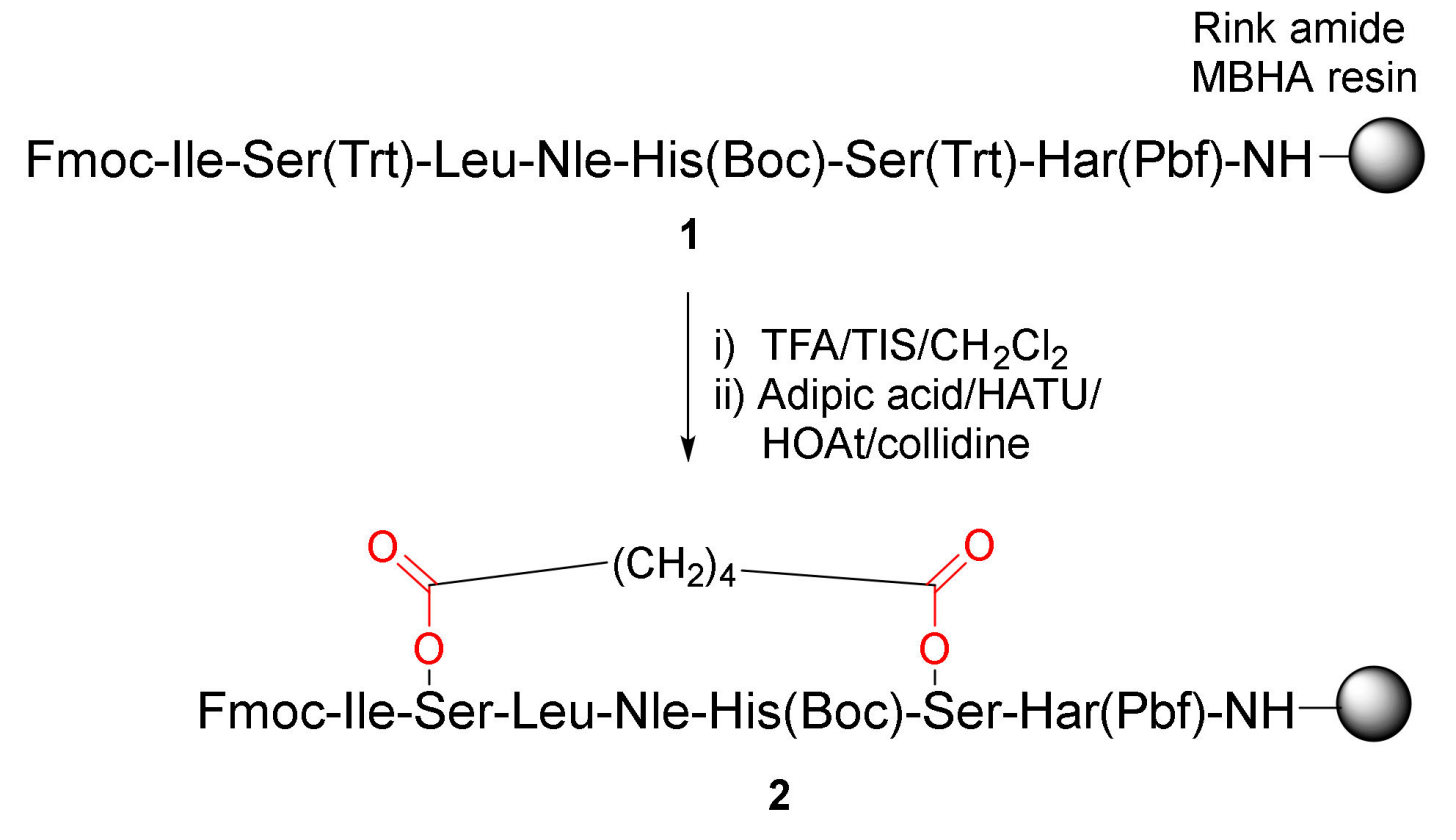
On-resin synthesis of a cyclic peptide **2** via side-chain diester cyclization strategy [35].

Caporale et al. reported the synthesis and evaluation of a novel, cyclic parathyroid hormone fragment analog via side-chain cyclization of serine residues in 2010 [35]. On-resin diester bridge formation of **1** was performed using adipic acid via acylation of the hydroxyl groups of Serines 6 and 10 (Scheme 1). Specifically, following selective deprotection of the trityl groups from the Ser residues in **1** with 1% TFA/CH2Cl2, solid-supported peptide **2** with a cross-linked side chain was obtained via esterification with adipic acid upon in situ activation in the presence of HATU/HOAt/collidine [35].

**Scheme 2:**
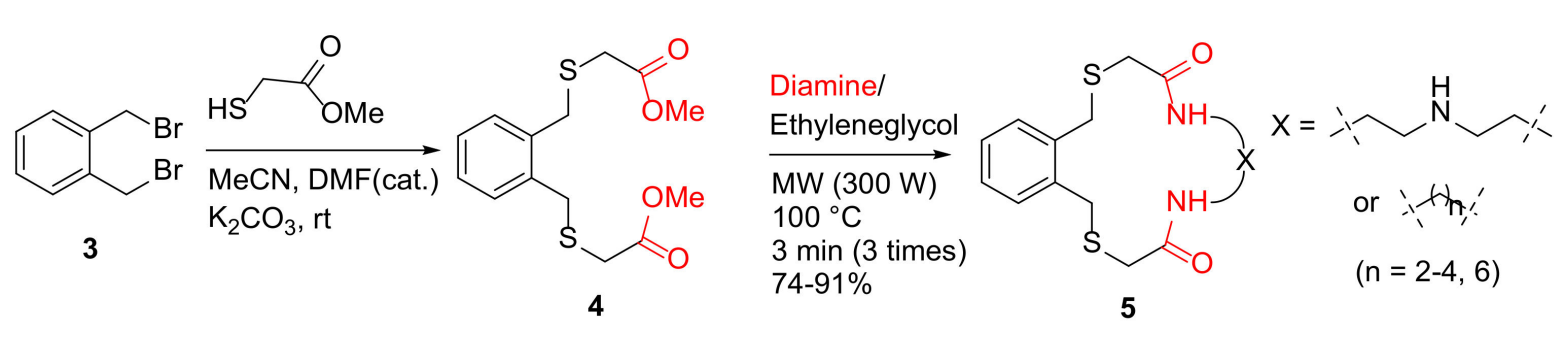
MW-assisted synthesis of aza thia crowns **5** [37].

In order to extend their previous work in the synthesis of aza crowns [36], in 2012, Rostami et al. reported the MW-assisted synthesis of new aza thia crowns [37]. Specifically, aza thia crowns **5** were synthesized from α, α-bis(bromomethyl) benzene **3**, and methylthioglycolate, followed by the reaction of this diester **4** and diamines in ethyleneglycol under MW irradiation at 100 °C for 9 min in 74–91% yields (Scheme 2). In contrast, conventional reflux in methanol for 24 h gave much lower yields (9–19%) [37].

**Scheme 3:**
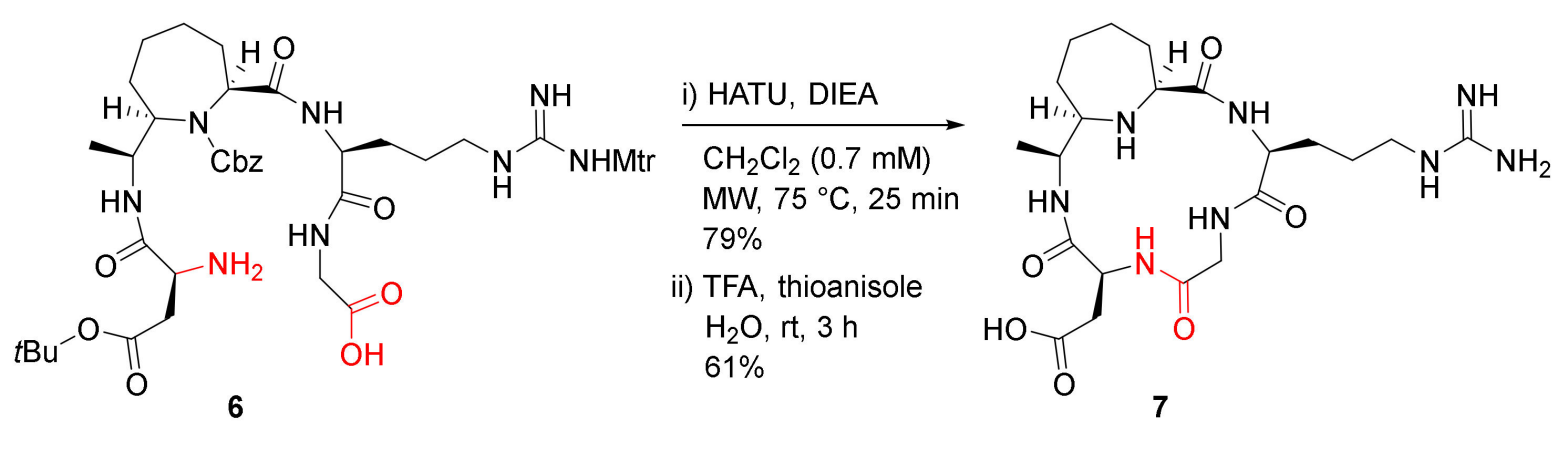
MW-assisted synthesis of a pentapeptide RGD analog **7** [38].

Cini et al. reported the MW-assisted synthesis of conformationally constrained peptidomimetics in 2012 [38]. The cyclopentapeptide analog **7** bearing an arginylglycylaspartic acid (RGD) motif, along with an enantiopure 7-substituted azepane-2-carboxylic acid (ACA) linker, was synthesized from a linear counterpart **6** in the presence of HATU/DIEA in dichloromethane (0.7 mM) at 75 °C under MW irradiation (25 W) for 25 min in 79% yield, followed by global side-chain deprotection in TFA/thioanisole/H_2_O (90/5/5) at room temperature for 3 h (Scheme 3) [38]. Macrocycle **7** showed low micromolar affinity towards α_ν_β_3_ and α_ν_β_5_ receptors with IC_50_ values of 1.8 and 2.9 µM, respectively [38].

**Scheme 4:**
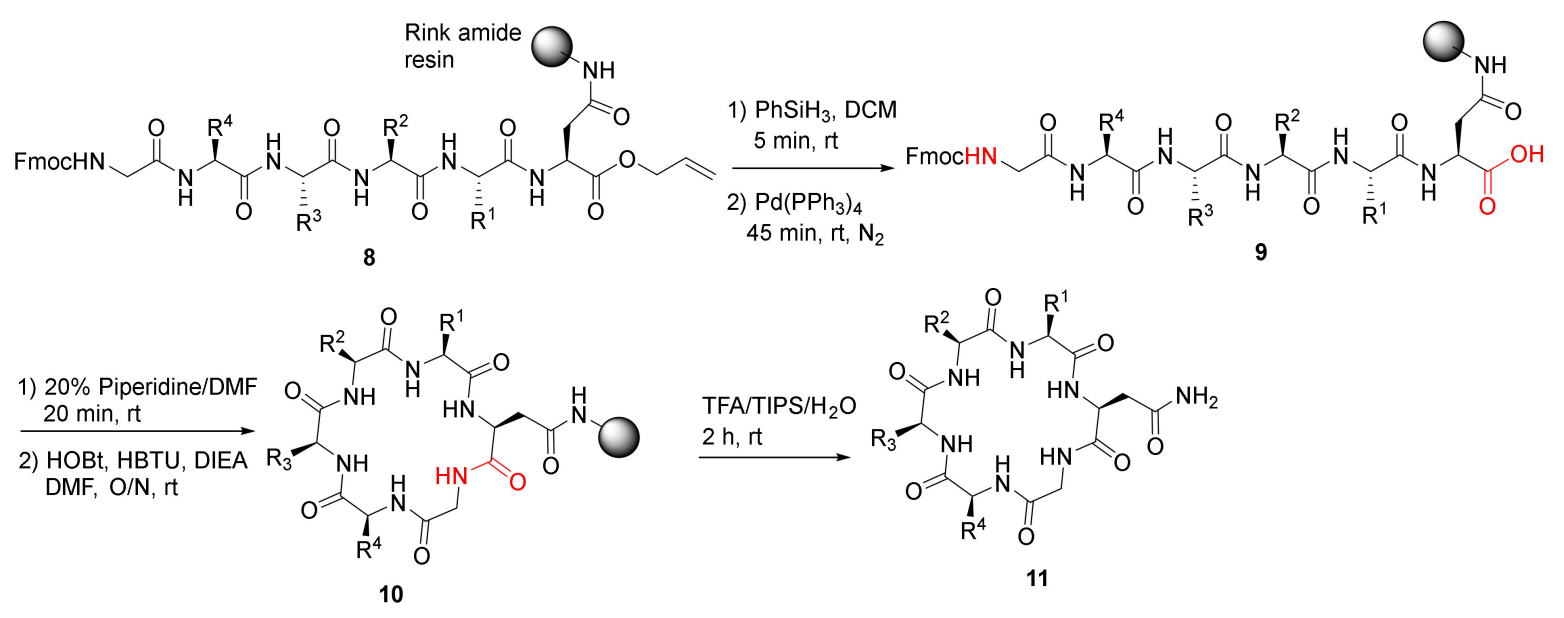
On-resin synthesis of cyclohexapeptides **11** [39].

Ferrie et al. reported the solid-phase synthesis and a comparative protease stability study of macrocyclic hexapeptides to mimic two endocrine hormones in 2013 [39]. Cyclohexapeptides **11** were synthesized using Rink resin and standard Fmoc amino acid coupling protocol, deprotection of the allyl group in **8**, on-resin macrolactamization of **9**, resin cleavage to provide **11** (Scheme 4) [39]. These endocrine peptide mimics showed improved stability profiles relative to naturally occurring vasopressin, oxytocin, and a linear control peptide.

**Scheme 5:**
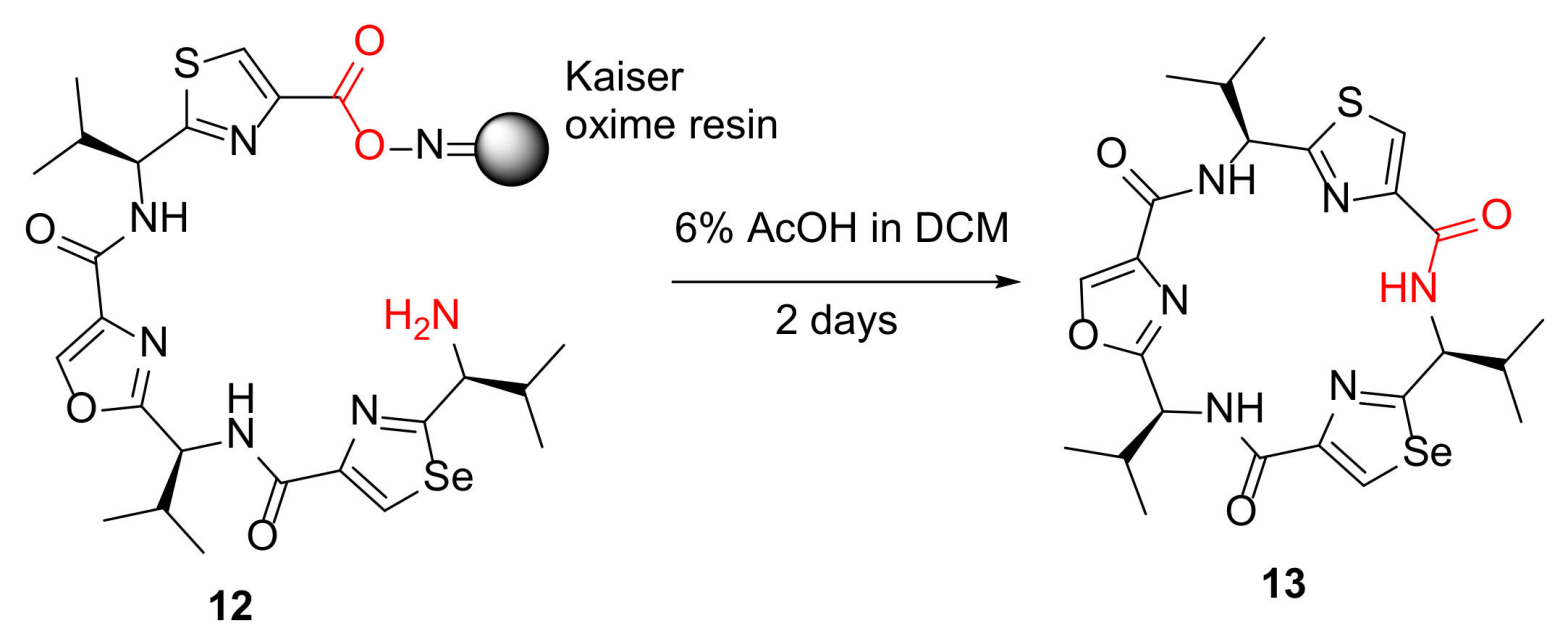
Synthesis of a representative azole-enriched cyclopeptide **13** via an on-resin cyclization–cleavage strategy [40].

Tao et al. reported the synthesis of azole cyclopeptide analogs via an on-resin cyclization–cleavage strategy in 2013 [40]. A selected example **13** from solid-supported linear precursor **12** is shown in Scheme 5, and using this developed solid-phase-based cyclitive–cleavage strategy, a chemical library was synthesized efficiently to produce >100 diverse azole cyclopeptide derivatives with various ring sizes, as modulators of multidrug resistance efflux pumps **[40]**. 

**Scheme 6:**
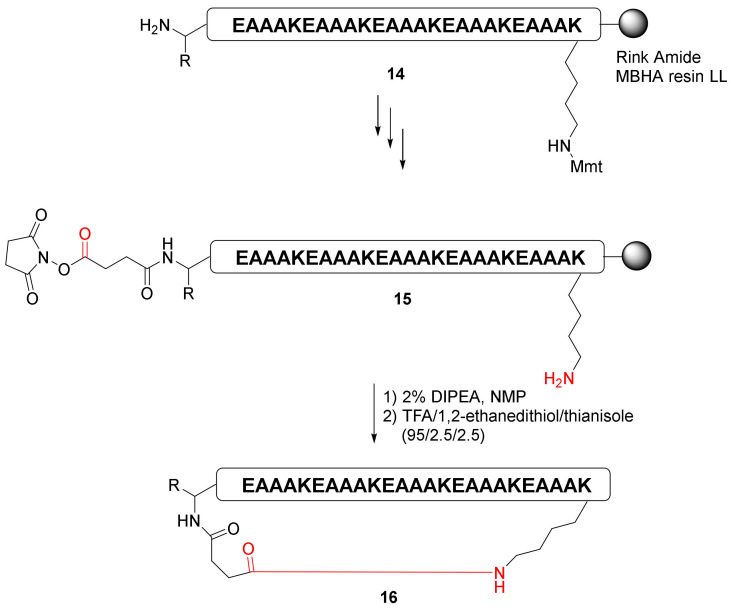
On-resin synthesis of cyclopeptides **16** via pre-activated *N*-hydroxysuccinimide (NHS) ester strategy **[41]**.

Choi et al. reported a highly efficient pre-activation cyclization of the long peptide via succinimidyl ester-amine reaction strategy in 2015 [41]. After the formation of a pre-activated succinimidyl ester precursor, on-resin macrocyclization of the 25 AA peptide **15** was achieved effectively to yield cyclopeptide **16** (Scheme 6) [41].

**Scheme 7:**
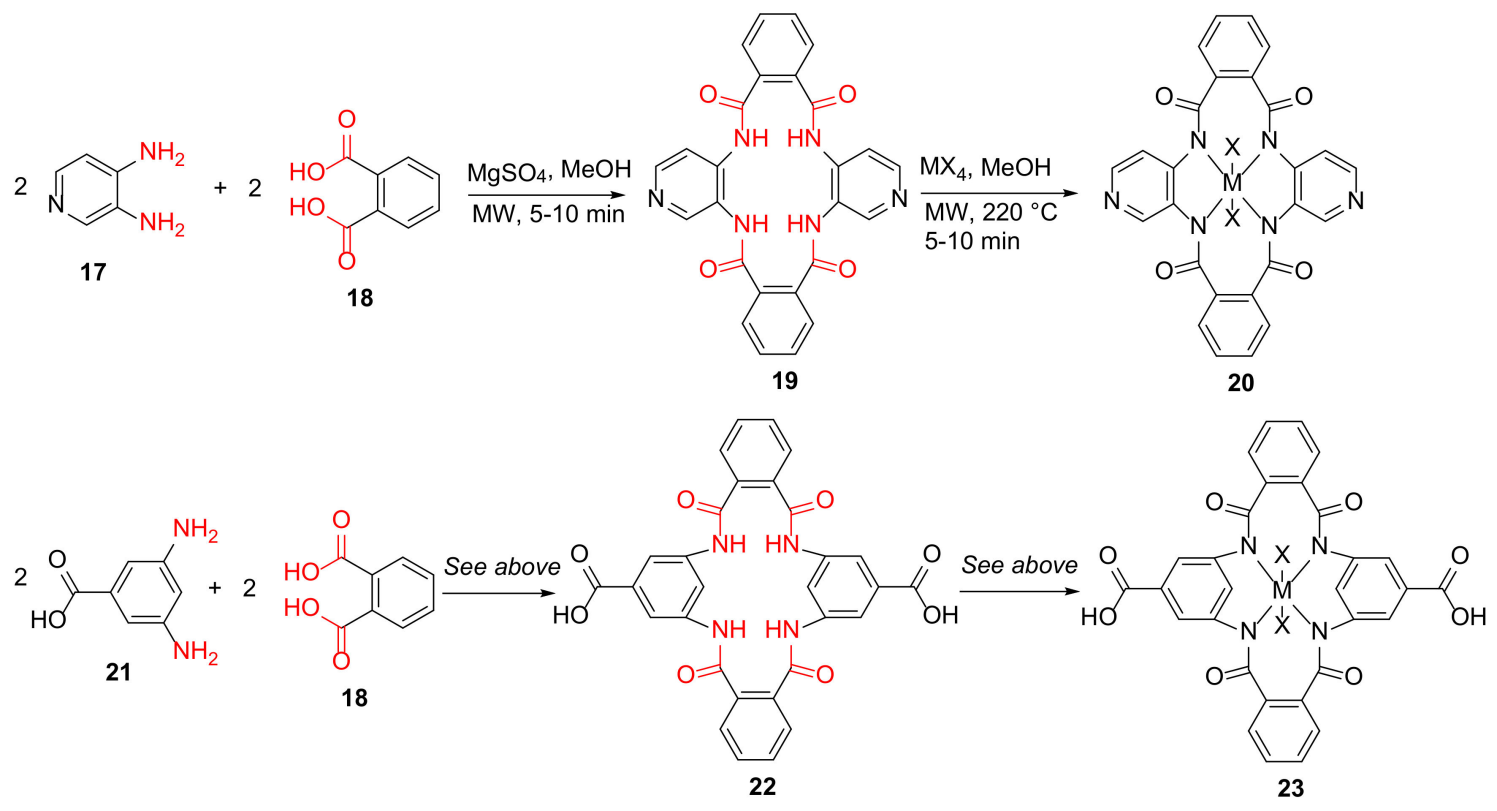
MW-assisted synthesis of 16/18-membered macrocycle metal complexes [42].

Kumar et al. reported the MW-assisted synthesis of the Ti/Zr(IV) metal complexes of 16/18-membered macrocycles as potential antibacterial and β-lactamase inhibitors in 2017 [42]. Specifically, **19** and **22** were synthesized from 3,4-diaminopyridine **17** or 3,5-diaminobenzoic acid **21** and phthalic acid **18** in the presence of anhydrous MgSO_4_ and methanol under MW irradiation for 5–10 min, followed by the formations of Ti or Zr(IV) metal complexes **20** and **23** of these macrocycle ligands (Scheme 7). The Zr(IV) macrocycle complex, **23**, showed good antibacterial activities against extended-spectrum β-lactamase (ESBL)-producing *E. coli* strains [42].

**Scheme 8:**
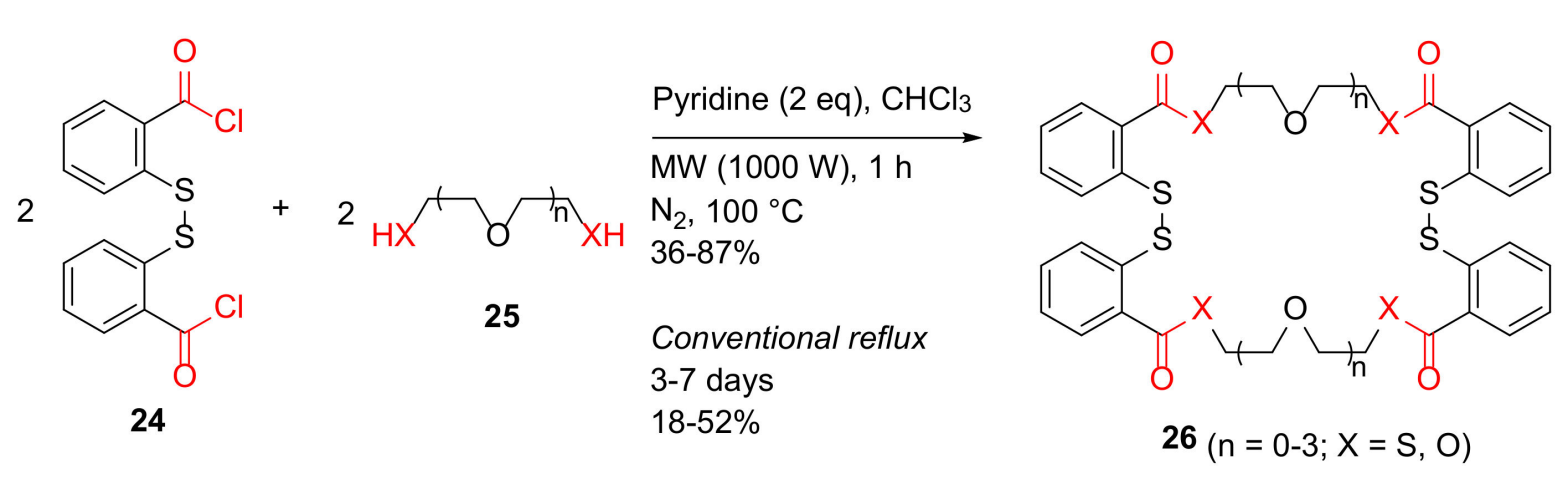
MW-assisted synthesis of benzo-thio crown ethers [43].

Calisir and Çiçek reported the MW-assisted synthesis of benzo-thio crown ethers and studies of their ion chelation properties in 2017 [43]. Benzo-thio crown ethers **26** were synthesized from 2,2′-dithiodibenzoyl chloride **24** and dithiol or diol **25** in pyridine–chloroform under MW irradiation at 100 °C for 1 h in 36–87% isolated yields, compared to 18–52% yields under conventional reflux for 3–7 days (Scheme 8) [43].

**Scheme 9:**
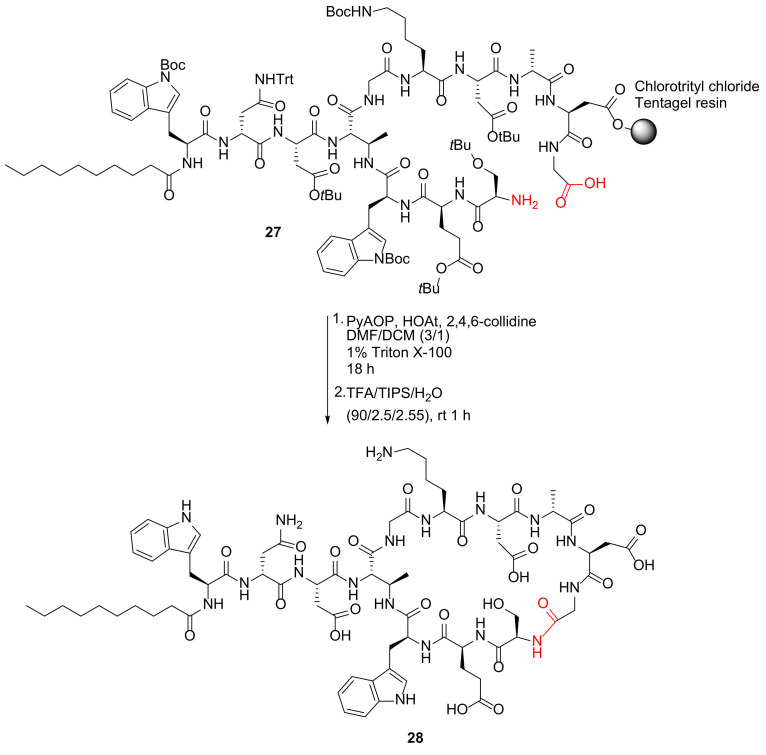
On-resin synthesis of macrocyclic daptomycin analog via the amide coupling strategy [44].

Moreira et al. reported the synthesis of macrocyclic daptomycin analogs via replacing the ester with an amide in 2019 [44]. A representative example is shown in Scheme 9. Specifically, macrocyclization of peptide **27** was performed using PyAOP/HOAt/2,4,6-collidine in DMF/DCM (3/1) containing 1% Triton™ X-100, followed by simultaneous resin cleavage and global deprotection to provide daptomycin analog **28** [44]. Previously, in 2016, Lohani et al. reported an alternative solid-supported synthesis of a daptomycin analog DapE12W13 only using single α-azido amino acid and on-resin macrocyclization was performed prior to the construction of the side chain containing the decanoyl tail [45]. In 2019, Itoh and Inoue reviewed solid-supported total synthesis of macrocyclic natural peptides with branched chains (e.g., polymyxin E2 and daptomycin) via four-dimensionally orthogonal protective group strategies [46].

**Scheme 10:**
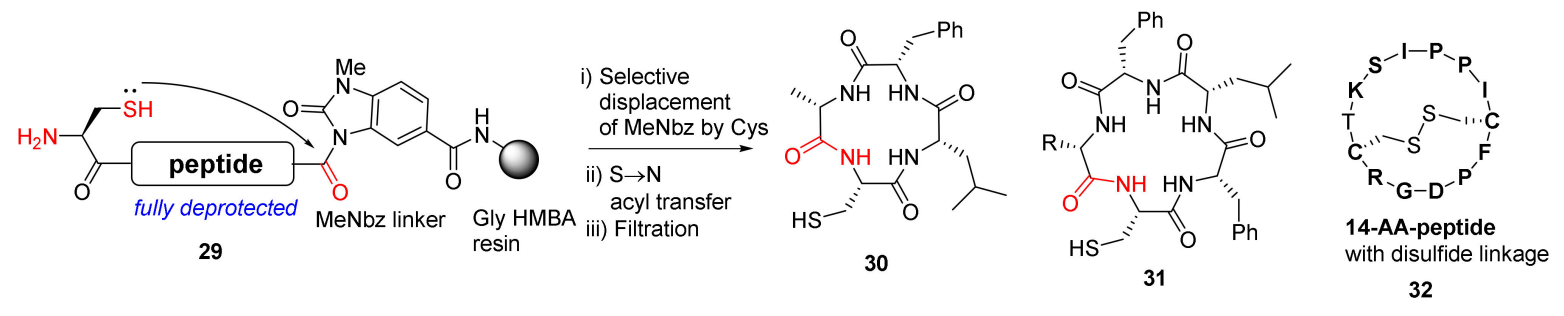
On-resin synthesis of cyclopeptides via selective displacement of MeNbz by Cys and acyl transfer strategy [47].

Arbour et al. reported the on-resin and self-cleaving head-to-tail macrolactamization of unprotected peptides via mild *N*-acyl urea activation in 2019 [47]. The macrocyclization of unprotected *N*-acyl urea-linked peptides **29** was mediated by *N*-terminal cysteine (Scheme 10). The S-to-N acyl transfer reaction, a robust and high-yielding chemoselective ligation methodology to form an amide bond, is widely used in organic synthesis, medicinal chemistry, and chemical biology [48]. Specifically, this efficient S-to-N acyl transfer cascade process involves the initial formation of a reactive thioester intermediate, followed by the acyl transfer to the nucleophilic NH_2_ group. In this work, diverse macrocycles such as tetra- and pentapeptides **30** and **31** and the intramolecular disulfide-linked 14-AA-peptides **32** (sunflower trypsin inhibitor 1) were synthesized and demonstrated, preventing head-to-tail dimer formation and hydrolysis of most substrates [47].

**Scheme 11:**
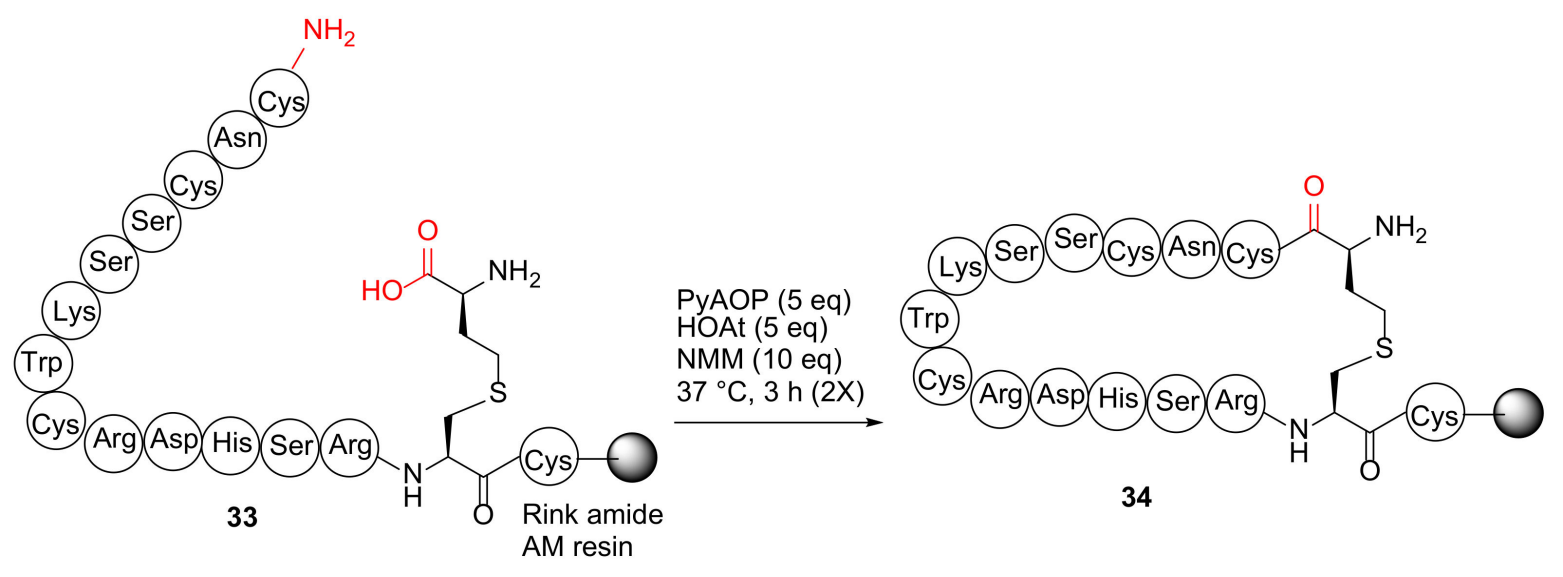
On-resin synthesis of cyclopeptide **34** [49].

Qu et al. reported the synthesis of disulfide surrogate peptides with large-span surrogate bridges via a native-chemical-ligation (NCL)-assisted diaminodiacid (DADA) approach in 2020 [49]. As an example, on-resin macrocyclization of linear peptide **33** was performed using PyAOP/HOAt/NMM at 37 °C for 3 h (2 times) to provide resin-bound 16-AA-peptide **34** with a thioether linkage as a complex mixture (Scheme 11) [49]. The failure of this on-resin DADA cyclization strategy was presumably due to its large 15AA ring size. Subsequently, a more effective NCL-assisted DADA strategy was developed to achieve large ring macrocyclization in the solution phase and with an alternative cyclization site (Trp8-Cys9) [49]. 

**Scheme 12:**
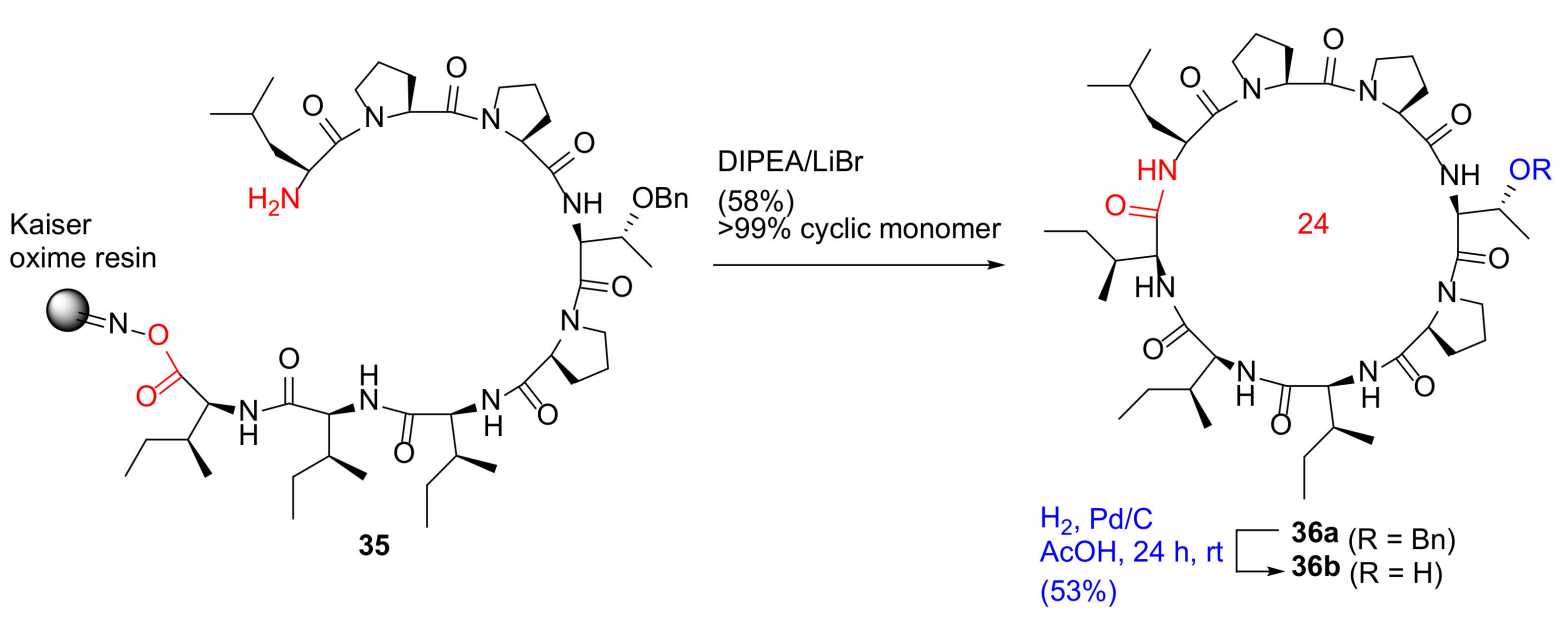
On-resin synthesis of dominicin via head-to-tail concomitant cyclization−cleavage strategy [50].

Bérubé et al. reported the solid-phase total synthesis and antimalarial evaluation of macrocyclic octapeptide dominicin, isolated from a marine sponge in 2020 [50]. The on-resin cyclization−cleavage reaction of **35** was performed using LiBr (5 eq) and DIPEA (2.5 eq) in CH_2_Cl_2_/THF (20:1), providing **36a** in a 58% yield as a cyclic monomer (>99%), based on the initial resin loading (Scheme 12). Subsequent catalytic Pd/C hydrogenation in acetic acid to remove the benzyl group on the side chain provided dominicin (**36b**) in a 53% yield after preparative HPLC purification. An alternative methodology was also developed using entirely on-resin synthesis and biorthogonal protection in *N*-Boc-L-Thr(O*t*Bu)-OH [50]. In 2018, Bérubé et al. reported the on-resin synthesis of pseudacyclins A–E via a similar head-to-side chain concomitant cyclization–cleavage strategy [51].

On-resin macrocyclizations of hexapeptides were previously reviewed by Prior et al. [52]; additional cyclopeptides include those synthesized via standard macrocyclization amide coupling strategy [53,54,55,56,57,58,59], synthetic peptide macrocycles with two-strand forming segments [60], on-resin macrolactamization [61], diaminodiacid-based macrocyclopeptides with a 1,2,3-triazole linker to mimic the disulfide bond [62], a cyclic peptide with a side chain-to-side chain cyclization and a central warhead residue [63], and solid-phase synthesis of radiolabeled somatostatin octapeptide analogs with macrocyclic disulfide linkage and conjugated to a tetraazacyclododecane chelator motif [64]. In 2013, Kumarn et al. reported the synthesis of cycloheptapeptide integerrimide A via an on-resin tandem Fmoc-deprotection–macrocyclization strategy [65]. Moreover, in 2013, Thakkar et al. analyzed >2 million peptides to investigate on-resin cyclization efficiency with regard to various ring sizes, peptide sequence, and solvent [66].

Other on-resin natural product peptide syntheses include solid-phase synthesis of the lantibiotic lactocin S [67], on-resin macrocyclization of tetrapeptide namalide [68], cyclotheonamide analogs as human β-tryptase inhibitors [69], cyclotheonamide E4 and analogs as potent and selective β-tryptase inhibitors [70], solid-phase synthesis of the proposed structure of cyclodepsipeptide coibamide A and its *O*-desmethyl derivative via on-resin macrocyclization [71], side chain to side chain cyclized opioid endomorphin-2 peptide analogs [72], on-resin synthesis and evaluation of the antibiotic lysocin E and analogs [73], on-resin synthesis of rapamycin analogs [74], parallel solid-phase synthesis and anti-inflammatory evaluation of cyclic peptides cyclosquamosin D and Met-cherimolacyclopeptide B and analogs [75], and synthesis and evaluation of backbone cyclic peptides as HIV-1 protein–protein interaction inhibitors [76]. In 2021, Yoshida et al. reported the solid-phase synthesis and bioactivity evaluation of cherimolacyclopeptide E [77]. Additional on-resin MW-assisted macrocyclization of peptides via amide coupling was reported by Rashad et al. in 2015 [78] and Aneja et al. in 2019 [79]. 

### 2.2. Transition-Metal Catalyzed Olefin Ring-Closure Metathesis (RCM) Macrocyclization

**Scheme 13:**
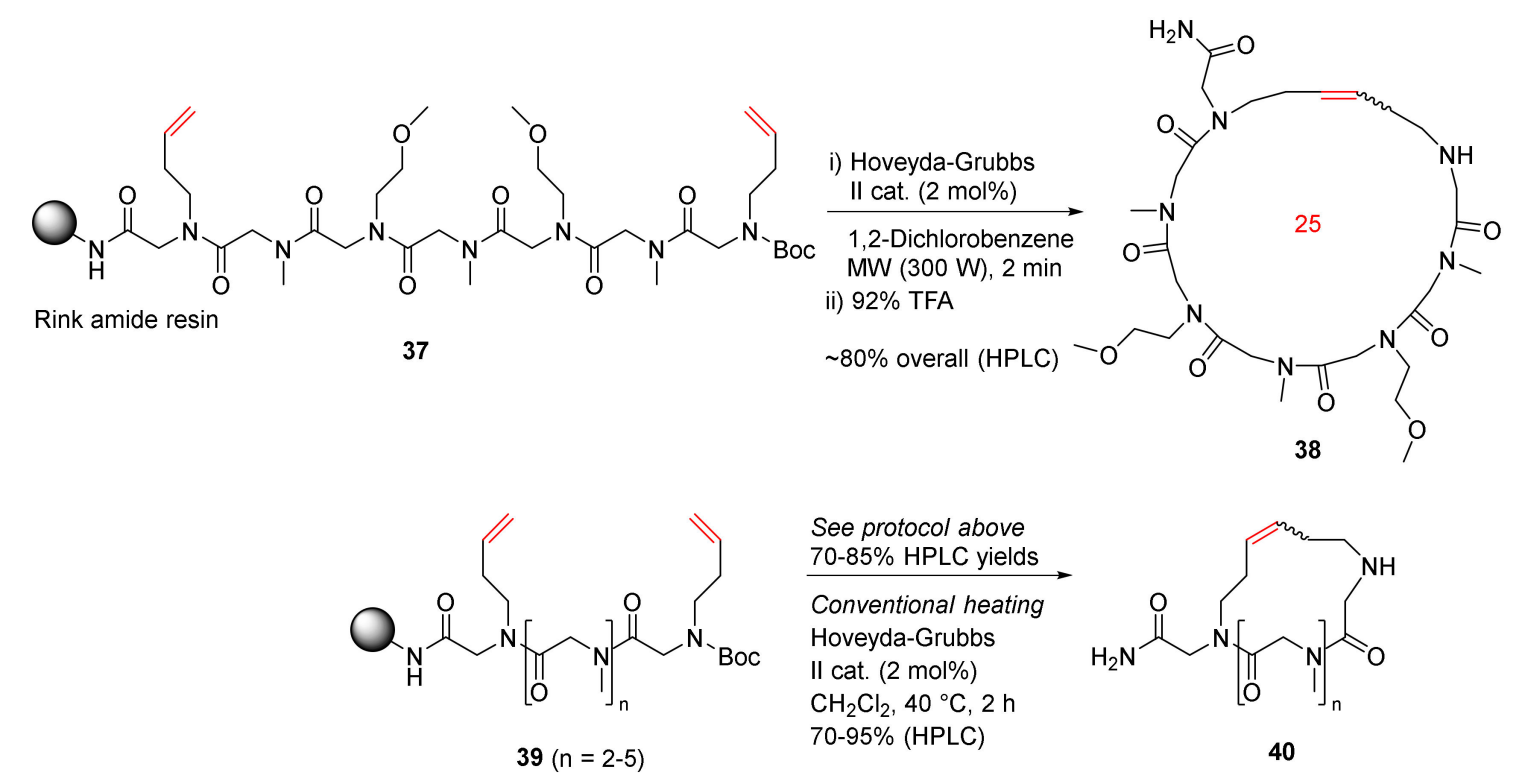
Solid supported MW-assisted RCM of peptoids [80].

Khan et al. reported the MW-assisted solid-phase synthesis of cyclic peptoids via the RCM strategy in 2011 [80]. Synthesis of **38** was performed from the model peptoid **37**, a heptamer with 3-buten-1-amine at the first and last positions, using Hoveyda–Grubbs second-generation catalyst (2 mol%) in 1,2-dichlorobenzene under MW irradiation for 2 min (4 × 30 s) (Scheme 13). The HPLC yield of the cyclic peptoid **38** as a mixture of *E*/*Z* isomers was ~80% following the resin cleavage in 92% TFA [80]. Accordingly, cyclopeptoids **40** with different ring sizes were synthesized from **39** in 70–85% HPLC yields using the same MW RCM protocol. In contrast, the HPLC yields were slightly higher when the reactions were performed under conventional heating in dichloromethane at 40 °C for 2 h, but with longer reaction times [80].

**Scheme 14:**
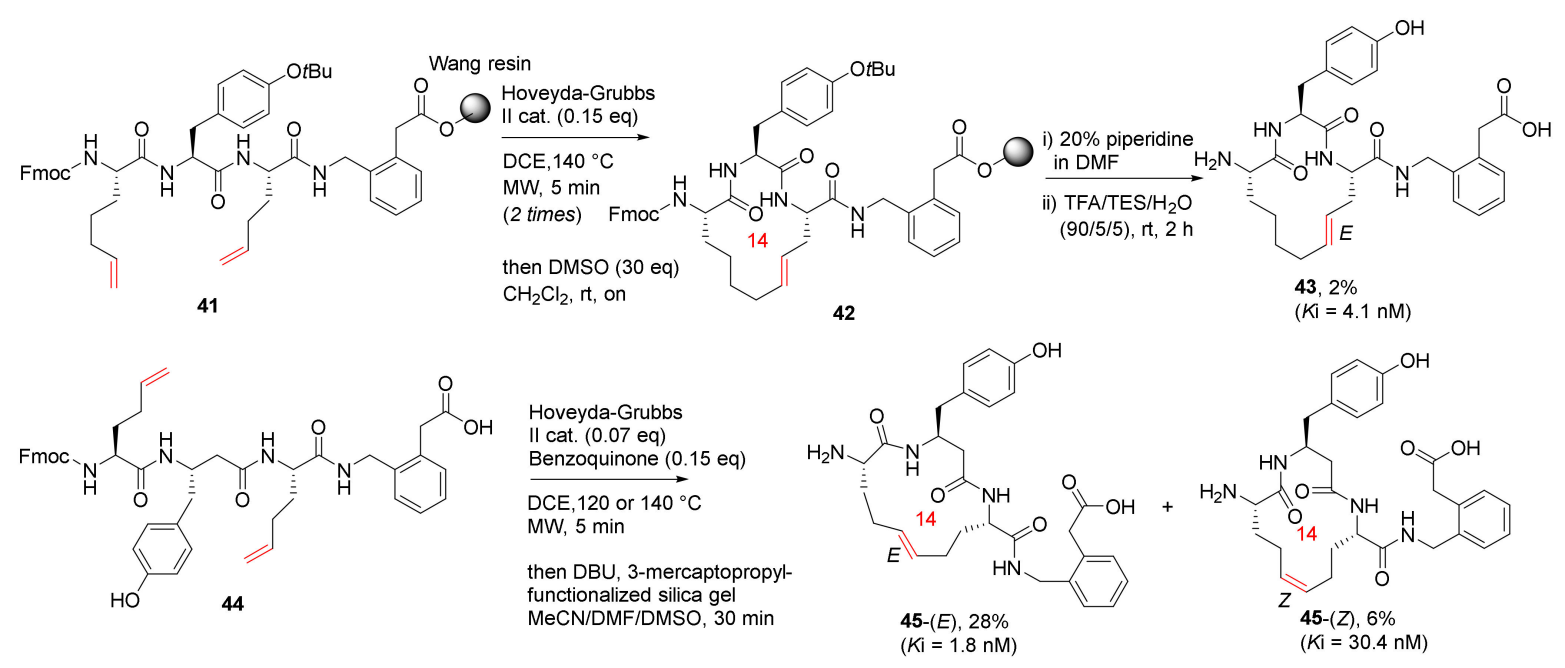
On-resin or solution phase MW-assisted RCM cyclization of 14-membered **43** and **45** [81].

Andersson et al. reported the synthesis and biological evaluation of low nanomolar macrocyclic inhibitors of insulin-regulated aminopeptidase (IRAP) via MW-assisted solid or solution-phase olefin RCM in 2011 [81]. Macrocycles **43**-(*E*) and **45**-(*E*), the most potent IRAP inhibitors with high stability against proteolysis by metallopeptidases, were synthesized from **41** and **44** on solid and solution phase, respectively (Scheme 14). The Wang resin was used in the MW-assisted solid-phase synthesis of **41**, and the on-resin RCM was performed at 140 °C using the Hoveyda–Grubbs second-generation catalyst and 1,2-dichloroethane (DCE) as solvent under microwave heating [81]. Interestingly, **43** was isolated from a complex reaction mixture and obtained in 2% yield, following isomerization of **41**, ring contraction, and subsequent double bond migration [81]. Accordingly, macrocycles **45**-(*E*) and **45**-(*Z*) were obtained in 28% and 6% yields, respectively. 

**Scheme 15:**
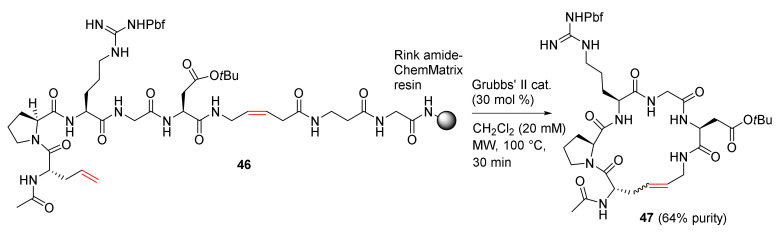
MW-assisted RCM cyclization–cleavage of pseudopeptide [82].

Baron et al. reported the MW-assisted synthesis of macrocyclic pseudopeptides via RCM cyclization–cleavage strategy using a *cis*-5-aminopent-3-enoic acid (*cis*-Apa) linker in 2011 [82]. Macrocycle **47** with an RGD motif was synthesized from **46** using Grubbs’ second-generation catalyst in dichloromethane at 100 °C under MW irradiation in 64% purity (Scheme 15). In contrast, when this reaction was performed at 60 °C for 5 h under MW irradiation without the catalyst, **47** was obtained in a higher 73% purity. Following reverse-phase chromatography purification, **47** was isolated as two configurational *E* and *Z*-isomers (3/1 ratio) in an overall 25% yield from the resin and a 58% cyclization yield from **46**. In addition, compared to conventional heating, MW heating is more efficient with higher yields and shorter reaction times [82]. Macrocyclic RGD peptides may have the potential to function as potent and/or selective α_5_β_1_ and α_ν_β_3_ integrin antagonists [83].

**Scheme 16:**
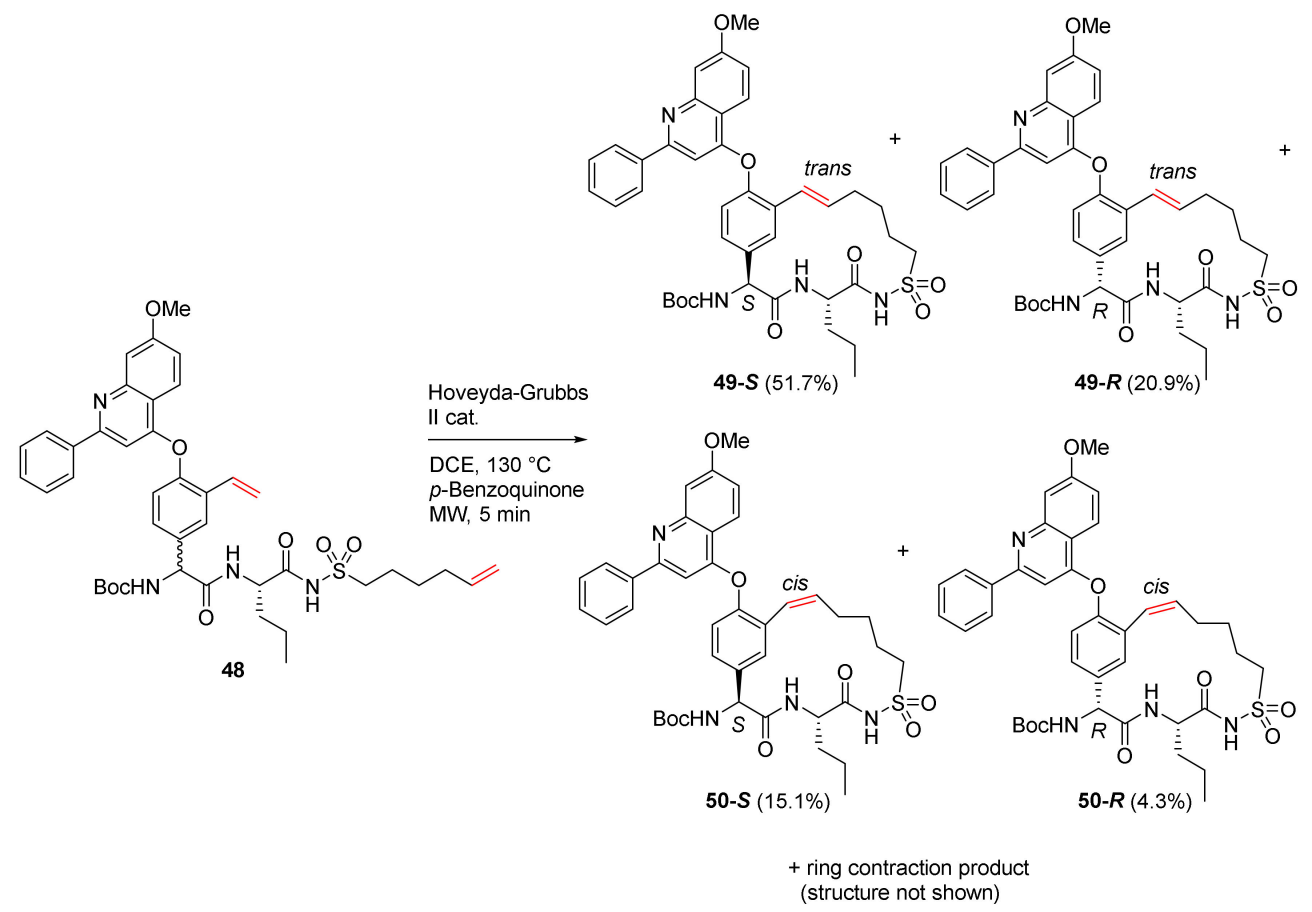
MW-assisted 16-membered RCM products of **48** [84].

Lampa et al. reported the RCM macrocyclization of the P2 phenylglycine and the alkenylic P1′ and HCV NS3 protease inhibition in 2011 [84]. As an example, the RCM of diastereomeric starting material **48** using Hoveyda–Grubbs’ second-generation catalyst in dichloroethane at 130 °C under MW irradiation is shown in Scheme 16. *p*-Benzoquinone was also added in the reaction to minimize the formations of ring-contracted and double bond migration side products. Four RCM products, **49-*S***, **49-*R***, **50-*S***, and **50-*R***, were obtained as stereoisomers R/S and configurational *E* and *Z*-isomers. In order to evaluate the reaction outcome, the impact of different methods (e.g., increasing temperature and MW irradiation) was measured, and it was found that the substrate appeared to play a more important role in affecting reaction outcomes relative to the cyclization method [84].

**Scheme 17:**
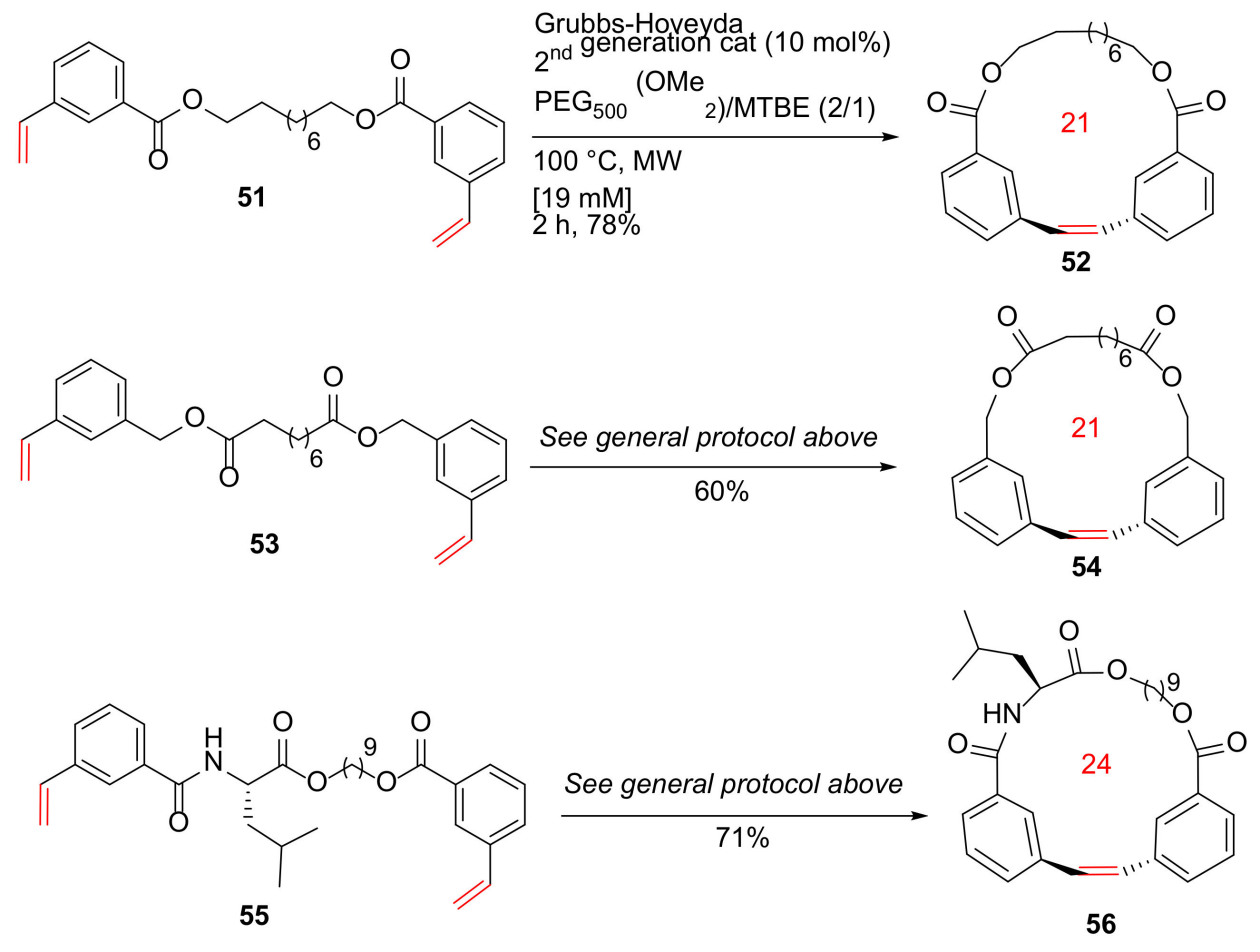
MW-assisted RCM macrocyclizations at high concentrations [85].

Raymond et al. reported the MW-assisted macrocyclic olefin metathesis at high concentrations using a phase-separation strategy in 2014 [85]. In this work, a protocol to promote macrocyclic olefin metathesis was developed at relatively high concentrations (up to 60 mM) under MW irradiation. As examples, diverse macrocyclic scaffolds such as **52**, **54**, and **56** with different alkyl, aryl, or amino acid spacers were synthesized from their respective diene substrates in the presence of the Grubbs–Hoveyda second-generation catalyst and a mixture of PEG_500_(OMe_2_)/methyl *t*-butyl ether (MTBE) at 100 °C under MW heating for 2 h in 60–78% yields (Scheme 17).

**Scheme 18:**
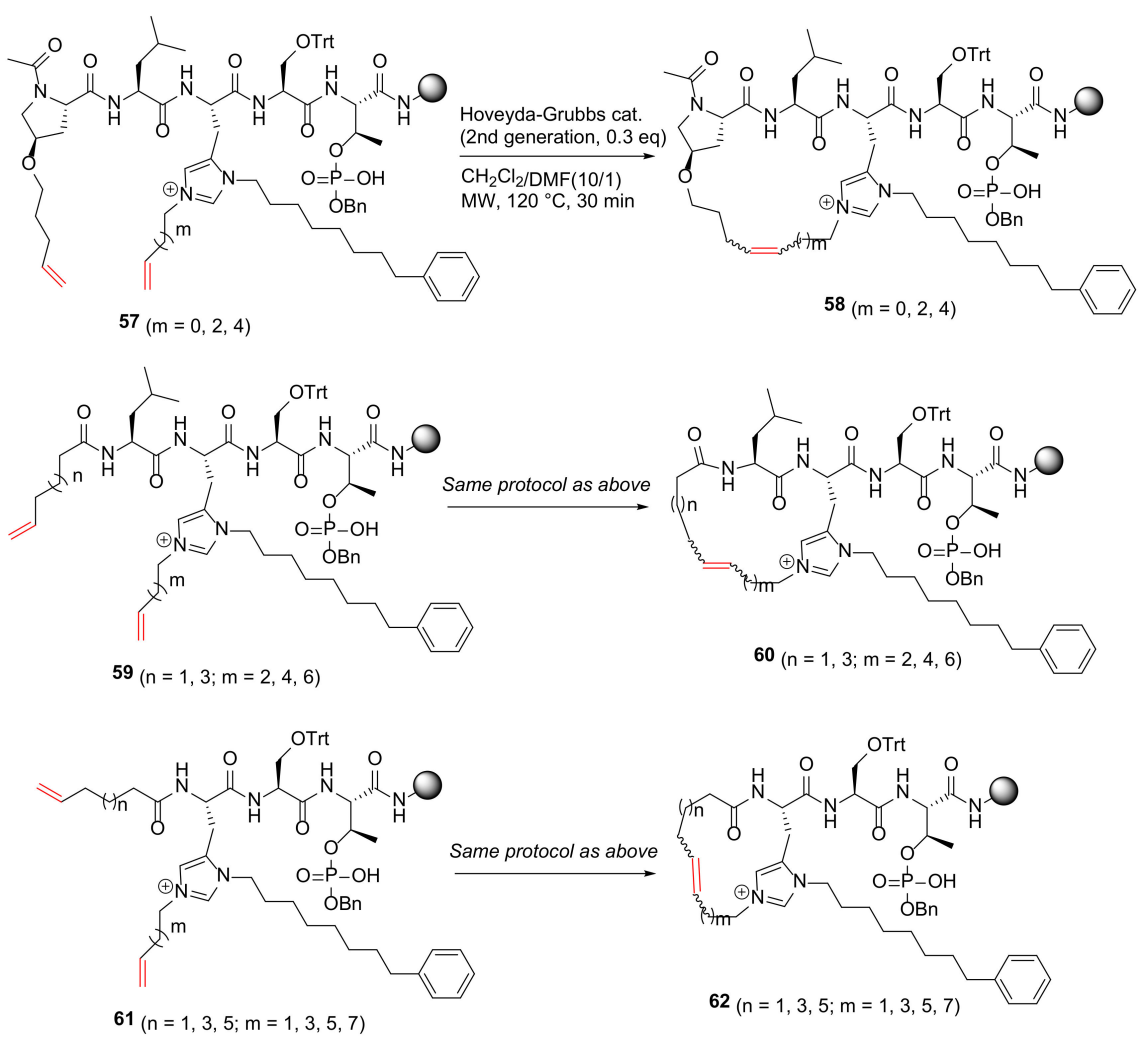
Solid-supported MW-assisted RCM synthesis of charge-masked macrocyclic phosphopeptides [86].

Qian et al. reported the synthesis of imidazolium-containing phosphopeptide macrocycles via neighbor-directed histidine N(τ)-alkylation in 2015 [86]. MW-assisted RCM reaction of **57** was performed on a solid support and by leveraging the Pro residue with a pentenyloxy side chain and the adjacent N(τ)–alkenyl group to produce various 20-, 22-, or 24-membered macrocycles **58**, using second-generation Hoveyda–Grubbs catalyst in CH_2_Cl_2_/DMF (10/1) under microwave irradiation at 120 °C for 30 min (Scheme 18). For this reaction, a solvent combination of DMF and CH_2_Cl_2_ is important for desirable peptide solubility, MW absorption, and resin swelling [86]. Syntheses of charge-masked macrocyclic phosphopeptides **58**, **60**, and **62** and subsequent derivatives bearing the bis-alkyl-His imidazolium ring have the application potential toward biologically important protein-protein interactions.

**Scheme 19:**
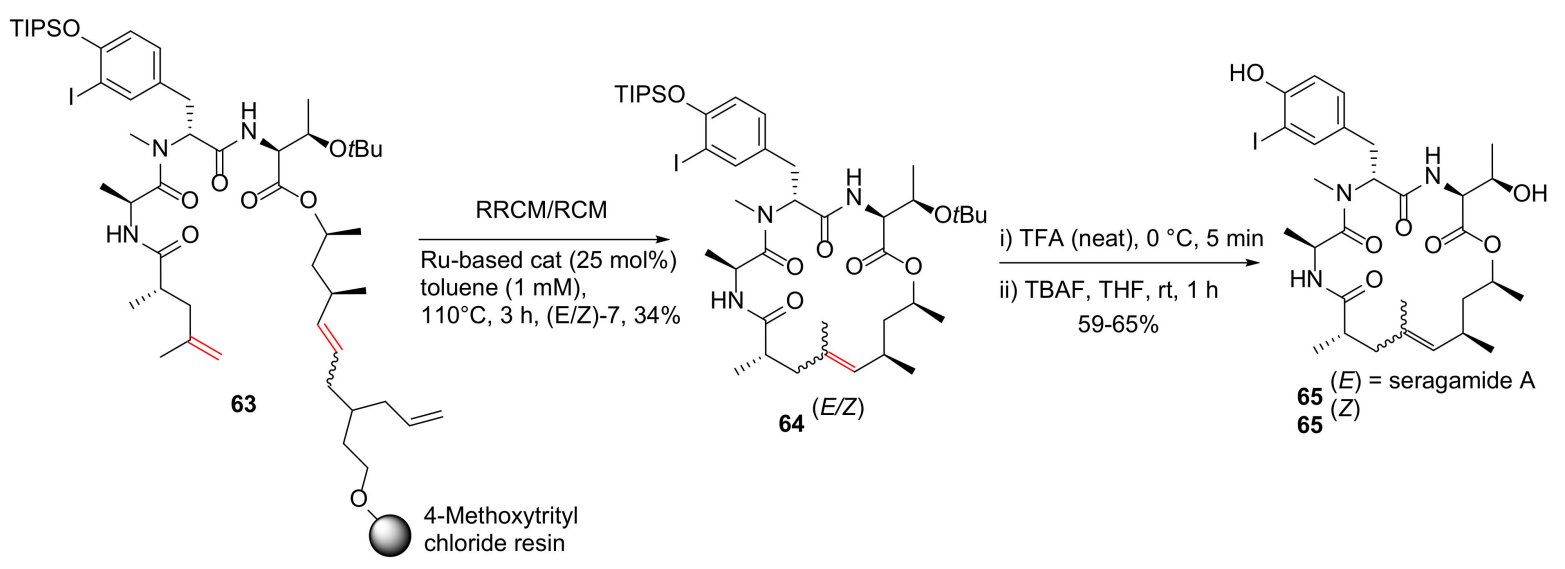
Synthesis of a representative azole-enriched cyclopeptide **13** via an on-resin cyclization–cleavage strategy [87].

Arndt et al. reported a divergent on-resin synthesis of macrocyclic depsipeptide seragamide A via relay-ring-closing metathesis (RRCM) strategy in 2015 [87]. Specifically, an on-resin RRCM reaction of **63** was performed using a Ru-based catalyst (25 mol%) in refluxing toluene at 110 °C for 3 h to provide the protected natural product **64** in 34% yield. Following sequential *O*-*t*Bu and TIPS-O deprotection of individual **64** (*E*/*Z*) isomers in TFA and TBAF, seragamide A **65** (*E*), and its Z-olefin isomer was obtained in 65 and 59% yields, respectively (Scheme 19) [87].

**Scheme 20:**
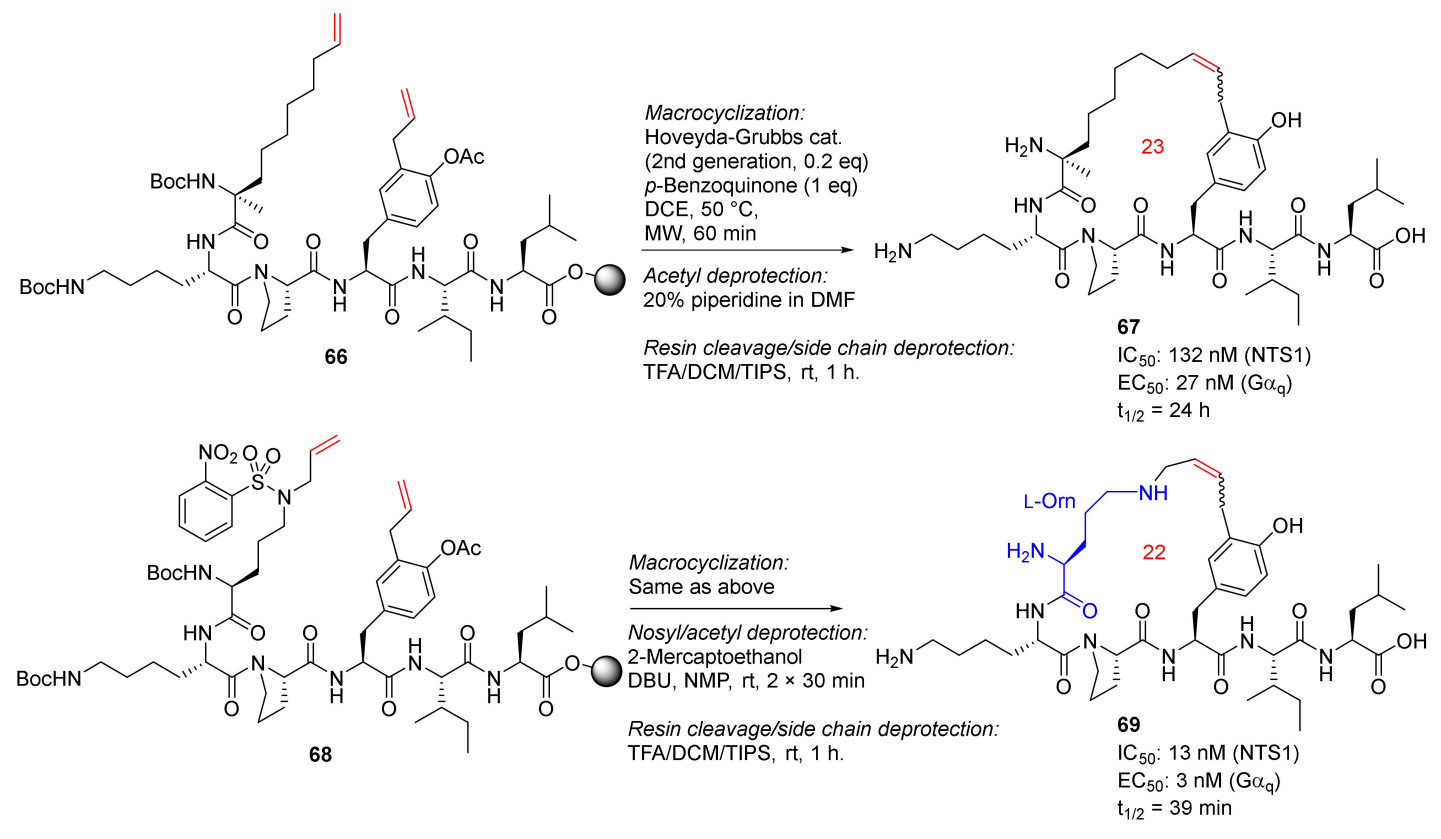
Solid-supported MW-assisted RCM reactions to produce 23- and 22-membered macrocycles **67** and **69 [88]**.

Sousbie et al. reported the discovery, synthesis, and chemistry optimization of macrocyclic neurotensin analogs with potent analgesic effects in 2018 [88,89]. RCM macrocyclizations of **66** and **68** were performed under microwave heating at 50 °C using Hoveyda−Grubbs second-generation catalyst and *p*-benzoquinone in DCE for 1 h, followed by the acetyl deprotection for **66** using 20% piperidine in DMF and simultaneous deprotection of the nosyl and acetyl groups for **68** using 2-mercaptoethanol and DBU, and final resin cleavage and side-chain deprotection in TFA/DCM/TIPS (Scheme 20). Two promising macrocycle candidates, **67** and **69**, with low nanomolar potency and/or good plasma stability, showed in vivo efficacy in two rodent pain models [88].

**Scheme 21:**
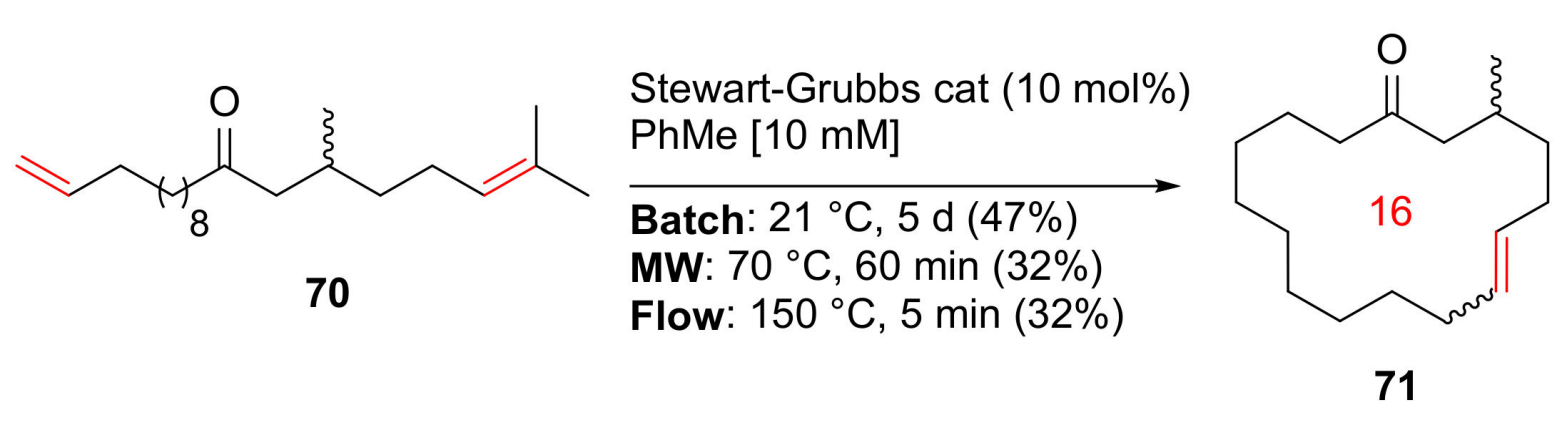
The RCM macrocyclization of musk **71** via batch, MW, and continuous flow strategies [90].

Morin et al. reported the synthesis and evaluation of a renewable macrocyclic musk using batch, MW, and continuous flow methodologies in 2019 [90]. At 10 mM concentration of the diene **70** in toluene, macrocycle **71** was synthesized in 32% yield under MW irradiation in the presence of Stewart–Grubbs catalyst at 70 °C for 60 min (Scheme 21). In contrast, batch synthesis at 21 °C for 5 days and continuous flow strategy at 150 °C for 5 min gave 47% and 32% yields, respectively, offering the advantage of facile scale-up (>1 g) in both cases [90].

**Scheme 22:**
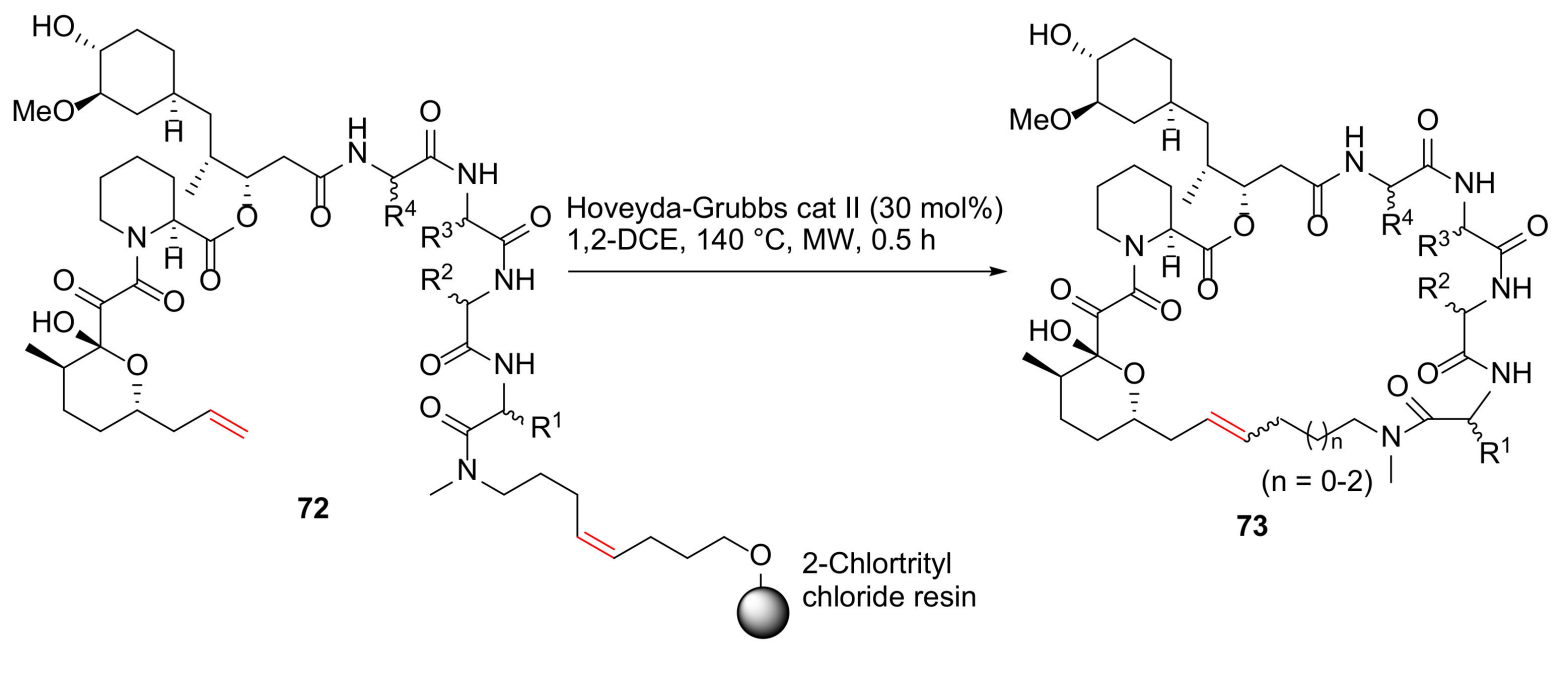
Solid-phase synthesis of rapafucin via on-resin RCM/cyclative release strategy [91].

Guo et al. reported the synthesis and evaluation of rapamycin-inspired macrocycles with new target specificity in 2019 [91]. A general synthetic route to rapafucin is shown in Scheme 22. Specifically, on-resin MW-assisted RCM cyclization–cleavage was performed from **72** using Hoveyda–Grubbs catalyst II (30 mol%) in 1,2-dichloroethane at 140 °C for 0.5 h to provide macrocycles **73** [91].

**Scheme 23:**
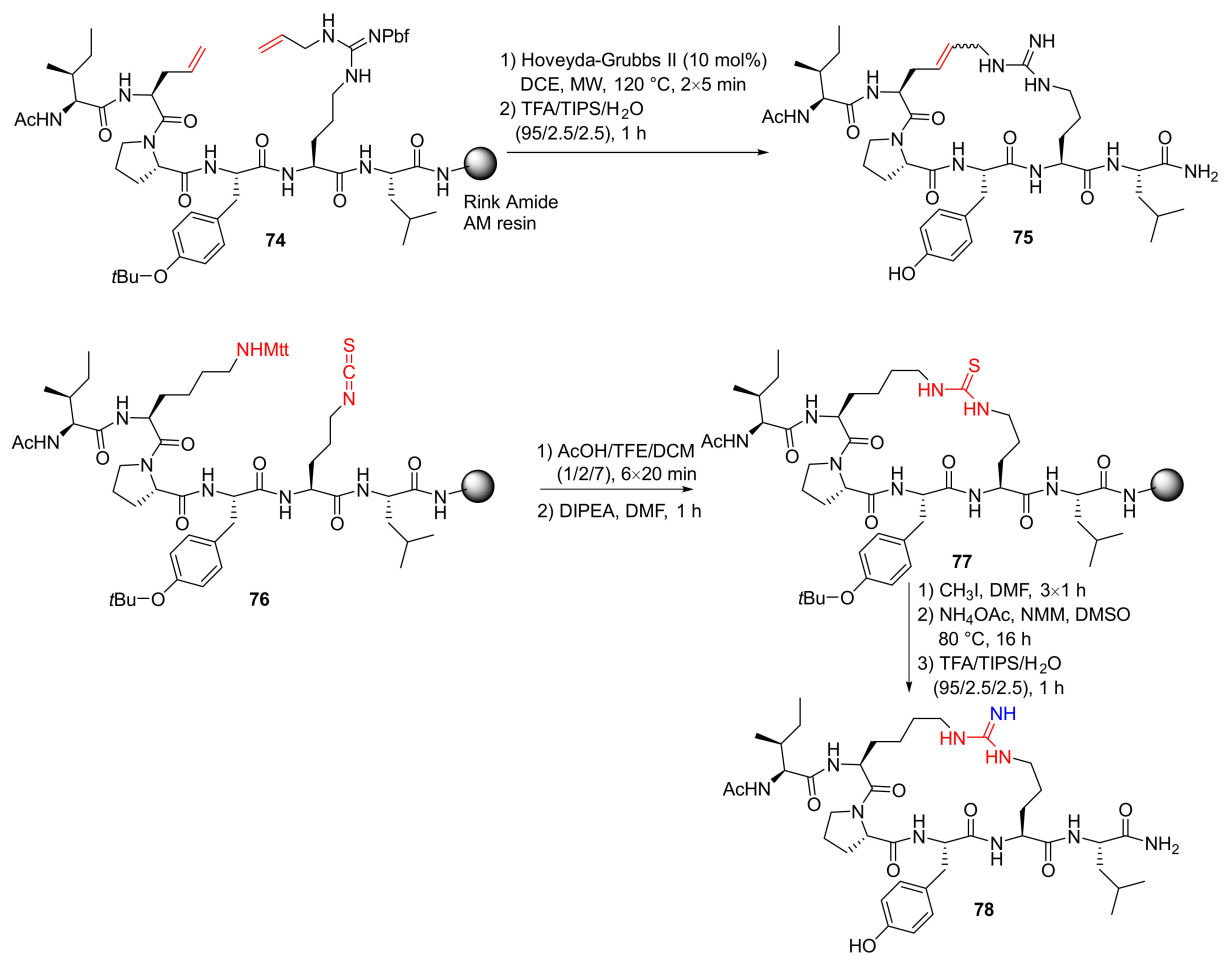
On-resin synthesis of macrocyclic peptide analogs via the RCM and thiourea/guanidine strategy [92].

‘T Hart et al. reported the hot-spot guided design and synthesis of macrocyclic peptide inhibitors of the LSD1-CoREST1 interaction in 2019 [92]. Specifically, RCM macrocyclization of **74** was performed using the Hoveyda–Grubbs II catalyst and on-resin MW irradiation to provide **75**, following final resin cleavage and deprotection by TFA. In addition, following the removal of the Mtt-group of **76** in mildly acidic conditions, on-resin cyclization of the deprotected peptide was performed upon treatment with DIPEA to provide solid-supported **77**, followed by the conversion of thiourea to the desired guanidine and subsequent resin cleavage and side-chain deprotection to yield **78** (Scheme 23) [92].

**Scheme 24:**
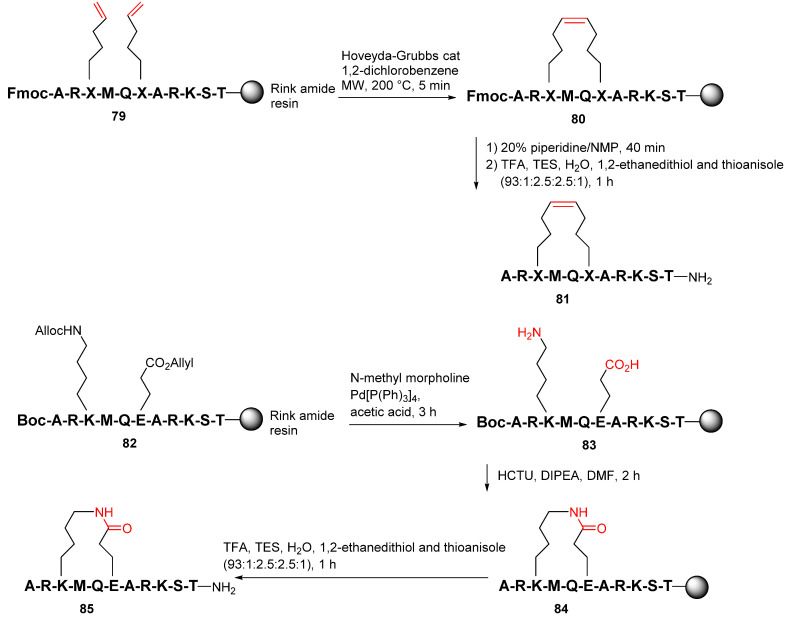
On-resin synthesis of macrocyclic peptides via RCM or lactam strategy [93].

Yang et al. reported the synthesis and evaluation of several series of macrocyclic peptides as reversible inhibitors of LSD1 with a novel binding mode in 2020 [93]. On-resin RCM was performed from the *N*-terminal Fmoc and the side-chain protected **79** using the Hoveyda−Grubbs catalyst under MW irradiation at 200 °C to provide **80** (Scheme 24). Following the Fmoc and side-chain deprotection and resin cleavage, target peptide **81** was obtained. Accordingly, after the removal of allyl and allyloxycarbonyl protecting groups of **82** using Pd(PPh_3_)_4_, on-resin lactamization of **83** was then performed using the HCTU coupling reagent, followed by the global deprotection and simultaneous resin cleavage with TFA to provide the lactamized peptide **82** [93].

**Scheme 25:**
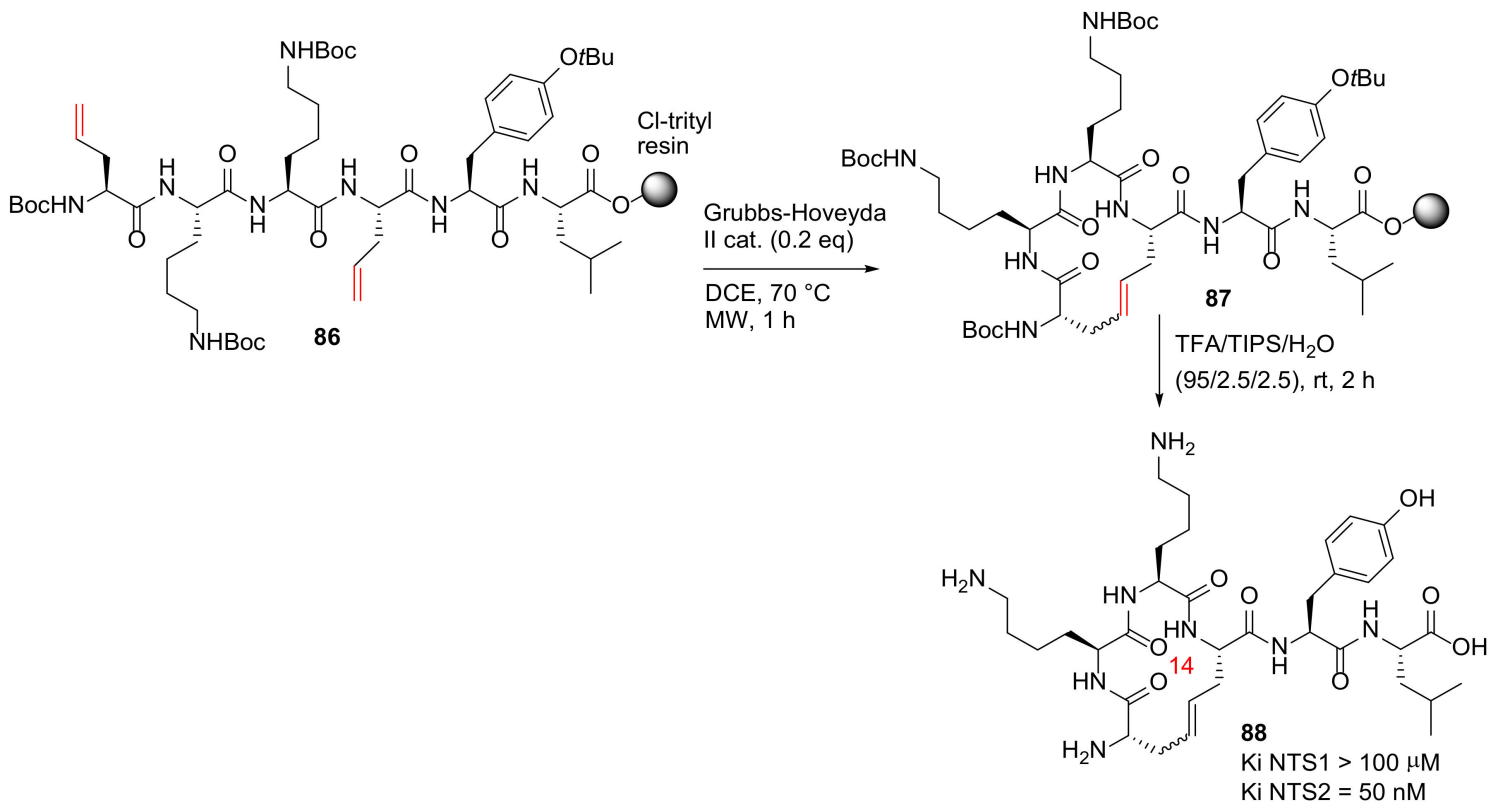
MW-assisted on-resin RCM cyclization of 14-membered lead candidate **88 [94]**.

Chartier et al. reported the design, synthesis, and evaluation of the first selective macrocyclic neurotensin (NT) receptor type 2 non-opioid analgesic in 2021 [94]. Macrocycle **88**, the most potent IRAP inhibitor with high stability against proteolysis by metallopeptidases, was synthesized from **86** on solid-phase (Scheme 25). The 2-chlorotrityl chloride resin was used in the MW-assisted solid-phase synthesis of **86**, and the on-resin RCM was performed between two allylglycine residues at 70 °C using the second generation Grubbs–Hoveyda catalyst and DCE as solvent under microwave heating in ~70% yield, judged by UPLC-MS [94]. Based on this developed methodology, a series of new NT macrocyclic analogs with various side chain to side chain cyclizations and therapeutic potential were synthesized to probe the chemical space associated with NT receptor binding, as well as the in vivo efficacy study in rodent pain models [94].

**Scheme 26:**
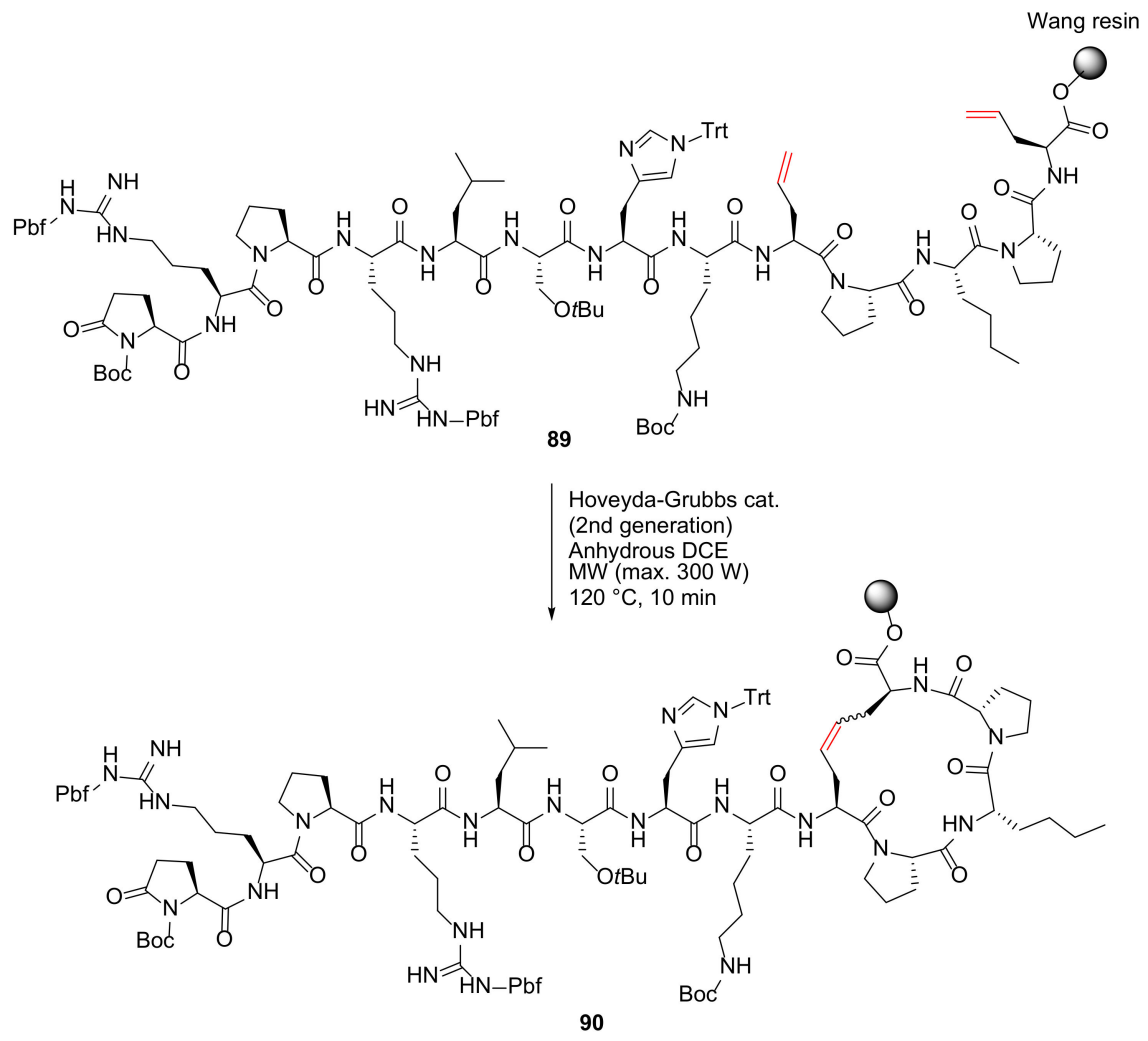
Solid-supported MW-assisted RCM synthesis of a 17-membered cyclopeptide analog [95].

Trân et al. reported the synthesis and evaluation of a series of apelin-13 based macrocyclic peptide analogs as modulators and pharmacological tools in the cardiovascular system in 2018 [95]. As an example, MW-assisted RCM reaction of **89** was performed on a solid support to produce a 17-membered macrocycle **90** using second-generation Hoveyda–Grubbs catalyst in DCE under microwave irradiation at 120 °C for 10 min (Scheme 26) [95]. Notably, the cleaved and side-chain deprotected compound shows a comparable binding affinity and a longer half-life in vivo relative to its linear peptide analog [95].

Other on-resin RCM reactions include the synthesis of a linked amino acid mimetic macrocycle under MW heating by Maxwell et al. in 2013 [96], diverse macrocyclization strategies using on-resin RCM, lactamization and thiol alkylation [97], synthesis of novel echinocandin analogs via on-resin RCM or disulfide formation strategy [98], the synthesis of peptides with olefin crosslinks [99], synthesis of peptide thioureas and thiazole-containing macrocycles via Ru-catalyzed RCM [100], and Grb2 SH3 domain-binding peptides [101].

### 2.3. Intramolecular C–C, and C–Heteroatom Coupling Reactions

**Scheme 27:**
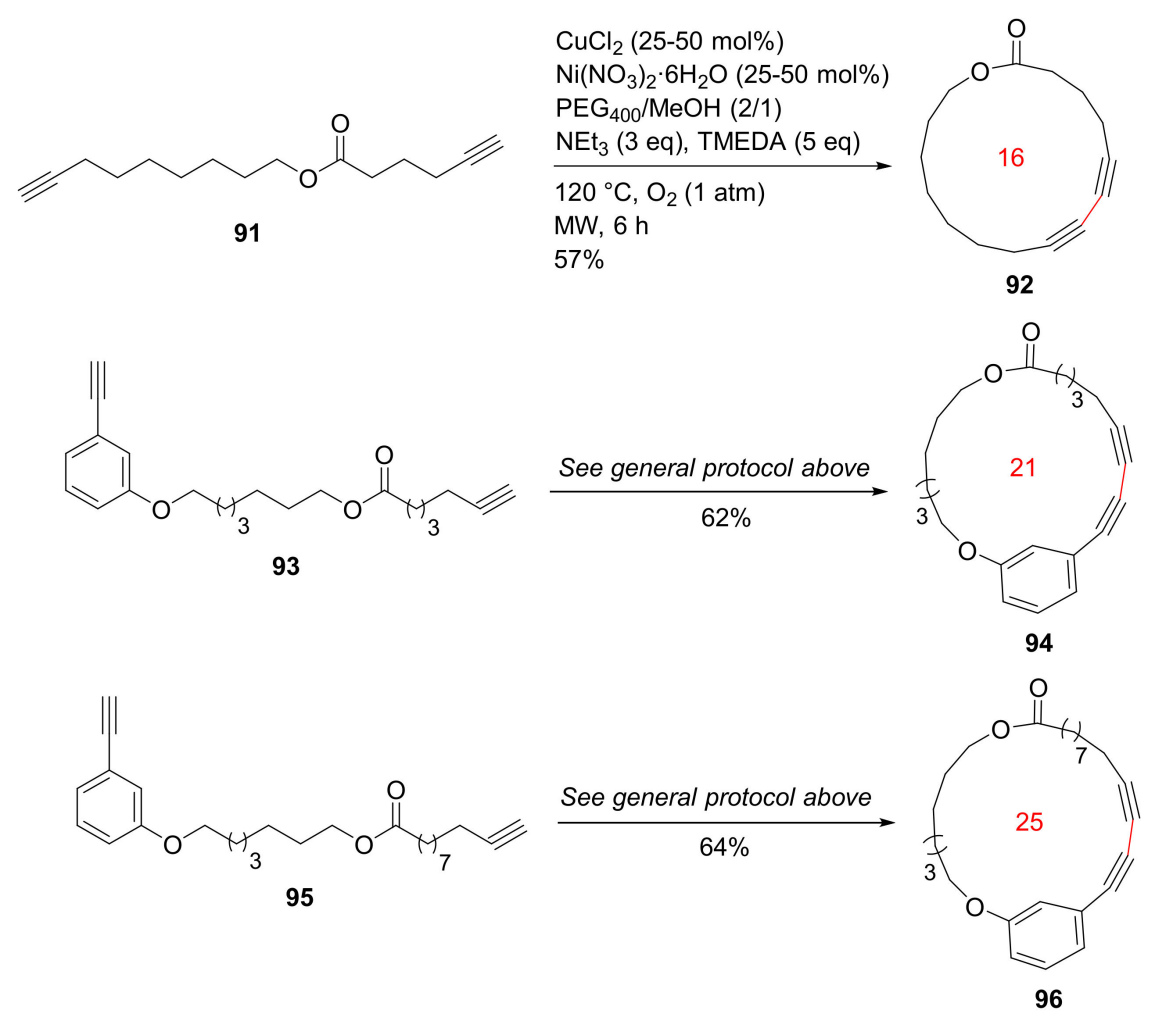
MW-assisted Glaser–Hay macrocyclizations at high concentrations [102].

Bédard and Collins reported the MW-accelerated macrocyclizations of diynes via Glaser–Hay oxidative coupling reactions in 2012 [102]. Efficient macrocyclizations of diynes **91**, **93**, and **95** were performed at high concentrations employing MW irradiation and a phase-separation strategy. The Glaser–Hay macrocyclizations were completed in 1–6 h under microwave heating to provide macrolactones **92**, **94**, and **96** (Scheme 27), in contrast to conventional heating for 2 days. In addition, macrocyclization concentrations under MW heating could be increased up to 100 mM, relative to traditional concentration levels (0.2 mM) [102].

**Scheme 28:**
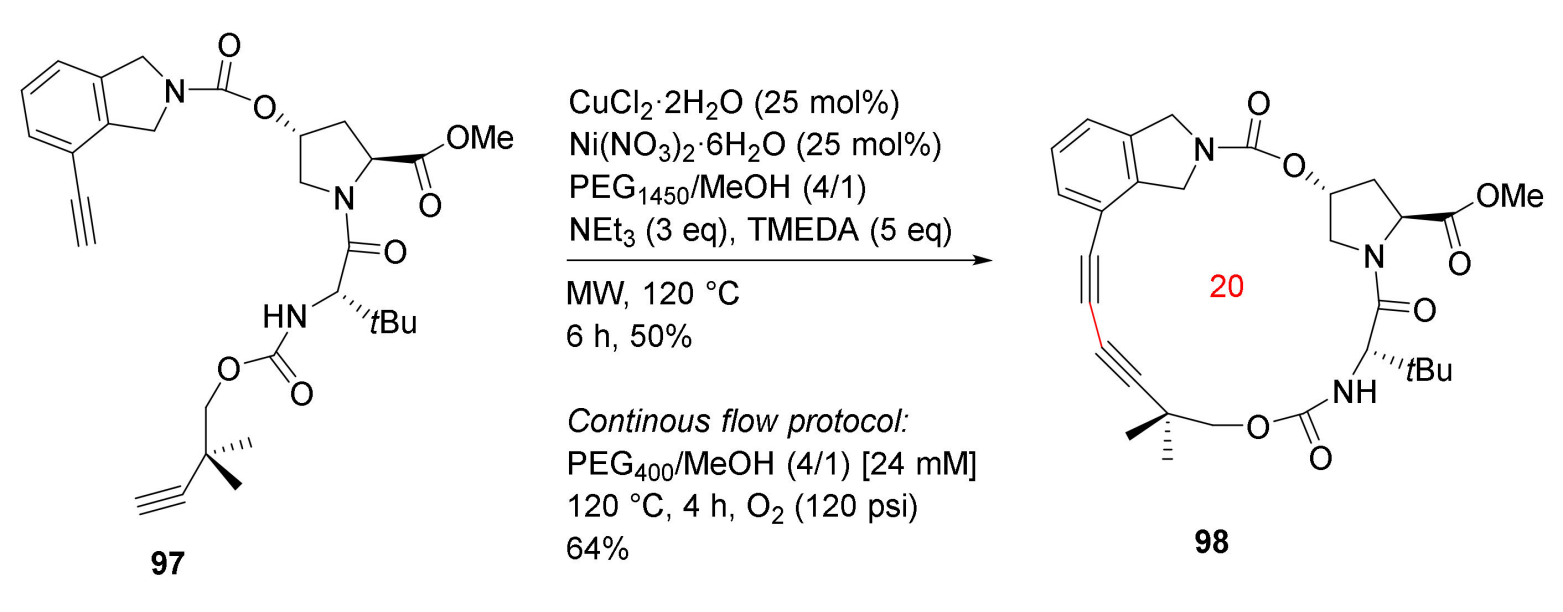
Glaser–Hay coupling macrocyclizations [103].

Godin et al. reported the MW-accelerated Glaser–Hay macrocyclizations and application toward the synthesis of antiviral agent vaniprevir in 2017 [103]. Glaser–Hay macrocyclization of **97** was performed employing MW irradiation and a phase separation strategy to provide **98**, a macrocyclic core of the hepatitis C virus NS3/5A protease inhibitor vaniprevir. The Glaser–Hay cyclization was completed in 6 h under MW heating in 50% yield (Scheme 28). In contrast, the phase separation/continuous flow protocol provided **98** in 4 h in a 64% yield [103].

**Scheme 29:**
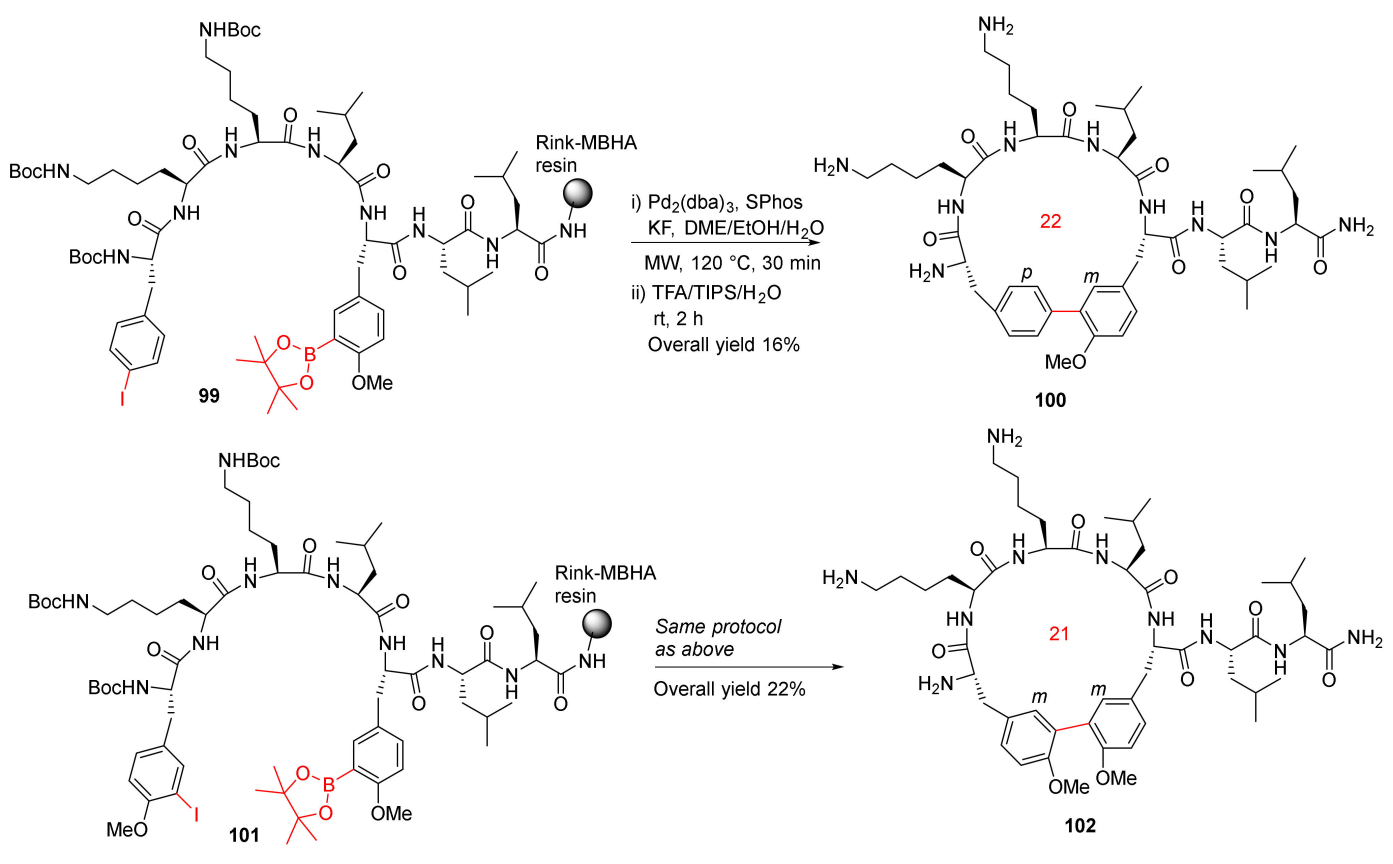
MW-assisted synthesis of biaryl cyclopeptides via Suzuki–Miyaura cross-coupling [105].

In order to further extend their previous work on MW-assisted synthesis of biaryl cyclopeptides via solid-supported intramolecular Suzuki–Miyaura coupling [104], in 2012, Afonso et al. reported the MW-assisted solid-phase synthesis of biaryl cyclic peptides containing a 3-aryltyrosine motif [105]. On-resin Suzuki–Miyaura macrocyclizations of **99** and **101** were performed using Pd_2_(dba)_3_ (0.2 eq), SPhos (0.4 eq), and KF (4 eq) in DME/EtOH/H_2_O (9/9/2) under MW irradiation at 120 °C for 30 min, followed by simultaneous resin cleavage and side-chain deprotection in TFA/TIPS/H_2_O to provide **100** and **102** in 16% and 22% overall yields, respectively (Scheme 29). This methodology demonstrates the synthesis of biaryl cyclopeptides with a *p*-Phe-*m*-Tyr or *m*-Tyr-*m*-Tyr linkage via solid-phase intramolecular Suzuki–Miyaura cross-coupling [105]. In the same year, Meyer et al. reported the solid-phase synthesis of 14- to 21-membered m,m-bridged biaryl macrocyclic peptides via on-resin Suzuki−Miyaura cross-coupling [106].

**Scheme 30:**
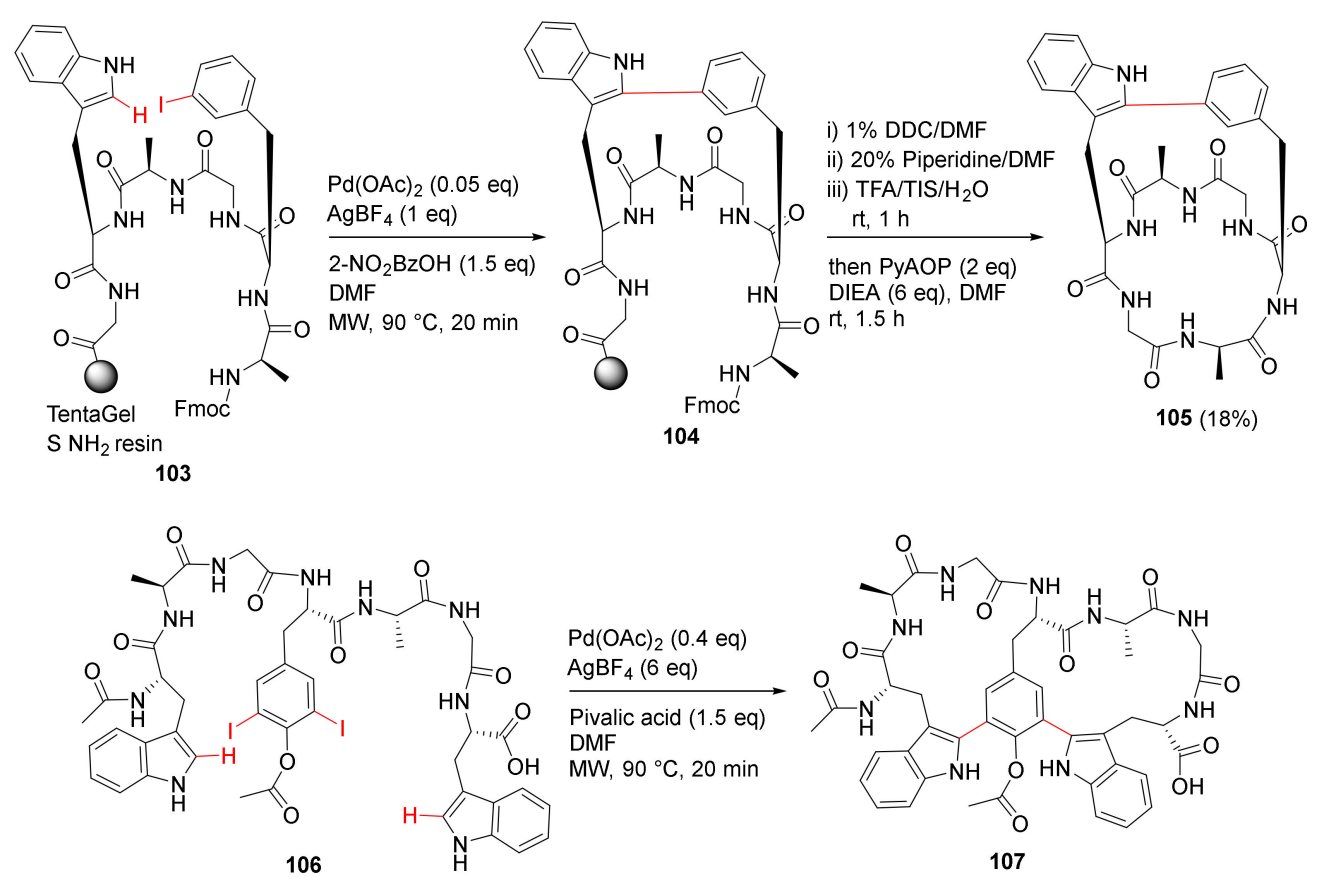
MW-assisted macrocyclization of the stapled peptides [107].

Mendive-Tapia et al. reported new peptide architectures and the MW-assisted macrocyclization between Trp–Phe/Tyr residues via a selective Pd-catalyzed C–H arylation strategy in 2015 [107]. As examples, solid-supported macrocyclization of **103** was performed using Pd(OAc)_2_ (0.05 eq), 2-nitrobenzoic acid (1.5 eq), and AgBF_4_ (1 eq) in DMF under MW irradiation (250 W) at 90 °C for 20 min, followed by the deFmoc, resin cleavage, and solution phase cyclization to provide bicyclopeptide **105** in an 18% unoptimized yield (Scheme 30) [107]. Accordingly, one-step double arylation to biaryl–biaryl stapled peptide **107** was synthesized from the linear peptide **106**, AgBF_4_ (6 eq), pivalic acid (1.5 eq), and Pd(OAc)_2_ (0.4 eq) under MW irradiation (250 W) at 90 °C for 20 min, provided the bicyclopeptide **107** [107]. This developed one-step simple protocol enabled access to novel constrained cyclopeptides (e.g., dimeric macrocycles, stapled bicyclopeptides, and biaryl–biaryl analogs) from their corresponding linear precursors, facilitating further application and property studies in chemical biology and medicinal chemistry. In 2012, Dong et al. reported the peptidic macrocyclization via Pd-catalyzed chemoselective indole C-2 arylation strategy [108].

**Scheme 31:**
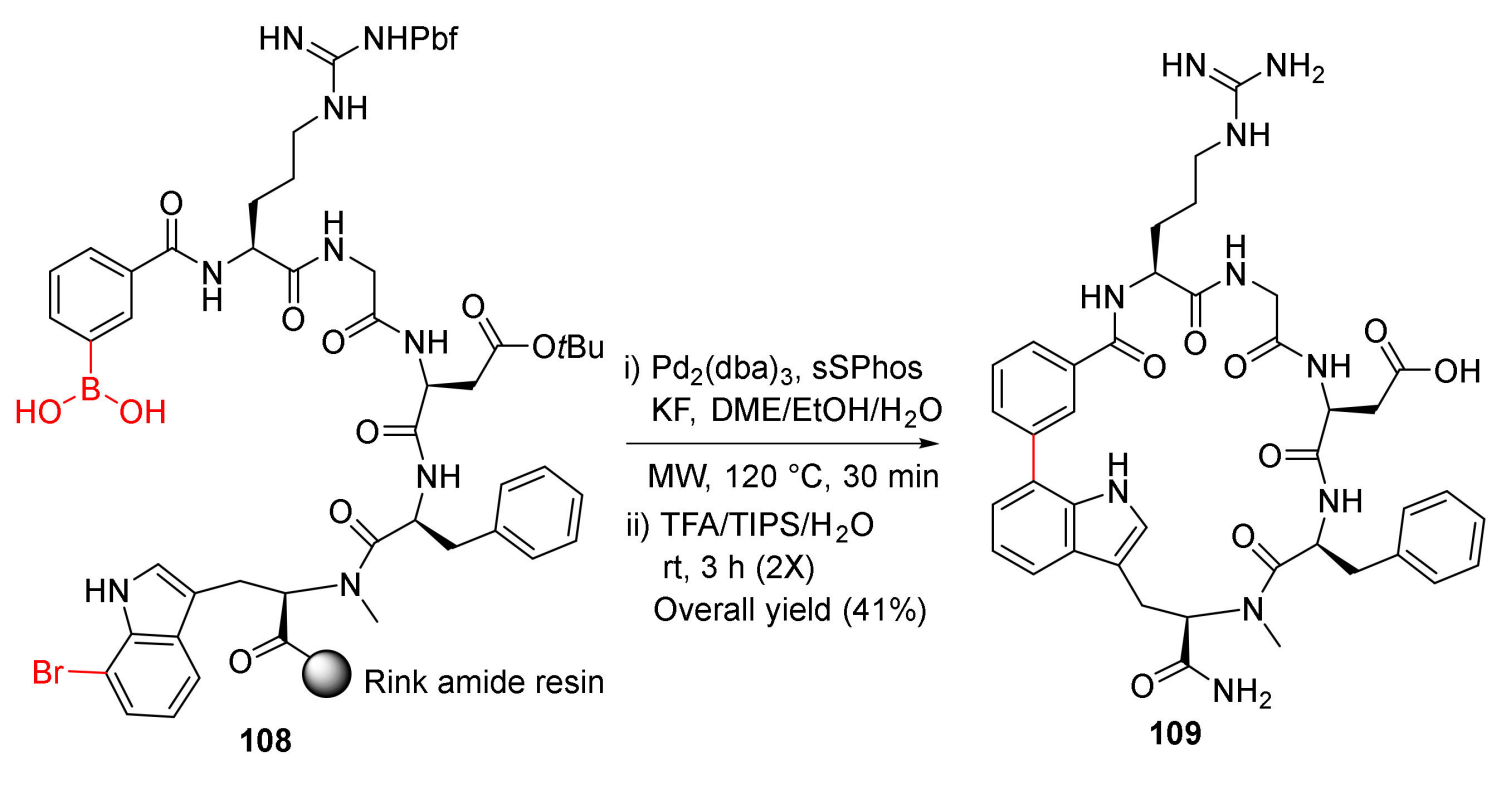
MW-assisted macrocyclization of RGD peptides via Suzuki–Miyaura coupling strategy [109].

Kemker et al. reported the MW-assisted macrocyclization of RGD peptides via Suzuki−Miyaura cross-coupling strategy in 2019 [109]. Solid-supported Suzuki–Miyaura macrocyclization of **108** was performed using Pd_2_(dba)_3_ (20 mol %), sSPhos (40 mol %), and KF (4 eq) in DME/EtOH/H_2_O (9/9/1) at 120 °C under MW irradiation for 30 min, followed by simultaneous resin cleavage and side-chain deprotections in TFA/TIPS/H_2_O (95/2.5/2.5) to provide **109** in a 41% overall yield (Scheme 31) [109]. Macrocycle **109** showed low nanomolar affinity toward α_ν_β_3_ with good selectivity and plasma stability [109]. 

**Scheme 32:**
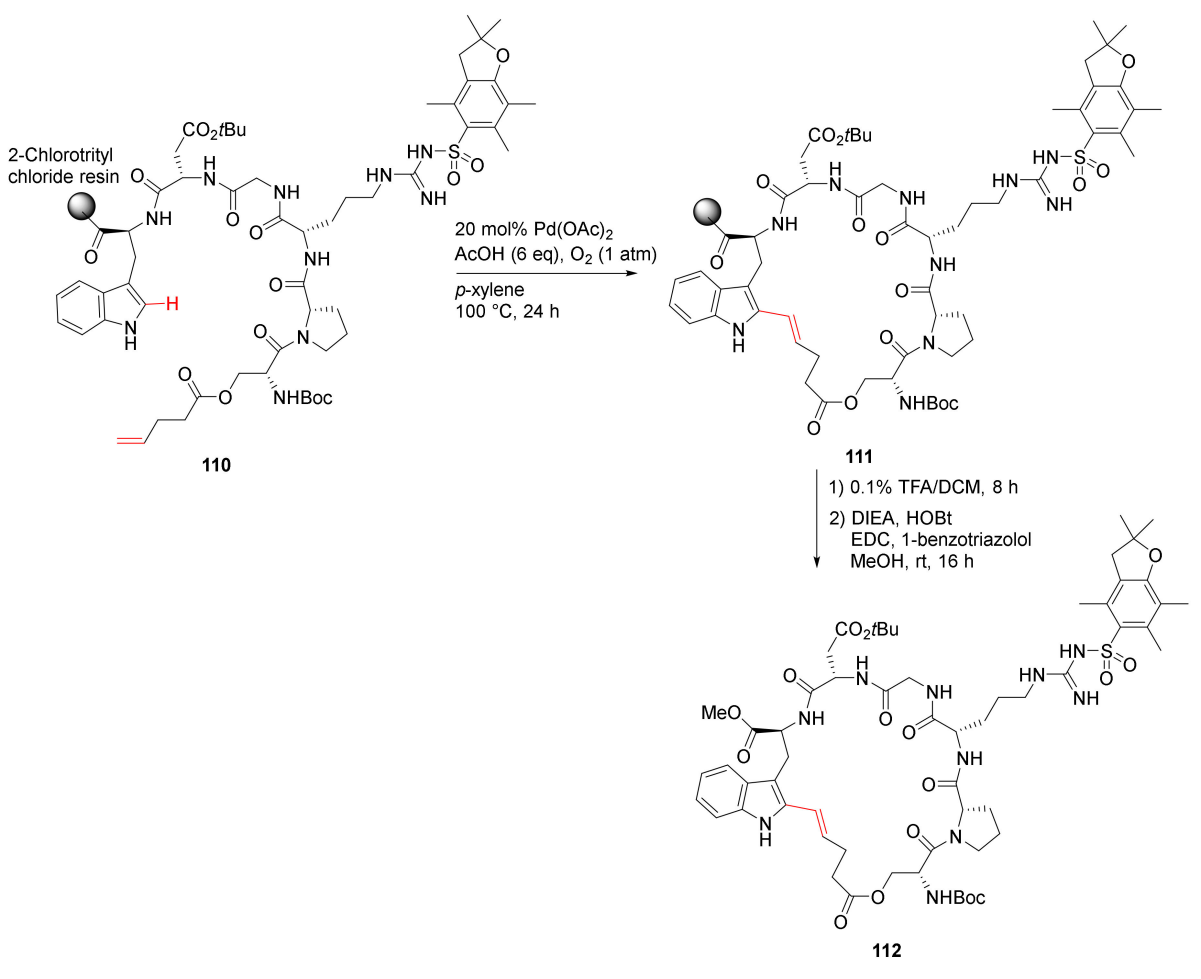
On-resin synthesis of cyclic peptide via Pd-catalyzed site-selective C–H olefination strategy [110].

Bai et al. reported on-resin macrocyclization via Pd-catalyzed site-selective C–H olefination strategy in 2020 [110]. Specifically, backbone-directed Trp(C2) and trifluorosulfonamide (T_f_)-directed Trp(C4) macrocyclization was developed. For example, solid-supported cyclization of **110** was performed using Pd(OAc)_2_ (20 mol %), AcOH (6 eq), and O_2_ (1 atm) in *p*-xylene at 100 °C for 24 h, followed by resin cleavage and subsequent esterification to provide **112** in 26% overall yield (Scheme 32) [110].

**Scheme 33:**
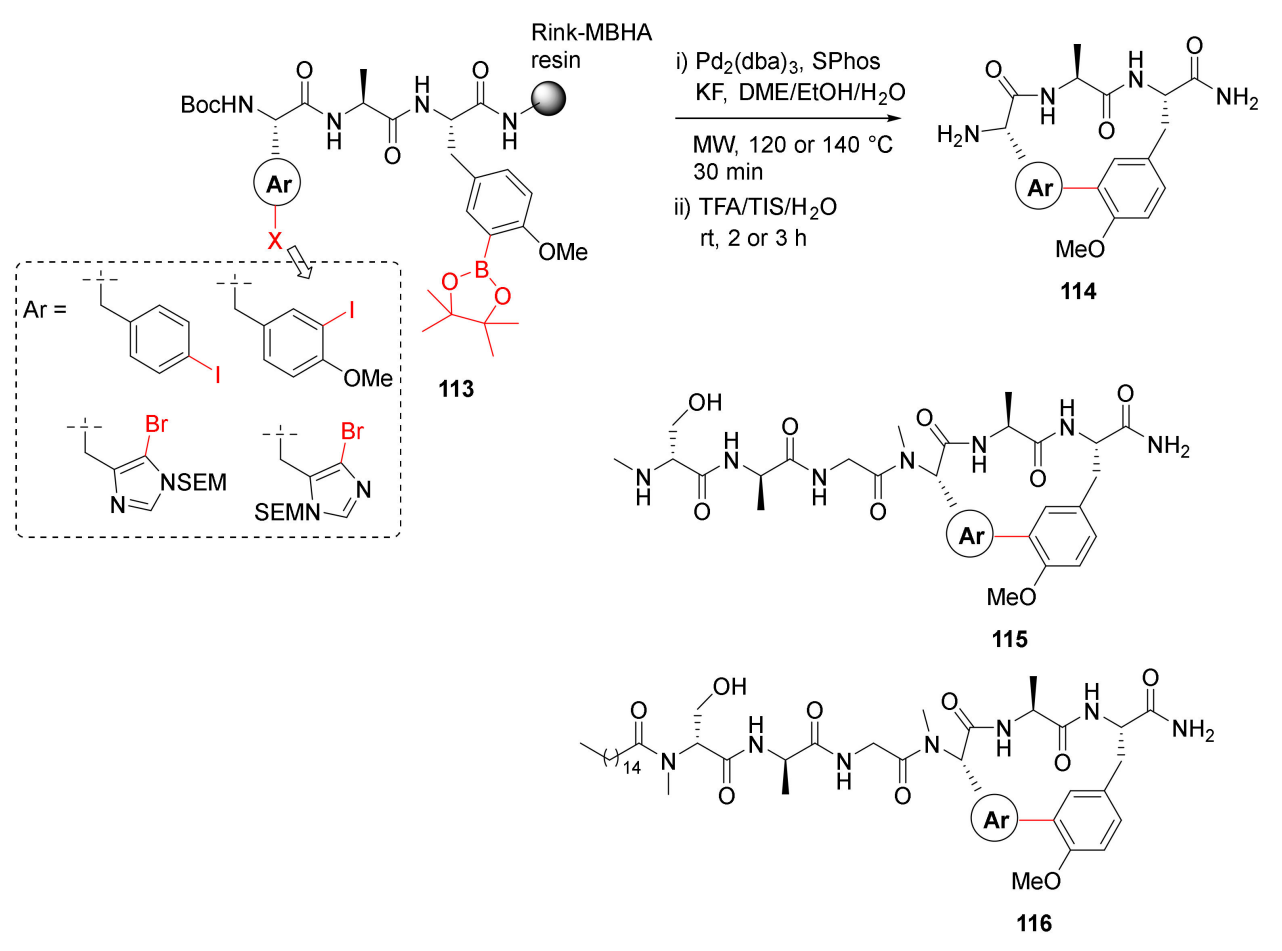
MW-assisted synthesis of biaryl cyclic tripeptides via Suzuki–Miyaura cross-coupling [112].

In order to build on their previous work employing MW-assisted Suzuki–Miyaura macrocyclization reaction [111], in 2020, Ng-Choi et al. reported the MW-assisted synthesis of biaryl cyclopeptides via on-resin intramolecular Suzuki–Miyaura coupling [112]. Suzuki–Miyaura macrocyclization of **113** was performed on solid-support using Pd_2_(dba)_3_ (0.2 eq), SPhos (0.4 eq), and KF (4 eq) in DME/EtOH/H_2_O (9/9/2) at 120 or 140 °C under MW irradiation for 30 min, followed by simultaneous resin cleavage and side-chain deprotections in TFA/TIS/H_2_O to provide **114** (Scheme 33). By using this developed methodology, several other analogs, including cyclolipopeptides **115** and **116,** were synthesized [112].

**Scheme 34:**
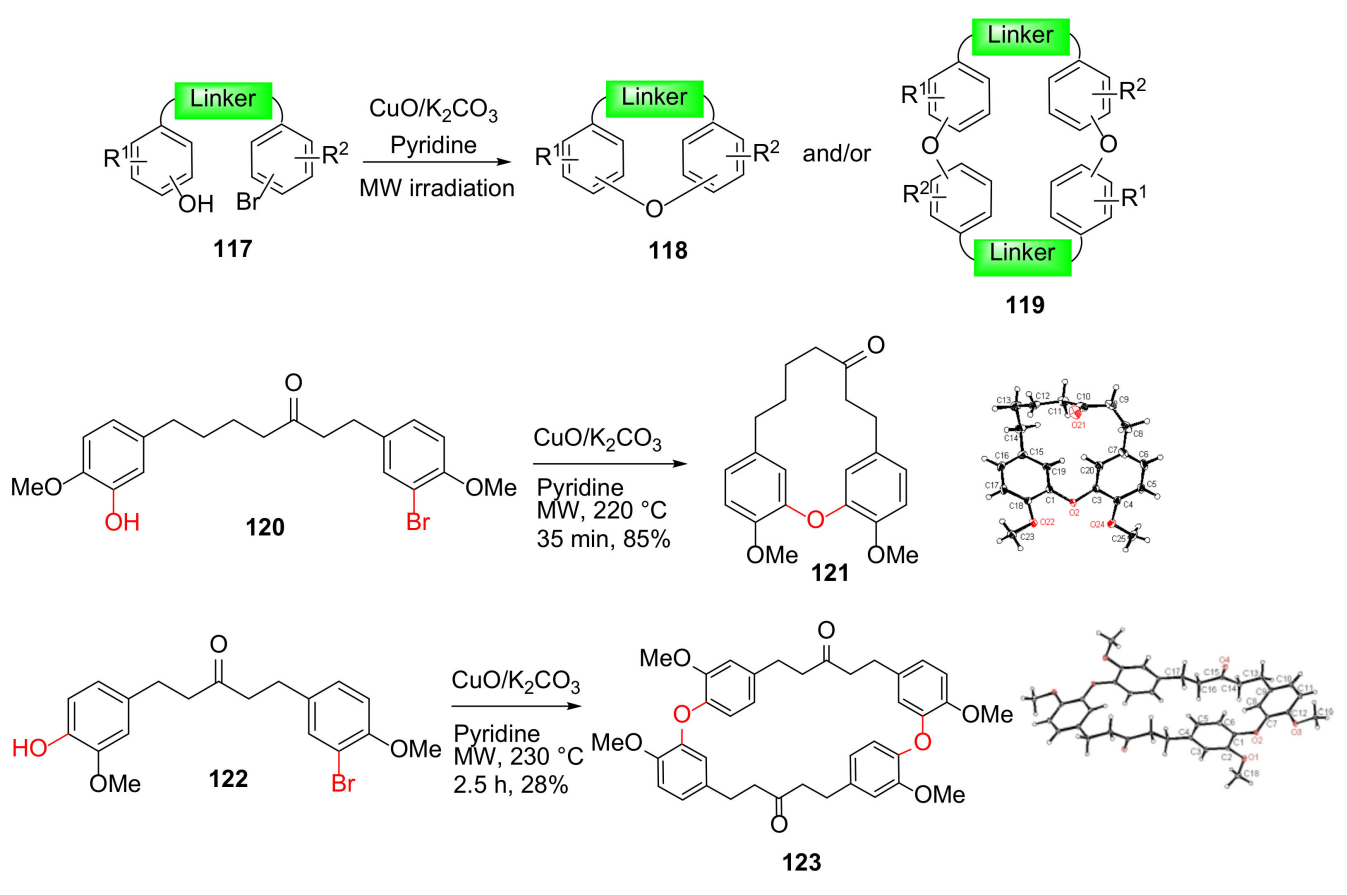
Microwave-assisted intra- and/or bi-molecular Ullmann macrocyclizations [114].

Shen et al. reported the MW-assisted intramolecular Ullmann reaction to synthesize diaryl ether macrocycles **121** and **123** in 2012 (Scheme 34); the MW-assisted Ullmann cyclizations performed more efficiently with higher yields and shorter reaction times compared to sealed pressure tube as well as conventional heating [113,114]. This class of macrocyclic diarylheptanoid analogs and an extended macrocycle library, produced via MW-assisted intramolecular Ullmann coupling, were tested for antibacterial activity, and several macrocycles with phenethyl- and *n*-hexylamino moieties showed antibacterial activity with minimum inhibitory concentrations (MICs) of 12.5–25 μg/mL against *M. tuberculosis*, and Gram-positive organisms [115].

**Scheme 35:**
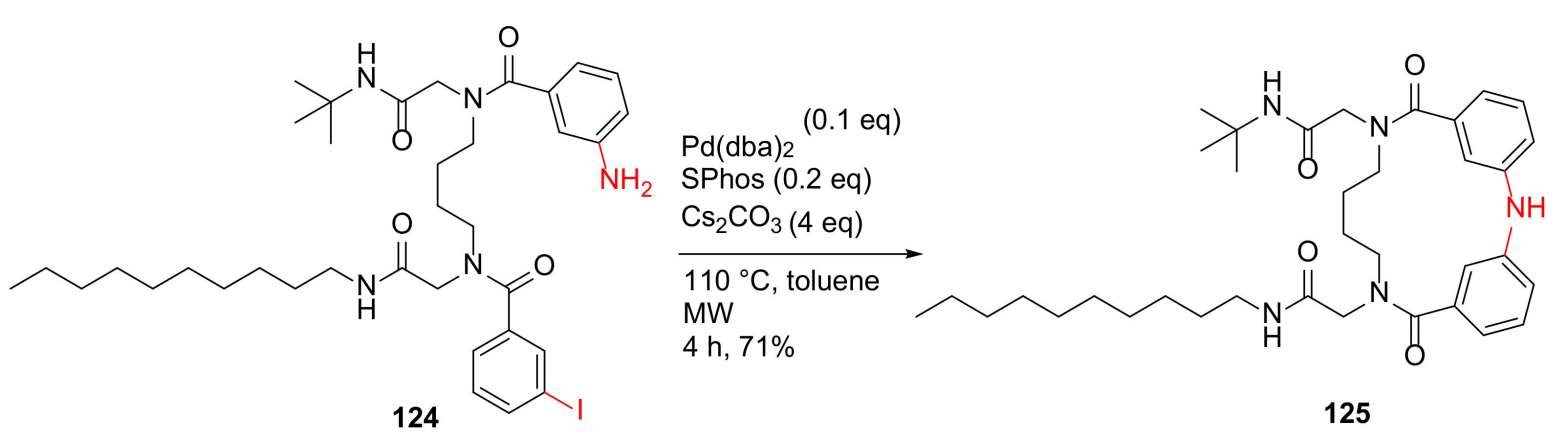
MW-assisted synthesis of a 15-membered macrocycle via Buchwald–Hartwig N-C cross-coupling reaction [116].

Chávez-Riveros et al. reported the synthesis and anti-inflammatory evaluation of fourteen 15-membered diphenylamine macrocycles in 2019 [116]. By using the optimized conditions, diphenylamine macrocycle **125** was synthesized from **124** under MW irradiation at 110 °C for 4 h and using Pd(dba)_2_ as the catalyst, SPhos as the ligand, cesium carbonate as the base in toluene, and in a 71% yield (Scheme 35). Macrocycle **125** with a decylamino side chain showed the most potent anti-inflammatory activity (ID_50_ = 0.18 μM) in a 12-*O*-tetradecanoylphorbol-13-acetate (TPA)-induced edema model with low cytotoxicity [116]. 

### 2.4. Click Macrocyclization via Copper-or Ruthenium-Catalyzed Azide–Alkyne Cycloaddition

**Scheme 36:**
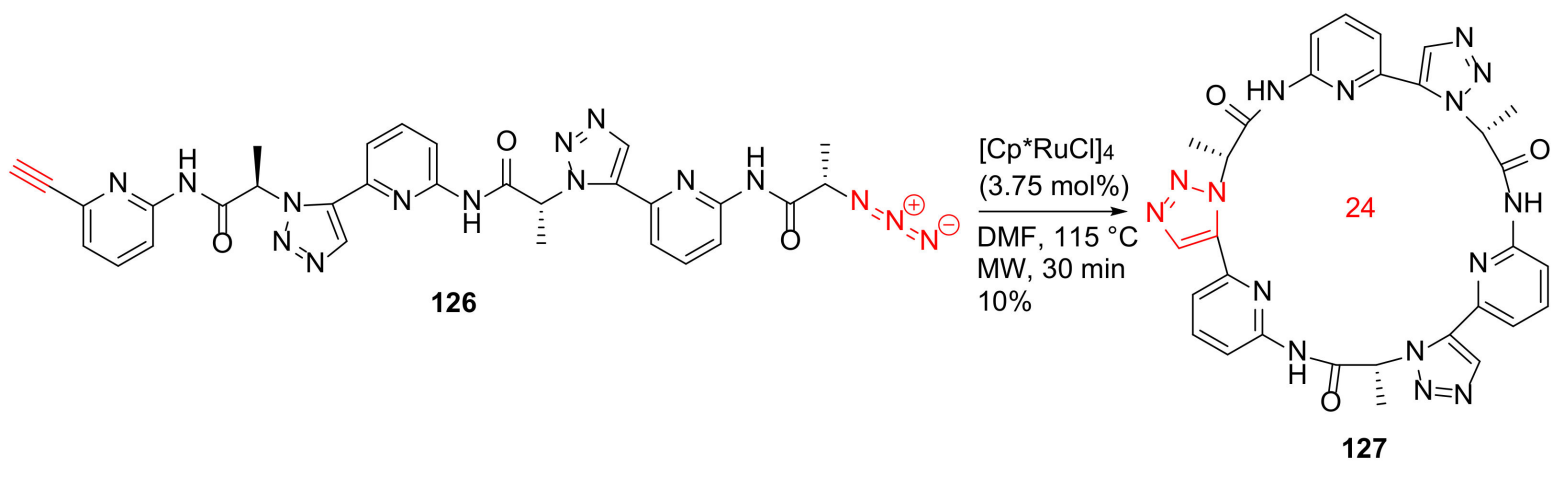
MW-assisted 24-membered macrocyclization of a pseudohexapeptide via the RuAAC click chemistry [117].

Krause et al. reported the MW-assisted macrocyclization of a pseudohexapeptide containing 1,5-disubstituted 1,2,3-triazole moieties, as well as its anion-binding properties in 2011 [117]. Synthesis of **127** was performed from **126** using the [Cp*RuCl]_4_ catalyst in DMF under MW irradiation at 115 °C for 30 min in 10% isolated yield (Scheme 36). In the literature, very few macrocyclizations involve Ru-catalyzed azide–alkyne cycloadditions (RuAAC) to provide a cyclic pseudopeptide derivative [117].

**Scheme 37:**
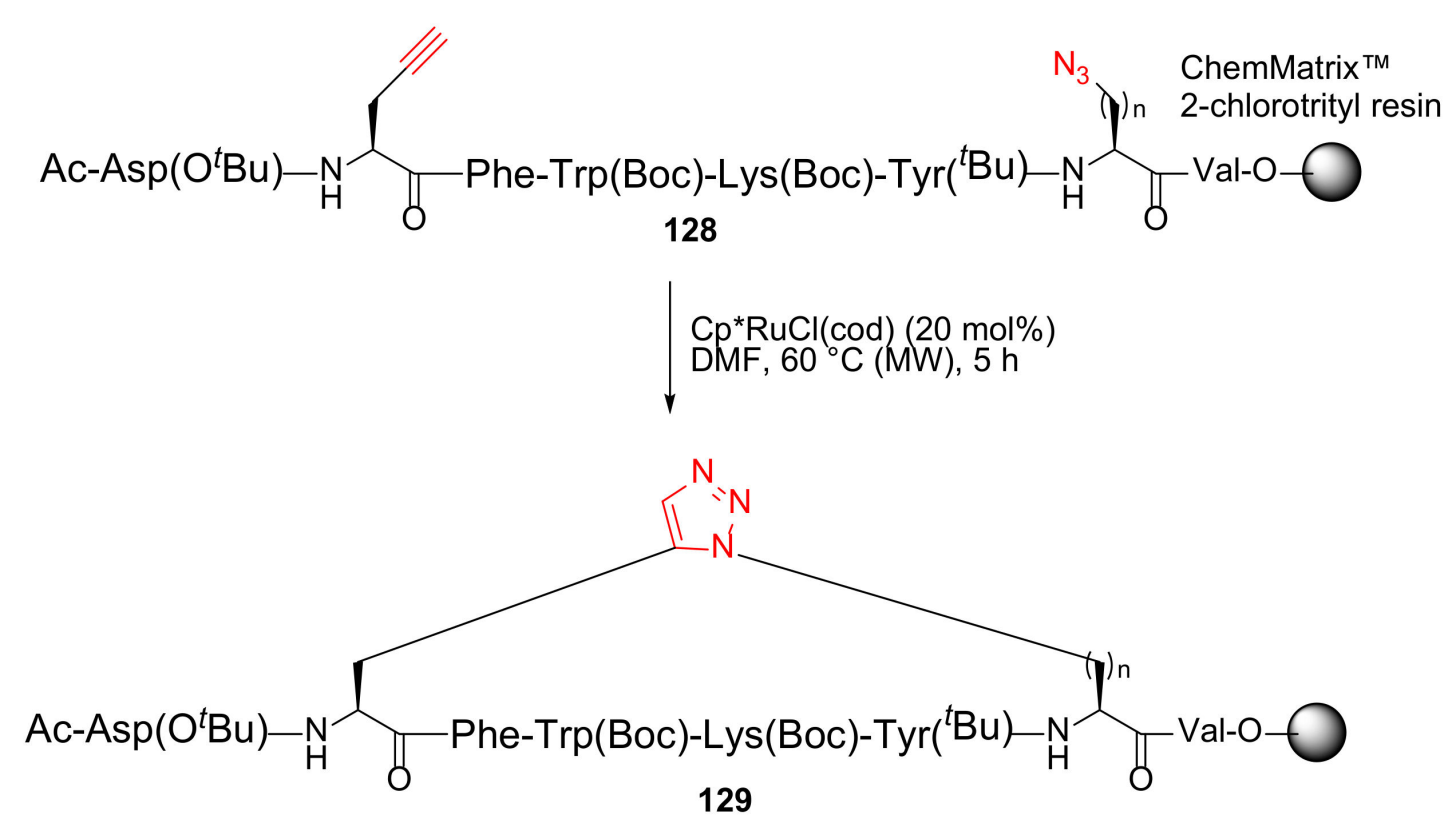
MW-assisted on-resin synthesis of triazole bridged urotensin-II peptidomimetics via side-chain click cyclization strategy [118].

Pacifico et al. reported the MW-assisted on-resin click macrocyclization to synthesize novel triazole bridged Urotensin-II peptidomimetics using RuAAC chemistry in 2017 [118]. Synthesis of 1,5-triazole bridged **129** was performed from **128** using [Cp*RuCl](cod) catalyst in DMF under MW irradiation at 60 °C for 5 h in quantitative yield (Scheme 37) [118].

**Scheme 38:**
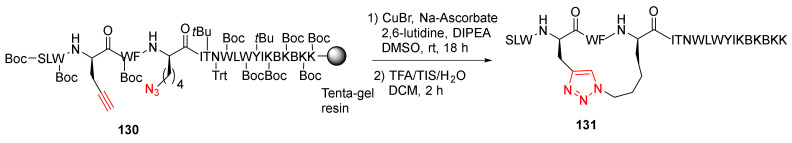
On-resin side-chain click cyclization of complex peptides via the CuAAC strategy [119].

Ingale and Dawson reported the solid-supported side-chain macrocyclization of complex peptides using the Cu-catalyzed azide–alkyne cycloaddition (CuAAC) strategy in 2011 [119]. In this work, a panel of 21 amino acid helical peptides, such as **131**, were synthesized via on-resin intramolecular CuAAC (Scheme 38). Specifically, on-resin CuAAC click macrocyclization of **130** was performed at room temperature in the presence of CuBr (1 eq), sodium ascorbate (1 eq), and 2,6-lutidine (10 eq) in DIPEA/DMSO for 18 h, followed by side chain deprotection and resin cleavage to provide **131** in 65% recovered yield [119].

**Scheme 39:**
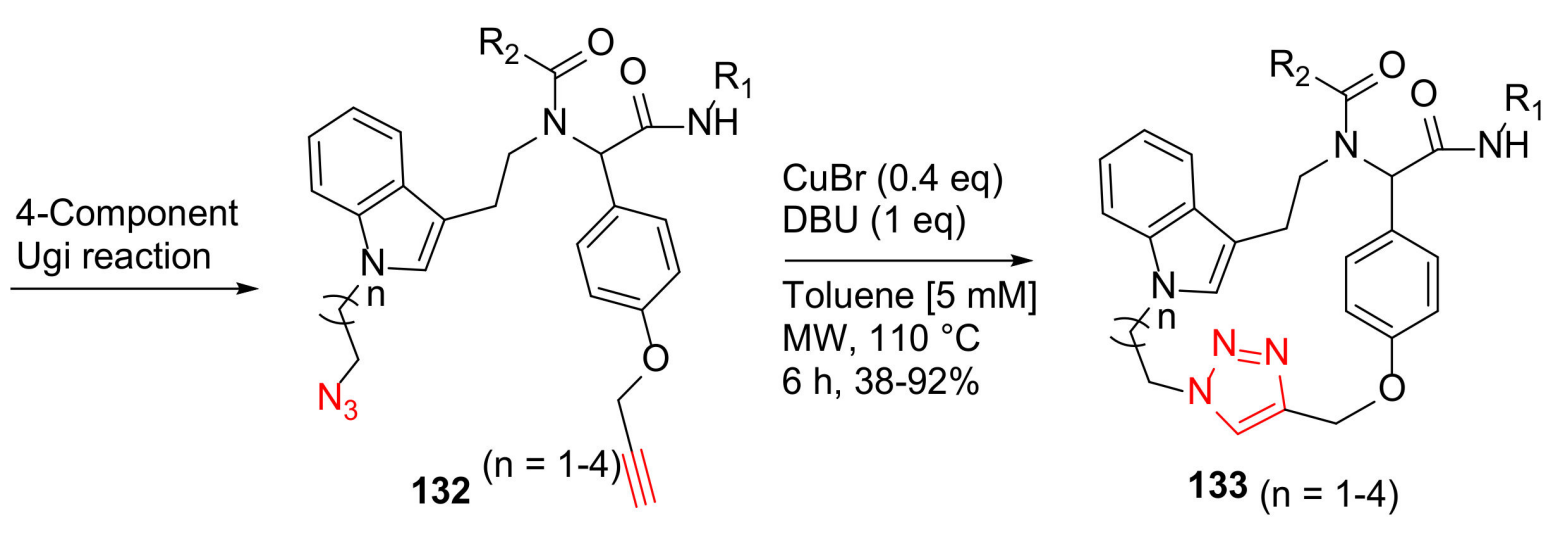
MW-assisted macrocyclization via click cycloaddition chemistry [120].

Chavez-Acevedo and Miranda reported a practical synthesis of novel tryptamine-based macrocycles using sequential 4-component Ugi reaction and MW-assisted click macrocyclization methodology in 2015 [120]. Macrocycles **133**, with a peptoid motif, a 1,3-substituted indole moiety, and a triazole heterocyclic ring, were synthesized from the Ugi reaction product **132** using CuBr (0.4 eq) and DBU in toluene [5 mM] under MW irradiation at 110 °C for 6 h, providing the click chemistry 18- to 21-membered macrocycles **133** in 38–92% yields (Scheme 39) [120].

Among numerous click reactions and applications, the radical-mediated thiol–ene reaction remains an attractive and robust click strategy in organic synthesis and polymer science because of its simplicity, high efficiency, and rapid conversion rate [121]. In 2010, Aimetti et al. reported on-resin peptide photocyclization via thiol–ene click strategy [122].

### 2.5. Intramolecular S_N_Ar or S_N_2 Nucleophilic Substitution

**Scheme 40:**
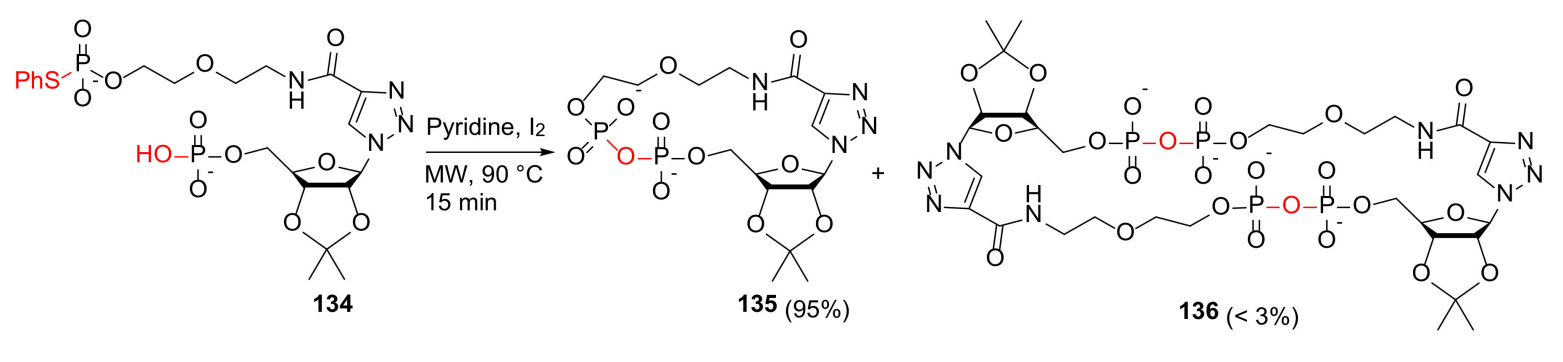
MW-assisted intramolecular cyclopyrophosphorylation [123].

Li et al. reported the MW-assisted concise synthesis and Ca^2+^-mobilizing activity in T-lymphocytes of new nucleobase-simplified cyclic ADP-ribose (cADPR) analogs in 2010 [123]. Synthesis of **135** was performed from **134** in pyridine and I_2_ under MW irradiation at 90 °C for 15 min in 95% yield (Scheme 40) [123]. Notably, under the optimized conditions and MW heating, the yield of the intermolecular pyrophosphoration byproduct **136** decreased to < 3%. Following the acetal deprotection of **135** in 50% HCOOH, the resultant free, cyclic nucleotide bearing the 2’ and 3’-OH groups functions as a membrane-permeable cADPR mimic with calcium release activity in intact T-lymphocytes [123]. By using similar MW-assisted intramolecular pyrophosphorylation methodology, some novel cADPR structural analogs were subsequently synthesized and evaluated for Ca^2+^-mobilizing activity [124,125].

**Scheme 41:**
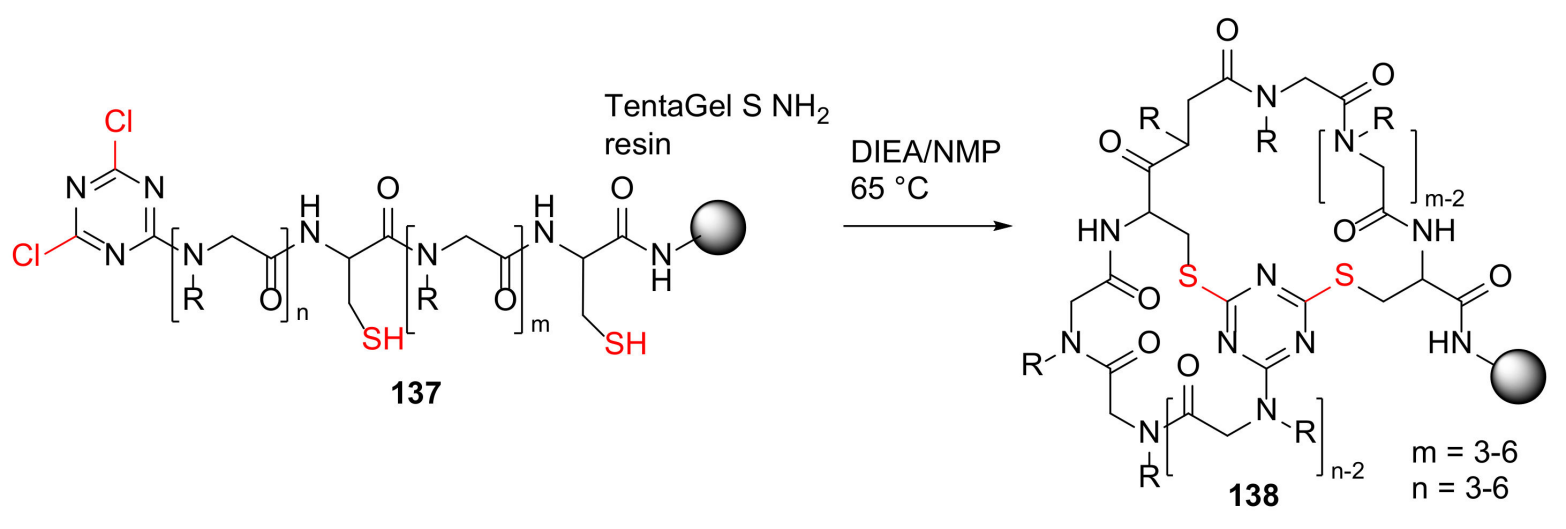
On-resin synthesis of triazine-bridged bicyclic peptoids macrocycle **138** via double nucleophilic attack mechanism [126].

Lee et al. reported the design and solid-phase synthesis of triazine-tethered bicyclic peptoids as conformationally constrained peptidomimetics in 2011 [126]. The resin-bound 6 to 12 peptoid residues **137** was cyclized via double nucleophilic attack to provide new bicyclic peptoids **138** in the presence of DIEA in *N*-methylpyrrolidone (NMP) at 65 °C (Scheme 41) [126].

**Scheme 42:**
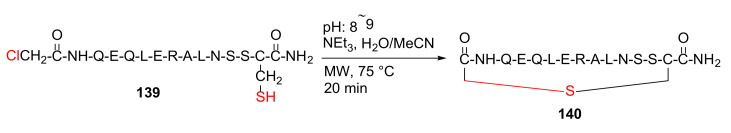
MW-assisted synthesis of the thioether-cyclized peptide [127].

Peng et al. reported the design and synthesis of the helix B surface peptide of erythropoietin and analogs with improved renoprotective effect and metabolic profile in 2013 [127]. Specifically, the peptide precursor was synthesized on a solid support, followed by global side-chain deprotection and resin cleavage to yield linear peptide **139**. Next, MW-assisted macrocyclization of **139** was performed in the presence of triethylamine in water/acetonitrile at 75 °C for 20 min to produce the thioether-cyclized peptide **140** (Scheme 42) [127].

**Scheme 43:**
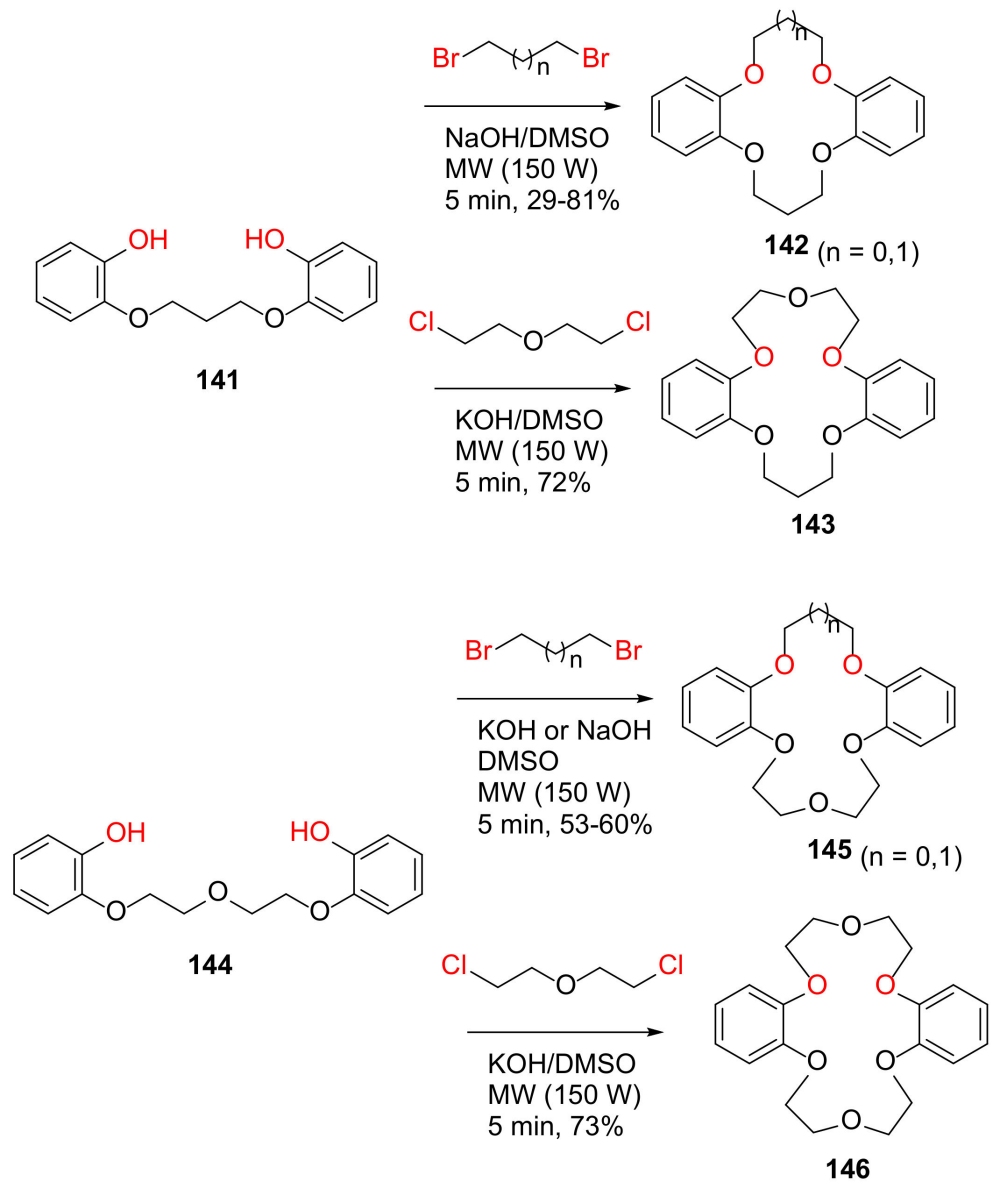
MW-assisted synthesis of dibenzo-crown ethers [128].

Torrejos et al. reported the MW-assisted synthesis of symmetric and asymmetric dibenzo-crown ethers in 2014 [128]. Specifically, symmetric dibenzo-crown ethers **142** (n = 1) and **146** can also be synthesized via one-pot synthesis from catechol and dibromopropane or dichloroethyl ether under basic conditions and MW irradiation with shorter reaction times and fewer purification steps, whereas asymmetric dibenzo-crown ethers **142** (n = 0), **143**, and **145** were obtained from two-step MW-assisted synthesis via the diphenol intermediates **141** and **144**, with satisfactory yields (Scheme 43).

**Scheme 44:**
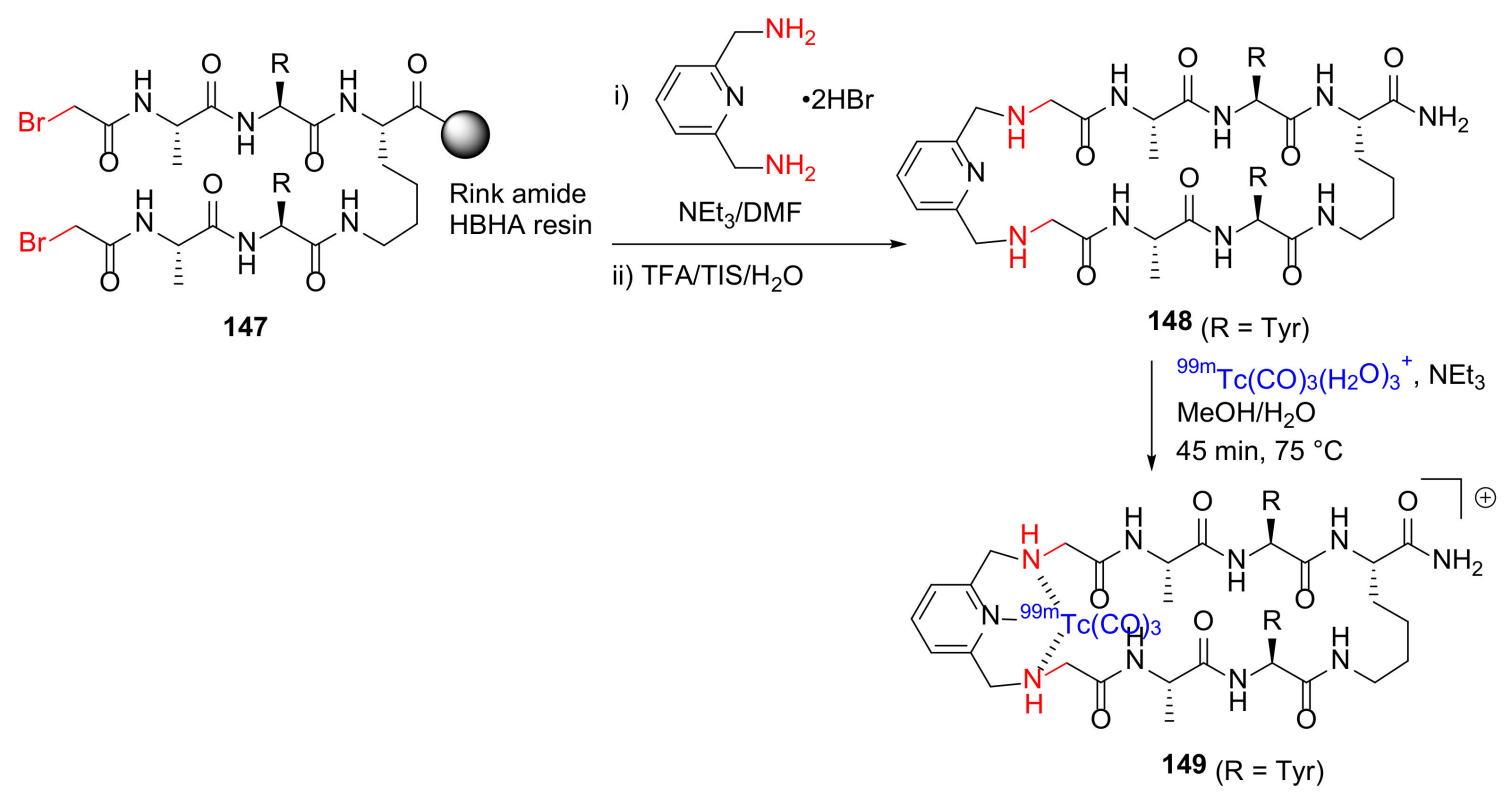
On-resin synthesis of ^99^mTc-containing macrocyclic peptide via a pyridyl tridentate chelation core strategy **[129]**.

Hickey et al. reported the design and synthesis of ^99m^Tc/Re-containing macrocyclic peptides as integrated metal-centric peptidomimetic imaging probes in 2015 [129]. For example, the macrocyclic peptide **148** was synthesized on-resin from **147** and 2,6-bis(aminomethyl)pyridine in DMF with TEA via a pyridyl tridentate chelation core strategy, followed by side chain deprotection, resin cleavage, and metal chelation with [^99m^Tc(CO)_3_(H_2_O)_3_]^+^ to provide **149** (Scheme 44) [129].

**Scheme 45:**
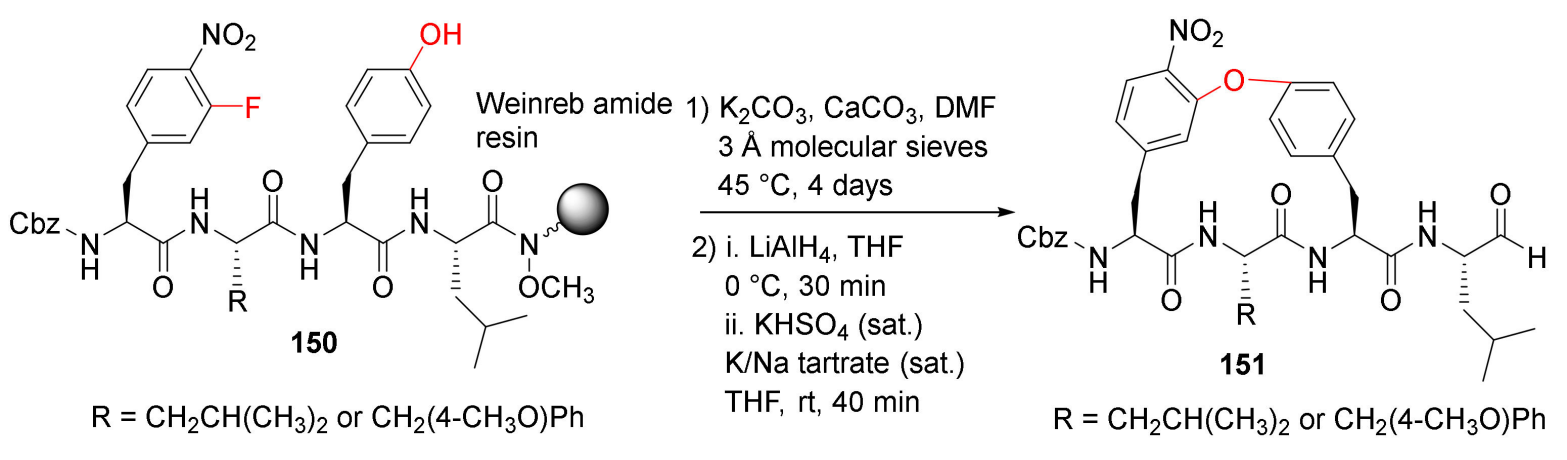
On-resin synthesis of macrocycle **151** via an intramolecular S_N_Ar mechanism [130].

Wilson et al. reported the solid-phase synthesis and biological evaluation of cyclopeptide aldehydes as potent and selective 20S proteasome inhibitors in 2016 [130]. The Weinreb amide resin-bound tetrapeptides **150** were cyclized via an intramolecular S_N_Ar mechanism, followed by the resin cleavage using lithium aluminum hydride (LiAlH_4_) to provide peptide aldehydes **151** (Scheme 45) [130]. The biaryl ether macrocycles **151** represent the first macrocyclic peptide aldehydes with high potency (Ki = 54.5 and 241 nM), cellular stability, and specificity for the proteasome. 

**Scheme 46:**
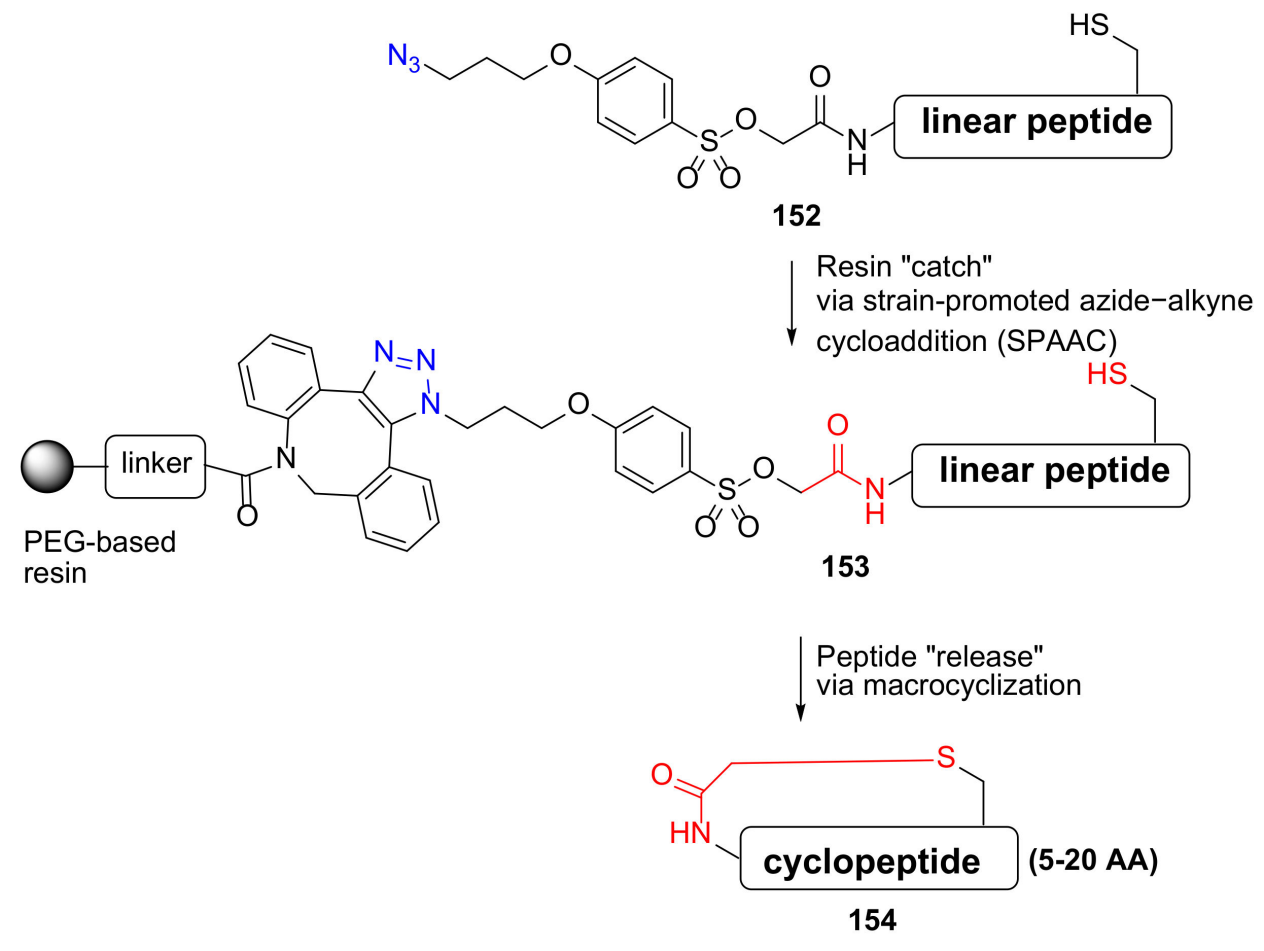
On-resin synthesis of cyclopeptides via a “catch–release” strategy [131].

Kheirabadi et al. reported the synthesis of thioether- or amine-bridged macrocyclic peptides via a “catch–release” strategy in 2018 [131]. This innovative enabling technology was highly effective, producing three macrocyclic peptide libraries containing 5–20 amino acids and thioether- or amine-based macrocyclic linkers (Scheme 46) [131]. Following resin release in 0.1 M NH_3_/MeOH, 5–20 AA membered cyclopeptides **154** were provided with very good purity.

Other diverse on-resin intramolecular S_N_Ar or S_N_2 nucleophilic halide substitution reactions include synthesis of macrocyclic peptides with both α-helix and polyproline helix motifs [132], synthesis of cyclic peptides via an S_N_2 intramolecular thioalkylation [133], synthesis of 19-membered thioether cyclic peptidomimetics [134], synthesis of thiazole-containing cyclopeptides via on-resin intramolecular thioalkylation [135], on-resin synthesis of lipidated macrocyclic, and bicyclic peptides via convenient and intramolecular halide substitution by a diamino acid [136]. In 2018, Zhang et al. reported the on-resin MW-assisted macrocyclization and discovery of bisthioether-stapled peptides with excellent proteolytic stability and potent inhibition of PRC2 catalytic activity [137]. In 2021, Roy et al. reported a powerful, high-throughput quality control assay for on-resin synthesis and analysis of macrocyclic DNA-encoded libraries via thioalkylation strategy [138].

### 2.6. Condensation Reactions

**Scheme 47:**
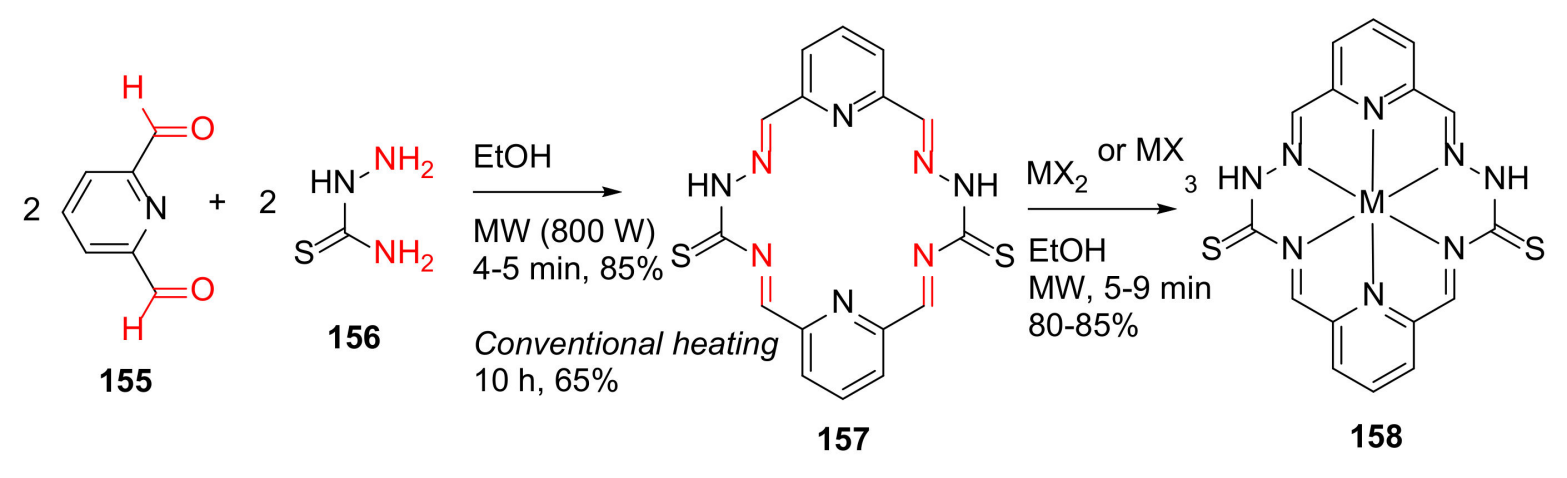
MW-assisted synthesis of 18-membered macrocycle metal complexes [139].

Ahmed et al. reported the MW-assisted synthesis and antibacterial evaluation of metal complexes with Schiff base 2, 6-pyridinedicarboxaldehydethiosemicarbazone (PDCTC) in 2014 [139]. Specifically, macrocyclic ligand **157** was synthesized from 2, 6-pyridinedicarboxaldehyde **155** and thiosemicarbazone **156** in dry ethanol under MW irradiation for 4–5 min, followed by the formation of Cu, Co, and Ni (II), and Cr (III) metal complexes **158** of these macrocycle ligands (Scheme 47). The metal complexes are generally more active than their parent Schiff base ligand in antibacterial testing [139]. 

**Scheme 48:**
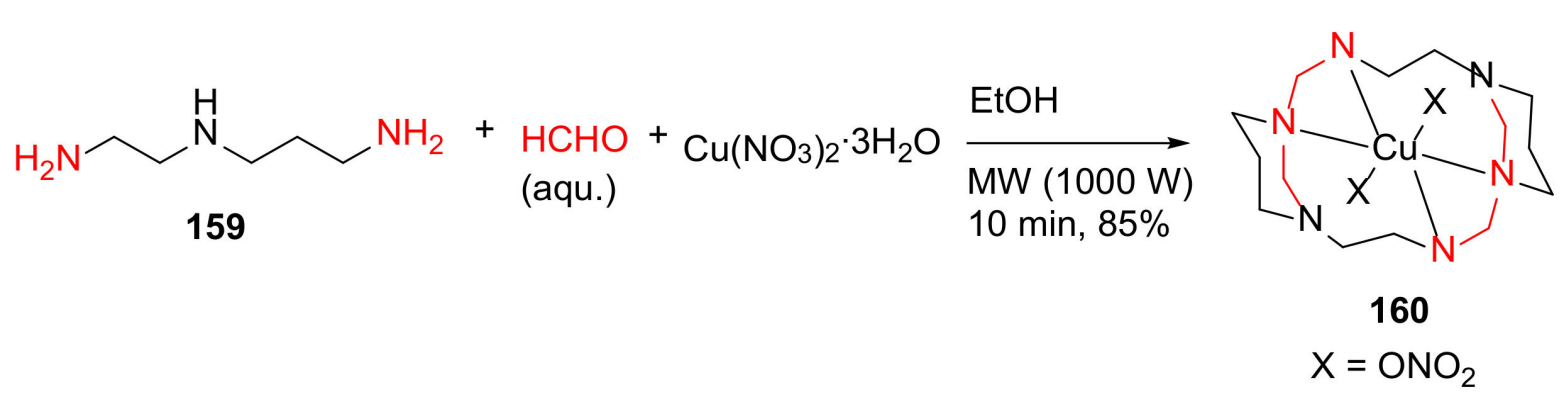
MW-assisted synthesis of diazacyclam-based macrocyclic copper complex [140].

Hakimi et al. reported the MW-assisted template synthesis, geometry studies, and photoluminescent properties of the diazacyclam-based macrocyclic copper complex in 2014 [140]. Specifically, the macrocyclic copper complex **160** of 1,3,6,10,12,15-hexaazatricyclo[13.3.1.1^6,10^]eicosane (L), [CuL(NO_3_)_2_], was synthesized from *N*-(2-aminoethyl)-1,3-diaminopropane **159**, formaldehyde and copper(II) nitrate in one-pot under MW irradiation for 10 min in 85% yield (Scheme 48) [140]. Several other related metal complexes including [CuL]Br_2_, [CuLI][CuLI]’I_2_·H_2_O, and [[CuL(NO_3_)]_2_[Hg_5_(*µ*-Cl)_8_(*µ*^3^-Cl)_2_]Cl_2_]n were also synthesized from **159** by replacing the nitrate ions with bromide, iodide or by reacting **160** with HgCl_2_, respectively, and investigated in this work [140].

**Scheme 49:**
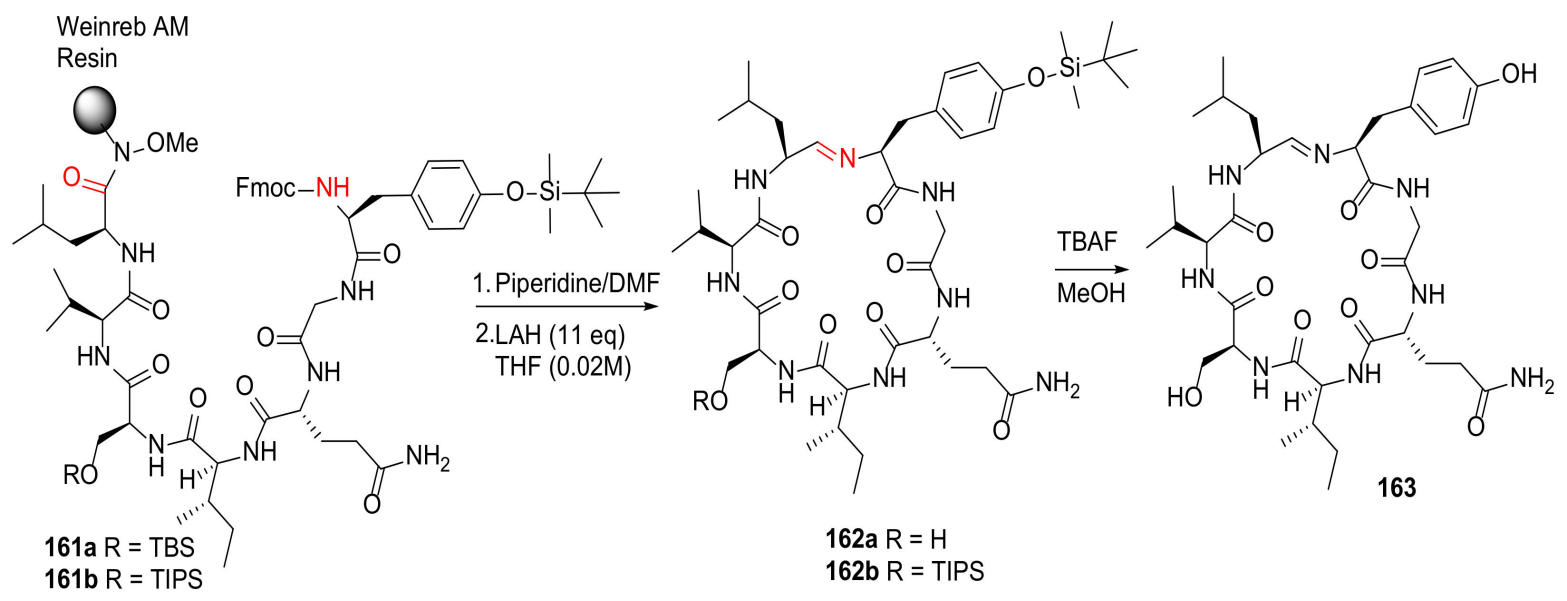
Total synthesis of scytonemide A via a biomimetic solid-phase synthesis strategy on the Weinreb AM resin [141].

Wilson et al. reported the solid-phase total synthesis of scytonemide A employing Weinreb AM resin in 2018 [141]. Following Fmoc deprotection, macrocyclization of the protected heptapeptide **161** was achieved via spontaneous imine formation upon resin cleavage using a reducing agent lithium aluminum hydride (LAH) and subsequent aqueous workup (Scheme 49). The final deprotection of **162** was performed under TBAF in methanol, providing scytonemide A (**163**) in a 51% yield [141].

**Scheme 50:**
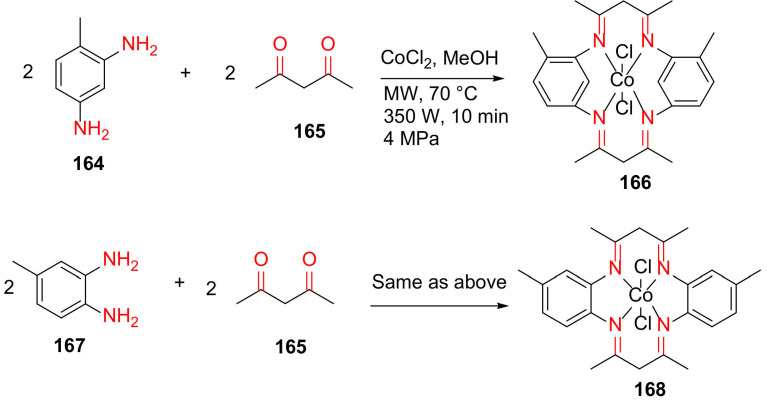
Microwave-assisted synthesis of Co^II^HMTAA-14/16 macrocycles [142].

Kumar and Vashistha reported the MW-assisted synthesis of Co^II^HMTAA-14/16 macrocycles and their nanocomposites with highly conductive carbon black in 2019 [142]. Specifically, **166** and **168** were synthesized from acetylacetone **165**, cobalt(II) chloride hexahydrate, and 2,4-/3,4-diamino toluene **164** or **167** in methanol at 70 °C for 10 min under MW irradiation (Scheme 50). These macrocycles were further used to synthesize their nanocomposites in the presence of carbon black and ethanol under MW irradiation (350 W, 4 MPa, 120 °C) for 30 min [142].

**Scheme 51:**
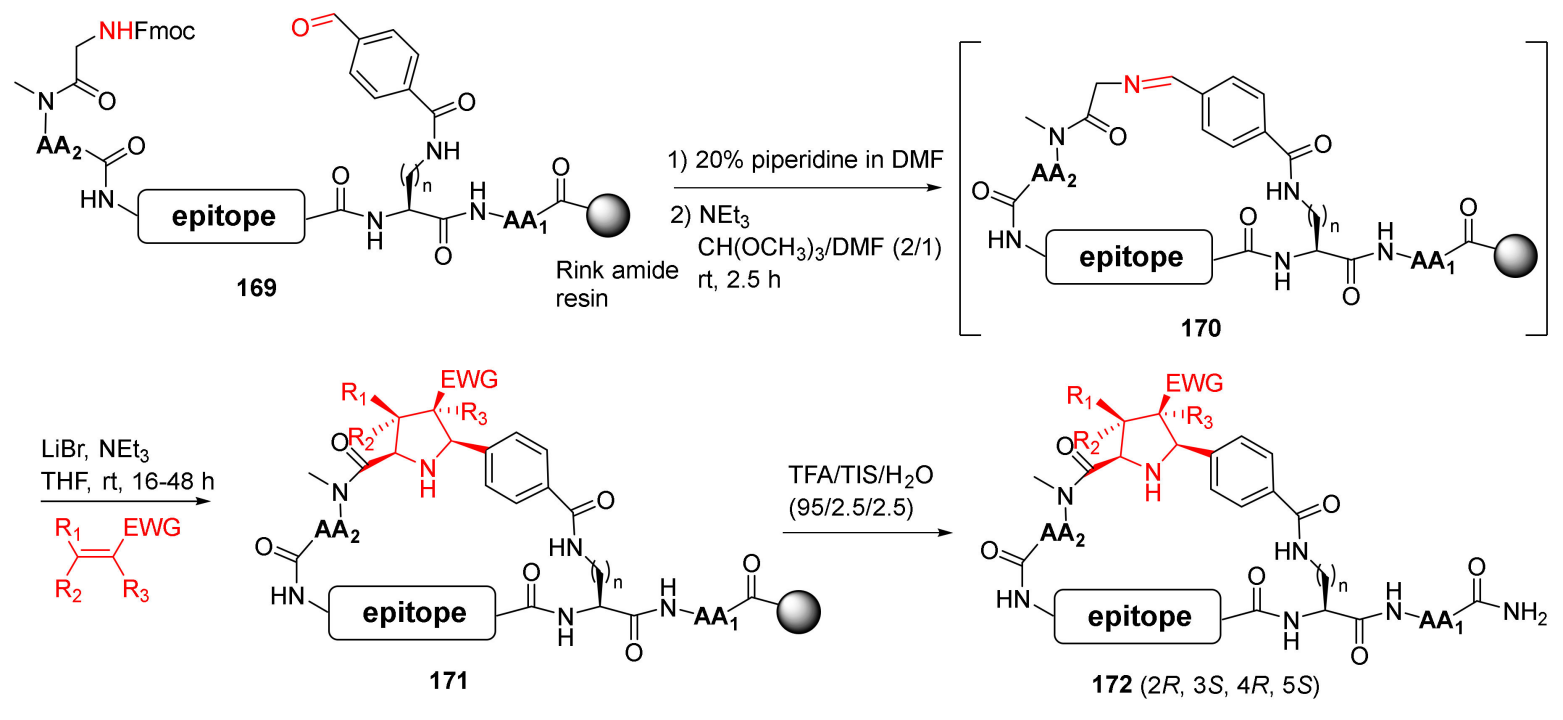
On-resin synthesis of cyclic PepNats via the imine/cycloaddition strategy [143].

Guéret et al. reported the solid-phase synthesis of macrocyclic modalities containing peptide epitopes and natural product motifs via macrocyclization by imine formation and subsequent stereoselective 1,3-dipolar cycloaddition in 2020 [143]. Specifically, a one-pot solid-phase synthesis protocol was developed to deprotect the Fmoc group in **169** and generate Schiff base **170** using trimethyl orthoformate as the dehydrating agent. Subsequent intramolecular cyclization of **170** was performed to provide **171** via azomethine ylide formation and 1,3-dipolar cycloaddition in the presence of lithium bromide (Scheme 51). Following the removal of side-chain protecting groups and simultaneous release from the resin, the major diastereomer **172** of the desired cycloadducts was produced in overall 8−14% purified yields [143].

### 2.7. Multi-Component Reaction (MCR)-Mediated Macrocyclization

**Scheme 52:**
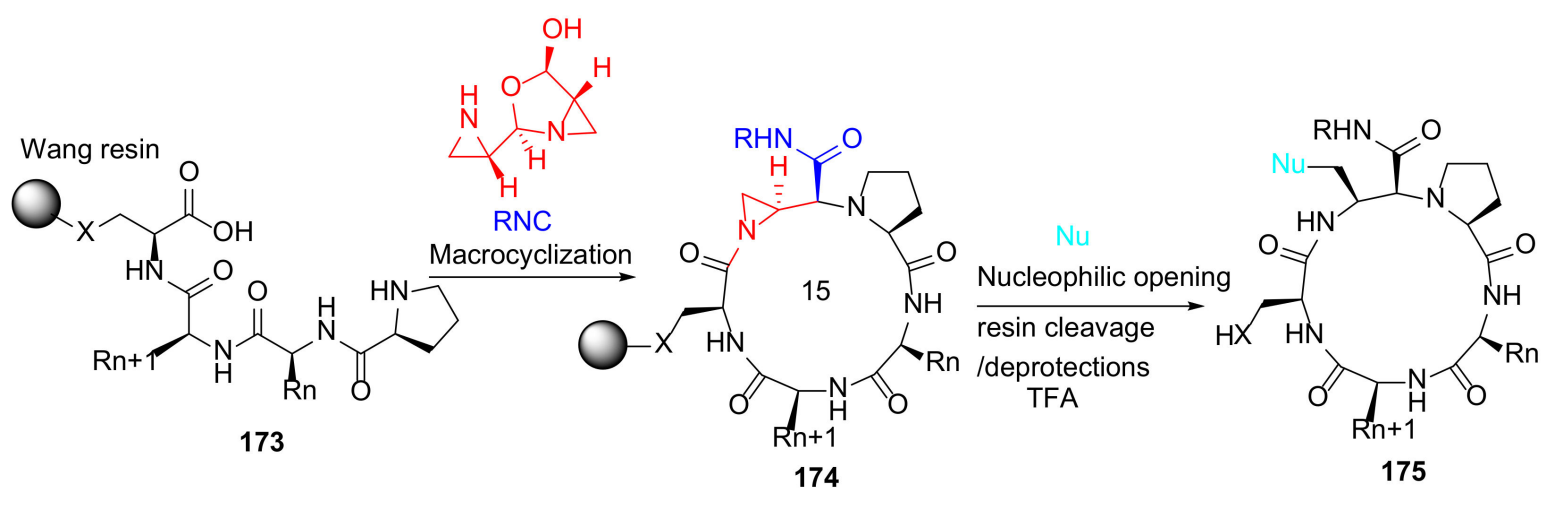
On-resin synthesis of macrocyclic peptidomimetics via three-component coupling strategy [144].

Treder et al. reported the first solid-phase synthesis of functionalized, macrocyclic peptidomimetics via three-component coupling mediated by aziridine aldehyde dimers in 2015 [144]. On-resin macrocyclization of **173** was achieved using aziridine aldehyde dimer and an appropriate isocyanide, providing **174**, which was subsequently functionalized by the nucleophilic opening of the aziridine ring (Scheme 52). This developed methodology enables the rapid parallel synthesis of macrocyclic peptides **175 [144]**.

**Scheme 53:**
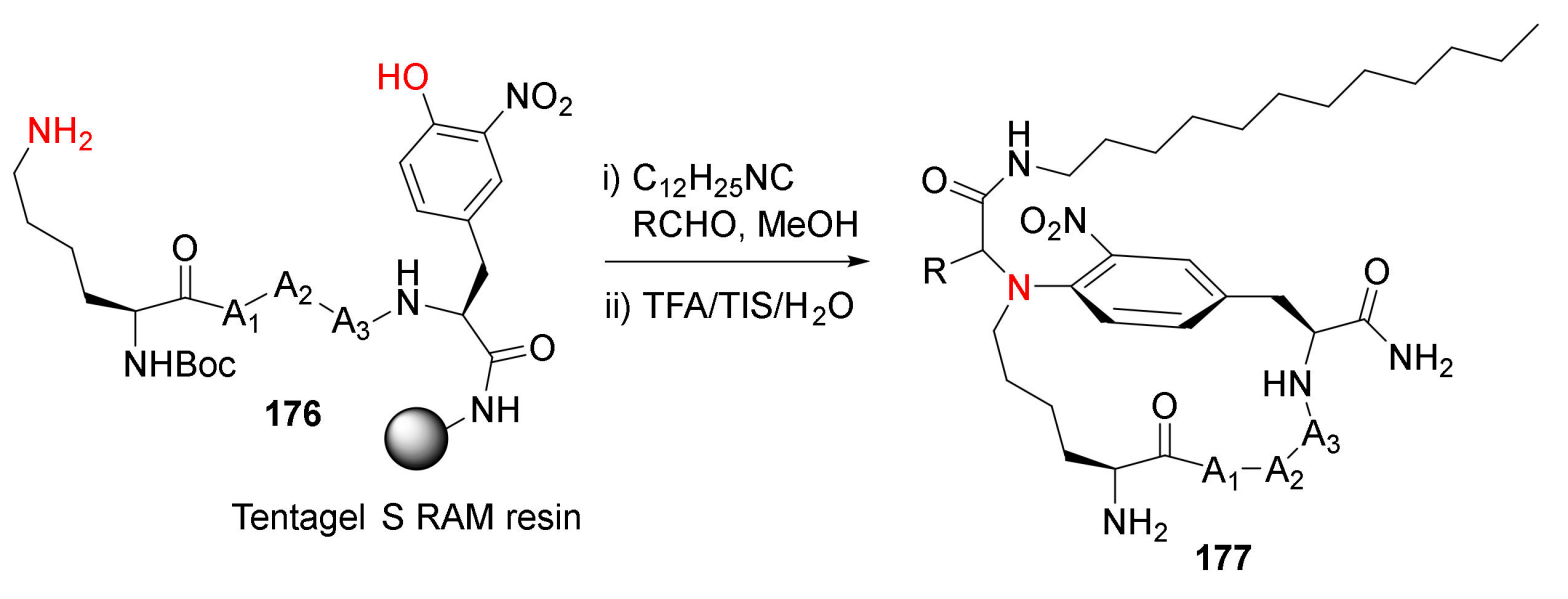
Solid-phase synthesis of *N*-aryl-bridged cyclolipopeptides via Ugi−Smiles macrocyclization methodology [145].

Morejón et al. reported the solution- and solid-phase synthesis of *N*-aryl-bridged cyclolipopeptides via the Ugi–Smiles multicomponent methodology in 2016 [145]. Solid-supported Ugi–Smiles macrocyclization of 3-nitrotyrosine-containing peptide **176** took place effectively in the presence of an isocyanide and an aldehyde, followed by simultaneous resin cleavage and side-chain deprotections in TFA/TIS/H_2_O to provide **177** in good yields (Scheme 53) [145]. Solid-supported Ugi-4-component macrocyclization methodology was subsequently reported to synthesize polycationic cyclolipopeptides with stabilized helical structures and antimicrobial properties [146], short and medium-size canonical cyclopeptides with turn-inducing moieties [147], stabilized cyclic β-hairpins, and *N*-alkylated peptides with various exo-cyclic functionalization such as cationic or hydrophobic tails and bioconjugation handles to install fluorescent and affinity tags, etc. [148]. In 2019, Reguera and Rivera reviewed the applications of the MCR toolbox for peptide macrocyclization and stapling [149]. In 2021, Rivera et al. reviewed solid-phase multicomponent protocols for biopolymer assembly and derivatization (e.g., the use of MCRs in the traceless on-resin synthesis of cyclic peptides) [150]. New synthetic methodology via on-resin Ugi reaction-based macrocyclization of peptide side chains to exocyclic functionalized helical peptides was subsequently developed by the same research team [151].

**Scheme 54:**
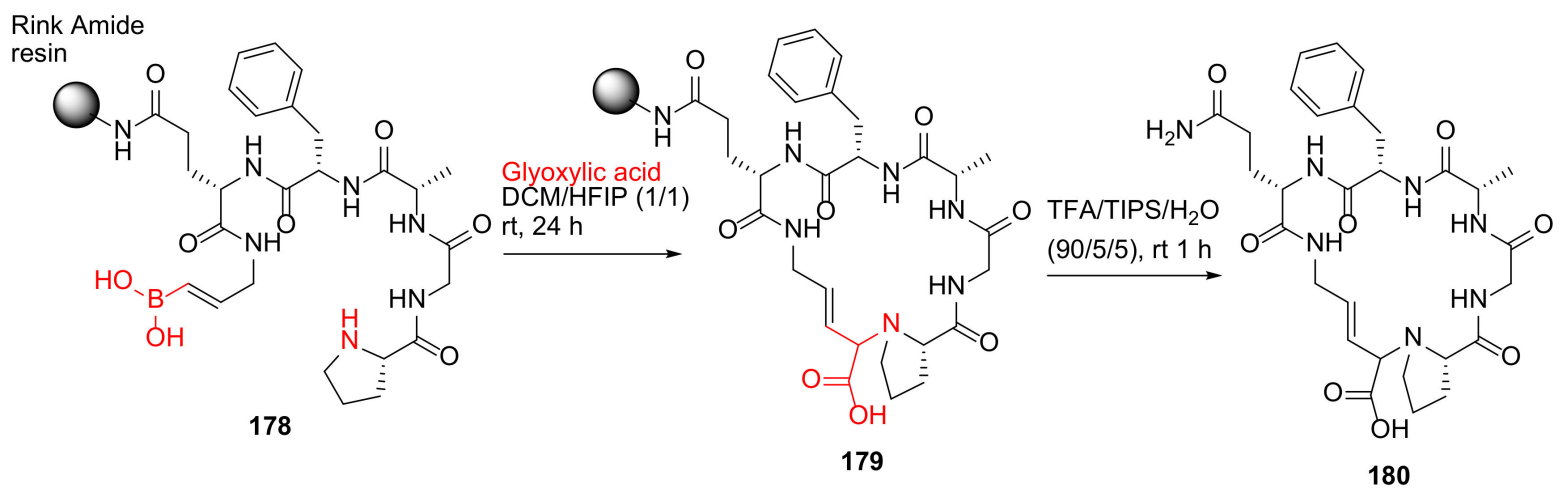
On-resin synthesis of macrocyclic peptides via the Petasis borono–Mannich cyclization strategy [152].

Yamaguchi et al. reported the synthesis of conformationally stable macrocyclic peptides via the Petasis borono–Mannich cyclization strategy in 2019 [152]. A representative example is shown in Scheme 54. Specifically, the Petasis borono–Mannich macrocyclization was carried out from **178** and glyoxylic acid in DCM/HFIP at room temperature for 24 h, followed by resin cleavage in TFA/TIPS/H_2_O at room temperature for 1 h to provide the desired product **180** [152].

**Scheme 55:**
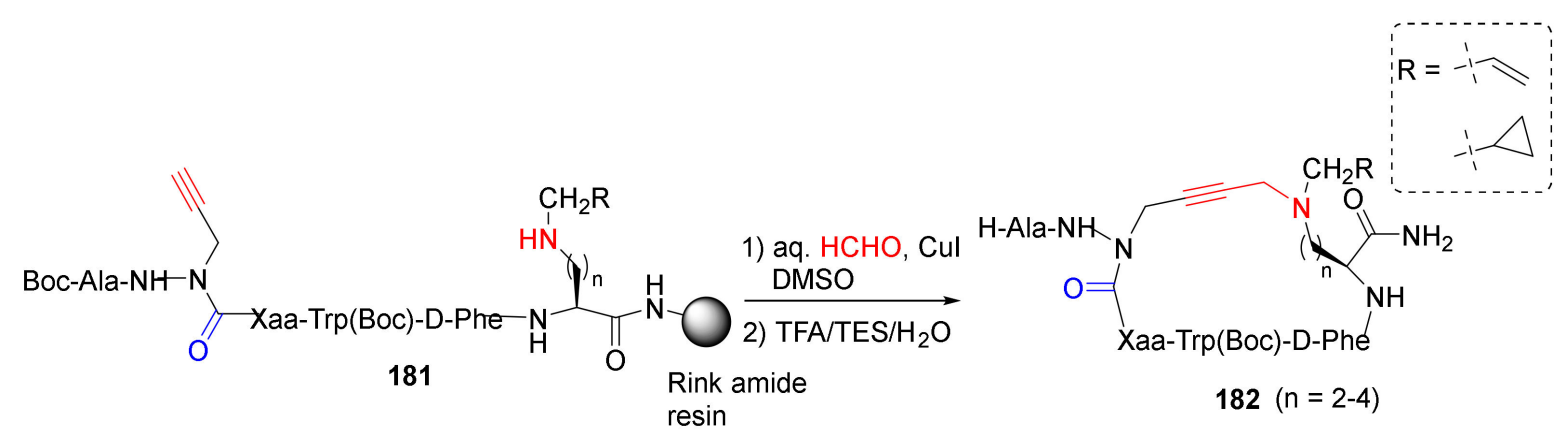
Diversity-oriented on-resin synthesis of cyclic azapeptides via A^3^-macrocyclization strategy [153].

Ohm et al. reported the diversity-oriented A^3^-macrocyclization of cyclic peptides with different substitution, ring size, and shape as CD36 receptor modulators in 2021 [153]. Specifically, cyclic azapeptides **182** were synthesized from linear precursors **181** using A^3^-macrocyclization strategy in aqueous formaldehyde and CuI in DMSO, followed by the resin cleavage and side-chain deprotection using a TFA/TES/H_2_O cocktail (Scheme 55) [153]. Previously, the same research group studied the dynamic chirality of this cyclic azapeptide class of allosteric CD36 modulators of macrophage-driven inflammation using the same macrocyclization approach [154], aza-propargylglycine installation via aza-amino acylation strategy [155], and diverse cyclic azapeptides as CD36-modulating peptidomimetics via A3-macrocyclization approach [156].

## 3. Conclusions

Macrocycles offer some key attributes and advantages over small molecules and natural products such as favorable PK/PD parameters and improved oral bioavailability, enhanced metabolic stability, and cell permeability, and thus play an important role in chemical biology and medicinal chemistry. Since 2014, nineteen macrocyclic drugs and radiopharmaceuticals have been approved by FDA in the therapeutic areas of bacterial and viral infections, oncology, immunosuppression, etc. Remarkably, among them, five macrocyclic NS3/4A protease inhibitors have been approved for the treatment of HCV infections either alone or in combination with other viral medications. However, several of these agents were discontinued from the US due to clinical practice changes or other reasons in the arena of chronic HCV treatment, including newer, safer, and/or more effective treatment options.

Undoubtedly, macrocycles continue to represent an exciting chemical class in clinical drug development pipelines, exemplified by a tri-macrocyclic phase II clinical candidate, a proprotein convertase subtilisin/kexin type 9 (PCSK9) inhibitor, for lipid-lowering therapy [157]. This tricyclic peptide PCSK9 inhibitor has the potential to be developed as an oral once-daily lipid-lowering agent as the only other two FDA-approved PCSK9 drugs, Praluent (alirocumab) and Repatha (evolocumab), are monoclonal antibodies (biologics), which are administered subcutaneously. Notably, it remains a challenge to develop macrocyclic peptides with oral bioavailability for systemic use. Other macrocyclic orally bioavailable peptide drugs include immunosuppressants Gengraf, Neoral, Sandimmune (cyclosporine), and Lupkynis (voclosporin). In the case of macrocyclic peptide drug Trulance (plecanatide) with two intramolecular disulfide linkages, it is taken orally but with minimal GI absorption and thus used for the treatment of GI-related disorders (e.g., CIC and IBS-C). In this context, such other recently approved macrocyclic oral drugs with minimal absorption include antibacterial Aemcolo (rifamycin SV) for managing and treating travelers’ diarrhea and Dificid (fidaxomicin) for the treatment of adult patients with *C. difficile* infections [13]. In addition, several advanced macrocycle clinical candidates were developed using the macrocycle–antibiotic hybrid strategy [5].

Moreover, high throughput chemistry, modular synthesis, and new macrocyclization methodology enable facile, rapid synthesis, SAR, and new advances in macrocycle drug discovery. Specifically, microwave-assisted and/or on-resin macrocyclizations have a profoundly accelerated generation of macrocycle libraries. These were highlighted via various recent MW-assisted and/or solid-supported macrocyclization strategies, including macrolactonization and macrolactamization, transition-metal catalyzed olefin ring-closure metathesis, intramolecular C–C and C–heteroatom cross-coupling, copper- or ruthenium-catalyzed azide–alkyne cycloaddition, intramolecular S_N_Ar or S_N_2 nucleophilic substitution, condensation reactions, and multi-component reaction-mediated macrocyclization. Depending on the nature of reaction substrates and types, both MW irradiation and solid-phase synthesis can shorten reaction times and synthetic routes, enhance reaction efficiency with higher yields and fewer side products, improve catalyst performance, consume less solvent, and/or streamline the synthesis and overall purification process. Though in some cases, significant optimization studies are required to find the most optimal reaction conditions and reagents. In multiple cases, on-resin MW-assisted macrocyclizations were effectively applied to the macrolactamization of peptide and peptidomimetics, RCM, triazole click chemistry, and intramolecular C–C coupling. Other innovative macrocycle entities and new advances in synthetic methodology development include the MCR-mediated macrocyclizations. Collectively, this work reviews and showcases some exciting advances in the macrocycle space, and its significance and impact will continue to thrive in the areas of chemistry, biology, and medicine.

## Figures and Tables

**Table 1:** Key attributes, physiochemical properties, and PK parameters [32,33] of FDA-approved macrocyclic drugs since 2014.

Macrocyclic Drugs	Drug Class	MoA	MWt (g/mol)	logP	HBA	HBD	PSA (Å^2^)	RoA and Dosing Frequency	*T*max [t_1/2_] (h)	Bioavailability (%)	Protein Binding (%)	Vd (L)	Metabolism	Excretion (%)		Developed and/or Marketed by	Initial US Approval
														Urine	Feces		
Olysio (simeprevir) [DSC]	Antiviral (HCV)	NS3/4A protease inhibitor	749.94	6.10	12	2	194	150 mg capsule PO q24 h with food	4–6 h [10–13 h, healthy voluteers]; [41 h, HCV-infected patients]	62 (single dose under fed conditions)	>99.9		Primarily CYP3A4 via oxidative metabolism (possibly CYP2C8 and CYP2C19) to unchanged drug and metabolites (minor)	<1	91	Johnson & Johnson	22 November 2013 (discontinued on 25 May 2018) [34]
Dalvance (dalbavancin)	Antibacterial lipoglycopeptide	Inhibition of transpeptidation and cell wall synthesis	Mixture 1816.69	4.39	38	21	573	1500 mg IV (a single-dose) or 1000 and 500 mg IV (two doses 1 week apart)	[346 h]		93	7–13	hydroxy-dalbavancin (minor)	33 (P), 12 (M)	20	AbbVie Inc. (formerly by Allergan plc)	23 May 2014
Orbactiv (oritavancin) Kimyrsa (oritavancin)	Antibacterial lipoglycopeptide	Inhibition of transglycosylation and transpeptidation and disruption of bacterial membrane integrity	1793.10	3.84	36	22	561	1200 mg IV (a single-dose)	[245 h]		85	87.6	Not metabolized	<5 (P)	<1 (P)	Melinta Therapeutics, Inc.	6 August 2014 15 March 2021 for Kimyrsa
Vanihep (vaniprevir)	HCV	NS3/4A protease inhibitor	757.94	3.90	14	3	189	150 mg PO q12h								Merck Sharp & Dohme.	26 September 2014 (approved in Japan)
Viekira Pak or XR [DSC] (ombitasvir/ paritaprevir/ ritonavir/dasabuvir); Technivie ombitasvir/paritaprevir/ritonavir [DSC]	HCV	NS3/4A protease inhibitor	765.88	5.13	14	3	198	A fixed-dose combination product containing paritaprevir 50 or 75 mg PO two tablets (q24h)	4–5 h [5.5 h]	53	97–98.6	103 (Vss)	Metabolized by CYP3A4 and to a lesser extent CYP3A5	8.8	88	AbbVie Inc.	19 December 2014 (24 July 2015 for Technivie) (discontinued on 22 May 2018) [16]
Bridion (sugammadex)	Reversal agent for neuromuscular blockade	Antidote and selective relaxant binding agent	2002.15	−11.30	48	24	972	2, 4, or 16 mg/kg IV push as a single dose	[2 h] and prolonged in renal impairment		Negligible	11–14	Not metabolized	95 (P)		Merck & Co., Inc.	15 December 2015
Zepatier (elbasvir/grazoprevir)	HCV	NS3/4A protease inhibitor	766.90	4.37	15	3	204	One tablet (50 mg/100 mg) PO (q24h)	2 h [31 h]	27	98.8	1250	Hepatic (partial oxidative metabolism via CYP3A); metabolites not detected in plasma	<1	>90	Merck & Co. Inc.	28 January 2016
Trulance (plecanatide)	Chronic Idiopathic Constipation (CIC) and Irritable Bowel Syndrome with Constipation (IBS-C)	Guanylate cyclase-C agonist	1681.89	−2.81	44	26	819	3 mg PO once-daily		Minimal		Minimal	Proteolytic degradation (GI tract)			Salix Pharmaceuticals, Inc	19 January 2017
Vosevi (sofosbuvir/velpatasvir/voxilaprevir)	HCV	NS3/4A protease inhibitor	868.93	3.60	15	3	204	A fixed-dose combination tablet (400 mg/100 mg/100 mg) PO with food q24h	4 h [33 h]		>99					Gilead Sciences Inc	18 July 2017
Mavyret (glecaprevir/pibrentasvir	HCV	NS3/4A protease inhibitor	838.87	1.19	15	3	204	300 mg/120 mg PO q24h	5 h [6 h]		97.5		Secondary to CYP3A	0.7	92.1	AbbVie Inc.	3 August 2017
Moxidectin	Anthelmintic	binds to glutamate-gated chloride ions channels, gamma-aminobutyric acid (GABA) receptors, and/or APT-binding cassette transporters	639.82	7.50	9	2	116	8 mg PO as a single dose	4 h [23.3 days]			2421	Minimal		2 (P)	Medicines Development for Global Health	13 June 2018
Lorbrena (lorlatinib)	Anticancer (ALK+ metastatic NSCLC)	Anaplastic lymphoma kinase (ALK) inhibitor	406.41	0.78	8	2	110	100 mg PO once daily	1.2 h [24 h]	81	66	305 (Vss)	Primarily via CYP3A4 and UGT1A4, with minor contribution from CYP2C8, CYP2C19, CYP3A5, and UGT1A3	48 (<1, P)	41 (~9, P)	Pfizer Inc.	2 November 2018
Aemcolo (rifamycin SV)	Antibacterial	Protein synthesis inhibitor by binding to the β-subunit of bacterial DNA-dependent RNA polymerase	697.77	1.52	13	6	201	388 mg (two tablets) PO twice daily		<0.1	80		Not expected		86	Aries Pharmaceuticals, Inc.	16 November 2018
Vyleesi (bremelanotide)	Hypoactive sexual desire disorder	Melanocortin receptor agonist	1025.16	1.83	24	15	376	1.75 mg SubQ as one dose (maximum: 1.75 mg within 24 h). No more than 8 doses per month	1 h [2.7 h]	~100	21	25 ± 5.8	Primarily amide hydrolysis of the cyclic peptide	64.8	22.8	Palatin Technologies	21 June 2019
Imcivree (setmelanotide)	Obesity and the control of hunger associated with pro-opiomelanocortin deficiency	Melanocortin 4 (MC4) receptor agonist	1117.31	−0.08	27	20	495	Starting dose 2 mg SubQ q24h for 2 weeks, then 1 or 3 mg SubQ q24h	8 h [11 h]		79.1	48.7	Metabolized into small peptides by catabolic pathways.	39 (P)		Rhythm Pharmaceuticals, Inc.	25 November 2020
Lupkynis (voclosporin)	Immunosuppressant	Calcineurin inhibitor	1214.62	2.89	23	5	279	PO 23.7 mg (q12h)	1.5 h [30 h]		97	2154 (Vss/F)	Primarily hepatic via CYP3A4	2 (<1, P)	93 (5, P)	Aurinia Pharmaceuticals Inc.	22 January 2021
Netspot (gallium Ga 68 dotatate)	Diagnostic imaging agents	Somatostatin type 2 (sstr2) receptor binding agent	1503.56					2 MBq/kg (0.054 mCi/kg) of body weight up to 200 MBq (5.4 mCi) IV bolus	[1.1 h]			Extensive		12 (first 4 h)		Advanced Accelerator Applications	1 June 2016
Lutathera (lutetium Lu 177 dotatate)	Somatostatin receptor-positive gastroenteropancreatic neuroendocrine tumors (GEP-NETs).	Somatostatin type 2 (sstr2) receptor binding agent	1609.55					7.4 GBq (200 mCi) IV q8 weeks for a total of 4 doses	[71 ± 28 h]		43 (non-radioactive form)	460		Primarily renal		Advanced Accelerator Applications	26 January 2018
Detectnet (copper Cu 64 dotatate)	Diagnostic imaging agents	Somatostatin type 2 (sstr2) receptor binding agent	1497.55					148 MBq (4 mCi) IV bolus	[12.7 h]			Extensive		16–40 over 6 h		Curium US LLC	3 September 2020

Lipinski properties were obtained from Scifinder and calculated using Advanced Chemistry Development (ACD/Labs) Software V11.02 (© 2022–2021 ACD/Labs). MWt refers to the molecular weight of the free acid or base drug form. HBA, hydrogen bond acceptor; HBD, hydrogen bond donor; PSA; polar surface area. All PK parameters refer to adults unless otherwise noted. PO: by mouth or oral. IV: intravenous. Vd: volume of distribution. Under excretion: P refers to approximate % of total dose as parent drug and M refers to approximate % of total dose as metabolites. DSC: discontinued (brand name and/or generic product in the US).

## Abbreviations

ACA: azepane-2-carboxylic acid. cADPR: cyclic ADP-ribose mimic. ALK: anaplastic lymphoma kinase. CIC: chronic idiopathic constipation. CuAAC: copper-catalyzed azide–alkyne cycloaddition. DBU: 1,8-diazabicyclo[5.4.0]undec-7-ene. DCC: *N*,*N*′-dicyclohexylcarbodiimide. DCE: 1,2-dichloroethane. DCM: dichloromethane. DDC: sodium diethyldithiocarbamate. DIEA/DIPEA: *N*,*N*-diisopropylethylamine. DME: 1,2-dimethoxyethane. DMF: dimethylformamide. DMSO: dimethyl sulfoxide. DSC: discontinued. ESBL: extended spectrum β-lactamase. EDC: 1-ethyl-3-(3-dimethylaminopropyl)carbodiimide. FDA: Food and Drug Administration. GI: gastrointestinal. HATU: 2-(1*H*-7-azabenzotriazol-1-yl)-1,1,3,3-tetramethyl uronium hexafluorophosphate. HBA: hydrogen bond acceptor. HBD: hydrogen bond donor. HCTU: *O*-(1*H*-6-chlorobenzotriazole-1-yl)-1,1,3,3-tetramethyluronium hexafluorophosphate. HCV: hepatitis C virus. HFIP: hexafluoroisopropanol. HOAt: 1-hydroxy-7-azabenzotriazole. IBS-C: irritable bowel syndrome with constipation. IRAP: insulin-regulated aminopeptidase. IV: intravenous. LAH: lithium aluminum hydride. MBHA: 4-methylbenzhydrylamine. MCR: multi-component reaction. MICs: minimum inhibitory concentrations. MW: microwave. MWt: molecular weight. NMM: *N*-methylmorpholine. MTBE: methyl *t*-butyl ether. NT: neurotensin. NHS: *N*-hydroxysuccinimide. NMP: *N*-methylpyrrolidone. NSCLC: non-small cell lung cancer. PCSK9: proprotein convertase subtilisin/kexin type 9. PD: pharmacodynamic. PK: pharmacokinetic. PO: by mouth or oral. PRC2: polycomb repressive complex 2. PSA: polar surface area. PyAOP: (7-azabenzotriazol-1-yloxy)trispyrrolidinophosphonium hexafluorophosphate. q12h: every 12 h. q24h: every 24 h. RCM: ring-closing metathesis. PDCTC: 2, 6-pyridinedicarboxaldehydethiosemicarbazone. RGD: arginylglycylaspartic acid. RRCM: relay-ring-closing metathesis. RuAAC: ruthenium-catalyzed azide alkyne cycloaddition. SAR: structure–activity relationship. S_N_Ar: nucleophilic aromatic substitution. SPhoS: 2-dicyclohexylphosphino-2′,6′-dimethoxybiphenyl. sSPhos: sodium 2′-dicyclohexylphosphino-2,6-dimethoxy-1,1′-biphenyl-3-sulfonate hydrate. TBAF: tetra-*n*-butylammonium fluoride. TEA: triethylamine. TES: triethylsilane. TFA: trifluoroacetic acid. TIPS/TIS: triisopropylsilane. TMEDA: tetramethylethylenediamine. TPA: 12-*O*-tetradecanoylphorbol-13-acetate. Vd: volume of distribution.
